# A 6000-year-long genomic transect from the Bogotá Altiplano reveals multiple genetic shifts in the demographic history of Colombia

**DOI:** 10.1126/sciadv.ads6284

**Published:** 2025-05-28

**Authors:** Kim-Louise Krettek, Andrea Casas-Vargas, Alex Mas-Sandoval, Leonardo Arias Alvis, Ella Reiter, Julie Moncada Madero, Matthias Urban, Juan Camilo Niño Vargas, William Usaquén, Jose Vicente Rodríguez Cuenca, Cosimo Posth

**Affiliations:** ^1^ Senckenberg Centre for Human Evolution and Paleoenvironment at the University of Tübingen, Tübingen, Germany.; ^2^ Grupo de Genética de Poblaciones e Identificación, Instituto de Genética, Universidad Nacional de Colombia, Bogotá, Colombia.; ^3^ Dipartimento di Scienze Statistiche “Paolo Fortunati,” Universitá di Bologna, Bologna, Italy.; ^4^ Department of Evolutionary Genetics, Max Planck Institute for Evolutionary Anthropology, Leipzig, Germany.; ^5^ Leiden University Centre for Linguistics, Leiden, Netherlands.; ^6^ Archeo- and Paleogenetics, Institute for Archaeological Sciences, Department of Geosciences, University of Tübingen, Tübingen, Germany.; ^7^ Centre National de la Recherche Scientifique “Dynamique du Langage” (UMR5596) and Université Lumière Lyon 2, Lyon, France.; ^8^ Department of Anthropology, Faculty of Social Sciences, Universidad de los Andes, Bogotá, Colombia.; ^9^ Grupo de Investigación en Antropología Biológica, Departamento de Antropología, Universidad Nacional de Colombia, Bogotá, Colombia.; ^10^ Department of Archaeogenetics, Max Planck Institute for Evolutionary Anthropology, Leipzig, Germany.

## Abstract

Genetic studies on Native American populations have transformed our understanding of the demographic history of the Americas. However, a region that has not been investigated through ancient genomics so far is Colombia, the entry point into South America. Here, we report genome-wide data of 21 individuals from the Bogotá Altiplano in Colombia between 6000 and 500 years ago. We reveal that preceramic hunter-gatherers represent a previously unknown basal lineage that derives from the initial South American radiation. These hunter-gatherers do not carry differential affinity to ancient North American groups nor contribute genetically to ancient or present-day South American populations. By 2000 years ago, the local genetic ancestry is replaced by populations from Central America associated with the Herrera ceramic complex and survives through the Muisca period despite major cultural changes. These ancient Altiplano individuals show higher affinities to Chibchan speakers from the Isthmus of Panama than to Indigenous Colombians, suggesting a dilution of the Chibchan-related ancestry through subsequent dispersal events.

## Teaser

The hunter-gatherer gene pool of the Bogotá Altiplano was replaced by Central American ancestry at least 2000 years ago.

## INTRODUCTION

Genetic studies on ancient and present-day Indigenous populations have substantially contributed to the understanding of the settlement of the Americas. Those studies revealed that the population ancestral to non-Arctic Native Americans derives from a genetic admixture between ancient East Asian and Siberian groups somewhere in North-East Asia before 20,000 years before the present (yr B.P.) (*1*–*4*). Around 16,000 yr B.P., after its arrival in North America, this genetic ancestry split into two lineages known as northern Native American and southern Native American (*1*, *5*, *6*). While northern Native American ancestry is largely confined to ancient and current populations of North America, the southern Native American lineage expanded further south and constitutes the main ancestry component of ancient and present-day Indigenous South Americans. Southern Native American ancestry diversified within North America into at least three sublineages, i.e., one related to the Clovis-associated Anzick-1 individual from western Montana (USA), one found in ancient California Channel Islands individuals and the last one representing the main ancestry source of modern-day Central and South Americans (*7*–*9*). Each of these sublineages provided a wave of ancestry into the gene pool of ancient South Americans. Individuals from Chile and Brazil dating back to around 12,000 and 10,000 yr B.P., respectively, were more genetically related to the Anzick-1 genome than individuals from the eastern Southern American coast, Southern Cone and the Andes from 10,000 yr B.P. onward (*8*, *10*). In addition, the California Channel Islands ancestry was found in the Central Andes by 4200 yr B.P. and became widespread in the region thereafter (*8*, *11*). However, the exact timing of these population movements into the southern subcontinent remains largely unsolved to date.

The Isthmo-Colombian area, stretching from the coast of Honduras to the northern Colombian Andes, is critical to understanding the peopling of the Americas. Besides being the land bridge between North and South America, it is at the center of the three major cultural regions of Mesoamerica, Amazonia, and the Andes. At the time of European contact, the region was inhabited by a complex mosaic of human populations, mainly speakers of Chibchan, Chocoan, Carib, and Arawakan languages (*12*, *13*). Among those populations, those who were speakers of Chibchan languages were the most widespread in the region in terms of demography, cultural diversity, and territorial distribution (*13*–*15*). Chibchan is a language family with multiple, highly distinct branches, many of which are still spoken today in different regions of the Isthmo-Colombian area (*13*, *16*). The homeland and antiquity of the Proto-Chibchan language and the ancestor of all Chibchan languages remain subjects of debate. High intrafamily variation in terms of lexicon and grammar suggests that the language family is ancient and began diversifying several thousand years ago. The locus of that incipient diversification, however, is still uncertain (*17*). Most scholars believe that this protolanguage began to diversify in Lower Central America, where the largest number of these languages is spoken today (*18*). However, some evidence suggests that Proto-Chibchan might have originated in South America and then diversified in Central America at a much later date (*14*). Genetic studies of ancient and present-day Isthmo-Colombian Indigenous populations revealed a distinctive ancestry component primarily associated with speakers of Chibchan languages (*19*, *20*). However, whereas mitochondrial DNA (mtDNA) studies suggested a migration of Chibchan-related ancestry from Central America into Colombia and Venezuela (*21*, *22*), genome-wide studies favored an opposite, south-to-north population movement (*23*, *24*). According to the latter model, speakers of Chibchan languages from Central America are not direct descendants of the first settlers in the region but, instead, derive from a more recent back migration from South to Central America (*23*).

The southernmost region of the Isthmo-Colombian area is the Altiplano Cundiboyacense (hereafter Altiplano). This plateau with an average altitude of 2600 m in the Eastern Cordillera of the Colombian Andes was inhabited by ancient hunter-gatherer groups from the Late Pleistocene (*25*, *26*). During the Early and Middle Holocene phases of the Preceramic period (~11,500 to 4000 yr B.P.), populations on the Altiplano underwent multiple cultural transformations, most notably increased sedentism and a transition from a hunter-gatherer subsistence to the introduction of horticultural practices and forest management (*27*). However, it was not until the early Late Holocene, ~3800 yr B.P., that the first clear evidence of maize cultivation appeared (*28*).

During the subsequent Formative period (~3000 to 1000 yr B.P.), a distinct type of pottery emerged on the Altiplano that is referred to as the Herrera ceramic complex, also known in the literature as the Herrera period (2800 to 1200 yr B.P.) (*29*). It is still highly debated whether Herrera-associated groups on the Altiplano derived from an in situ development of local hunter-gatherers or were a consequence of population dispersals into the region (*30*, *31*). Around 1200 yr B.P., a cultural phase, known as the Muisca period, began on the Altiplano and lasted until the imposition of the Hispanic Colonial regime in the mid-16th century (*27*, *32*). Most available evidence is suggestive of population continuity with the preceding Herrera period (*33*, *34*). The Muisca period is characterized by a relatively continuous process of demographic growth, development of agriculture and trade, and social and political complexification. These factors played a considerable role in shaping the Muisca culture and gave rise to the Chibchan-speaking population that dominated the Altiplano until European colonization.

While several studies have reported mtDNA data from ancient Colombian individuals (*19*, *31*, *35*, *36*), genome-wide data from this region are still entirely lacking to date. In this study, we generated mtDNA and genome-wide data of 21 ancient individuals from two areas of the Altiplano (Bogotá plateau and Los Curos). Our data, spanning a time transect between around 6000 and 500 yr B.P., provide an opportunity to explore several key questions: (i) Which southern Native American genetic ancestry do Preceramic individuals from the Altiplano derive from? (ii) Were the cultural transformations associated with the Herrera and Muisca periods accompanied by migrations and demographic changes? (iii) How is the genetic ancestry observed in speakers of Chibchan languages related to that of ancient individuals from the Altiplano? (iv) What are the genetic relationships between the generated ancient genomes and the existing genomic data of present-day Indigenous communities from Colombia and neighboring regions?

### Ethics statement

We acknowledge that this ancient DNA (aDNA) study focused on pre-Hispanic individuals could potentially have profound implications for present-day Indigenous communities. Therefore, we established contact with the Guardia Indígena Muisca, representatives of the Muisca people of Suba and the Resguardo de Suba located in the Bogotá plateau, to discuss the research results and present them in a way that respects local history, values, and traditions. This effort will extend beyond publication, with coordinated joint dissemination and outreach activities. During our consultation with community members and presentation to local archaeologists and anthropologists, we stressed that findings on the genetic ancestry of ancient individuals can be refined through additional analyses and/or data. Moreover, we emphasized that genetic results are independent and should not be conflated with concepts of cultural identity. As scientists addressing questions of relevance to Indigenous communities from Colombia, we respect and value the richness of community-based knowledge.

## RESULTS

### Dataset and aDNA authentication

**Fig. 1.**
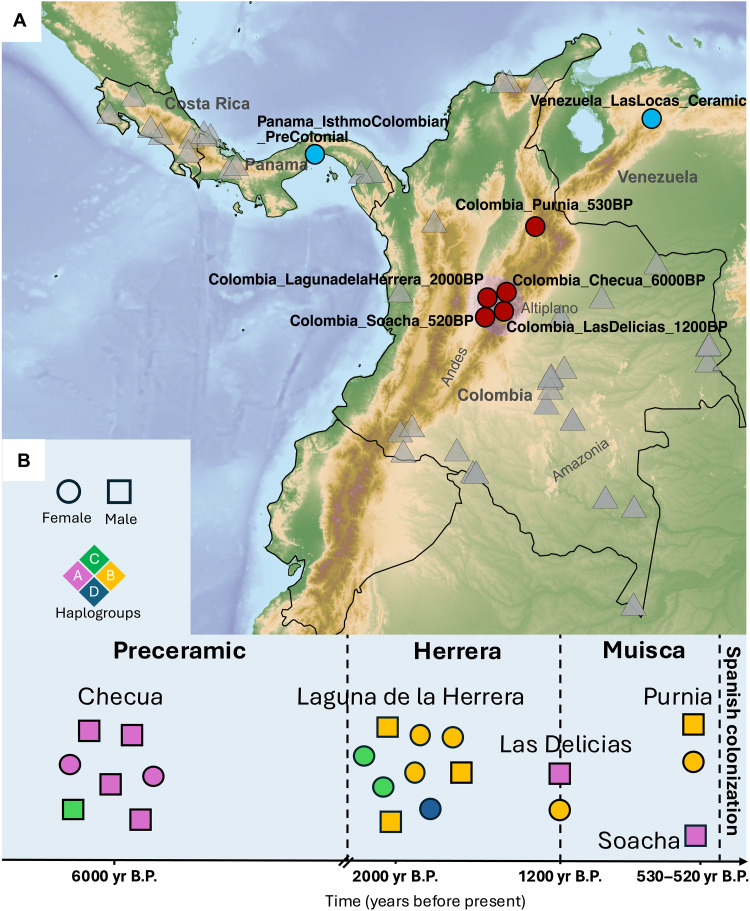
Geographic and temporal framework of the analyzed ancient and modern individuals. (**A**) Map of the Isthmus of Panama (thereafter isthmus) and northwestern South America indicating the geographic location of modern-day Indigenous populations from Colombia, Panama, and Costa Rica (gray triangles), ancient Colombians (red circles), Ceramic-age Venezuelans and ancient Panamanians (light blue circles). (**B**) Timeline of the generated ancient genomes from the Altiplano and associated archaeologically defined periods. The Herrera ceramic complex is part of the Formative period. Individual dates are reported as scattered around the median of calibrated radiocarbon dates obtained from direct dating of individuals within the groups (table S1).

We report data from five archaeological sites on the Altiplano encompassing an ancient genomic time transect of around 5500 years (Fig. 1A). The generated dataset includes seven hunter-gatherer individuals from the Preceramic period (Colombia_Checua_6000BP) (fig. S1), nine individuals from the Herrera period (Colombia_LagunadelaHerrera_2000BP) (fig. S2), three individuals associated with both initial and final phases of the Muisca culture (Colombia_LasDelicias_1200BP and Colombia_Soacha_520BP) (figs. S3 and S4) all from the Bogotá plateau, and two individuals from further north in the Los Curos area associated with Guane populations (Colombia_Purnia_530BP) (fig. S5). To obtain these data, we extracted DNA from the petrous portion of the temporal bone and teeth, generated double-stranded genetic libraries and captured them for both the complete mtDNA and a targeted set of ~1.24 million genome-wide single-nucleotide polymorphisms (1240K SNPs). We sequenced the libraries on Illumina platforms, resulting in an SNP coverage between 177,102 and 912,488 SNPs (average 541,067). We further evaluated aDNA authenticity through mtDNA and X-chromosome contamination estimates, which were found to be in all cases below 4 and 5%, respectively (table S1). We projected the generated ancient genome-wide data onto a principal components analysis (PCA) built with the variation over the 1240K SNPs of worldwide modern-day populations (table S8A) (*37*). All individuals fall within the expected variation of modern-day Native Americans, further confirming the absence of substantial contamination with non-Native American ancestries (fig. S6).

### Uniparental markers and population structure

Genetic sexing revealed the presence of 10 females and 11 males in our dataset, all the latter belonging to Y-chromosome haplogroup Q1b1a and its subclades (table S1). This is the predominant paternal haplogroup in Native Americans (*38*, *39*), and our data confirm a high frequency within ancient Colombia (table S1). The most prevalent mtDNA haplogroups in modern-day Indigenous Colombians are A2, B2, and C1, while D1 and D4h3a are substantially less common (*19*). This general trend is confirmed in our ancient sample set, where haplogroup B2d has the highest frequency, followed by A2 and C1, while only one individual carries haplogroup D1 (Fig. 1B). The highest proportion of haplogroup A2 is found in the group Colombia_Checua_6000BP, as previously shown for older individuals from the same site (*36*).

We estimated genetic kinship among all individuals (*40*) as well as distant relatedness and levels of inbreeding using states of identity by descent (IBD) and runs of homozygosity (ROHs) (*41*). While the former describes close family relatedness between individuals (up to third degree), IBD and ROH inform about shared ancestors, consanguinity, and effective population size. We detected four second- and third-degree relationships in individuals of the Colombia_Checua_6000BP group (table S1). Five of the seven individuals sequenced from this site are males, with related individuals sharing the same Y-chromosome haplogroup but not mtDNA haplogroups. The analyzed individuals from Colombia_LagunadelaHerrera_2000BP are less related with only one parent-offspring relation (FORM006 and FORM004) and another second-degree relation (FORM004 and FORM008). Furthermore, we do not find evidence for routinely practiced close-kin mating patterns, with a rare sharing of long IBD segments between the male individuals (figs. S7 and S8). ROHs over 4 cM were present in all individuals, with the highest prevalence in the Colombia_Checua_6000BP group (fig. S9). The distribution of ROHs suggests the effective population size for Colombia_Checua_6000BP to be between three and eight times smaller than later ancient Colombian groups. This fits archaeological descriptions of small population sizes within hunter-gatherer communities on the Altiplano, followed by a steep demographic growth during the Herrera and Muisca periods (*42*, *43*).

For subsequent population genetic analyses, we clustered individuals in groups based on their date and archaeological site, after removing first- and second-degree relatives and confirming the absence of genetic outliers (table S2, A and B). Last, we combined these data with available ancient and present-day individuals from the Americas (see Materials and Methods).

### Preceramic hunter-gatherers derive from a previously unknown basal lineage

**Fig. 2.**
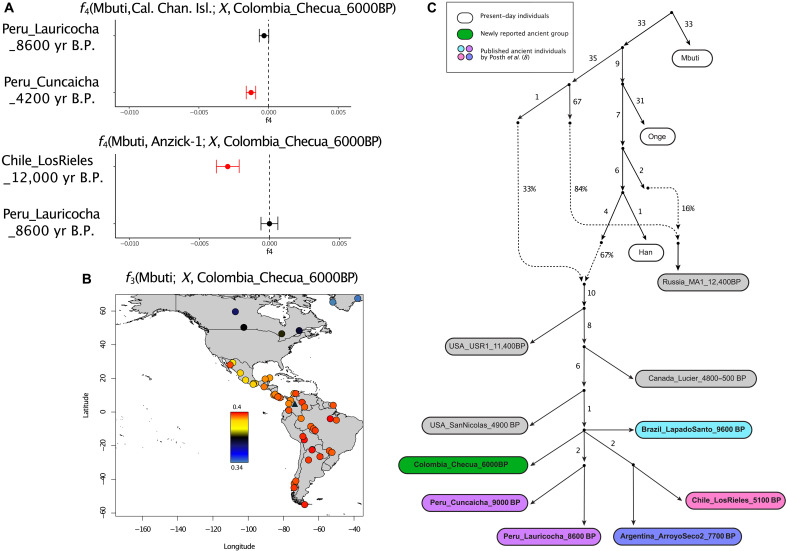
Genetic characterization of the preceramic hunter-gatherers from Checua. (**A**) *f*_4_ statistics measuring the differential affinity of Colombia_Checua_6000BP to distinct ancient southern Native American lineages related to the California Channel Islands and Anzick-1 genomes. Tests are reported with 1 SE, and red symbols indicate *z*-scores below −3. (**B**) Map with *f*_3_ outgroup statistics measuring the shared genetic drift of Colombia_Checua_6000BP with present-day Indigenous American populations genotyped on the Illumina dataset (colored circles). The location of the Checua site is indicated with a black triangle. (**C**) Admixture graph that comodels major North and South American lineages including Colombia_Checua_6000BP (worst *f*_4_ statistic *z*-score = 3.2).

To determine which specific branch of southern Native American ancestry the Altiplano’s hunter-gatherers derived from, we used a series of *f*_4_ statistics. We designed these analyses taking into account that Chile_LosRieles_12000BP maximizes the Anzick-1 ancestry in South America, Peru_Cuncaicha_4200BP is among the individuals with highest California Channel Islands ancestry, and Peru_Lauricocha_8600BP does not present either of these two ancestries (*8*). We found that Colombia_Checua_6000BP does not present higher genetic affinity to Anzick-1 than Peru_Lauricocha_8600BP, while Chile_LosRieles_12000BP is more closely related to Anzick-1 than Colombia_Checua_6000BP (Fig. 2A and table S3A), indicating the absence of significant Anzick-1–related ancestry in the analyzed hunter-gatherers from the Altiplano. Similarly, *f*_4_ statistics reveal that Colombia_Checua_6000BP does not carry a distinct affinity to the ancient California Channel Islands individuals (Fig. 2A and table S3B).

Our combined findings show that Colombia_Checua_6000BP does not belong to either the Anzick-1–related or California Channel Islands–related expansions into South America and is consistent with deriving from the lineage that represents the primary source of ancestry of South Americans (*8*). This lineage was shown to have a long-standing genetic continuity until present-day individuals in multiple regions of South America from at least 9000 years ago (*8*). Therefore, we tested whether Colombia_Checua_6000BP shows differential affinity to any ancient and present-day population from South America with the *f*_3_ outgroup statistics of the form *f*_3_(Mbuti, modern/ancient Native American; Colombia_Checua_6000BP). This analysis reveals that Colombia_Checua_6000BP shares higher genetic drift with populations from Central and South America than North America but does not show specific allele sharing with any Central and South American population (Fig. 2B), consistent with Colombia_Checua_6000BP deriving from a deeply divergent South American lineage. We formally investigated this assumption with a set of *f*_4_ statistics of the form *f*_4_(Mbuti, Colombia_Checua_6000BP; modern/ancient Native American, modern/ancient Native American) testing for potential distinct affinities between the ancient hunter-gatherer group and ancient or present-day Native American individuals available in our dataset (table S3, C to E). As observed in *f*_3_ outgroup statistics, significant affinities are only observed when Central and South American individuals are compared against North Americans. Instead, *f*_4_ statistics are found to be consistent with zero for comparisons between ancient and modern-day Central and South American populations, respectively.

Combined, these results suggest the positioning of Colombia_Checua_6000BP as an outgroup to virtually most Indigenous South Americans. To formally test this placement, we modeled a qpGraph tree that recapitulates major demographic events in the settlement history of the Americas (*8*). When placing Colombia_Checua_6000BP in any possible position of the admixture graph, we obtained a statistically robust fit only when this group is modeled as a previously uncharacterized lineage deriving directly from the initial radiation of Central and South American populations (Fig. 2C and fig. S10) (*5*, *8*).

Last, we analyzed our post-2000–yr B.P. ancient genomic data from the Altiplano to investigate the presence of possible local genetic continuity. With *f*_4_ statistics, we are able to show not only that Colombia_Checua_6000BP is equally distant from all subsequent ancient groups in the Altiplano [*f*_4_(Mbuti, Colombia_Checua_6000BP; ancient Colombia 1, ancient Colombia 2) = 0] but also that this group is not more closely related to subsequent ancient Colombians than to any other South American population in our dataset [*f*_4_(Mbuti, Colombia_Checua_6000BP; ancient Colombia, modern/ancient Native American) = 0]. Overall, the performed analyses provide robust evidence for a major genetic replacement on the Altiplano between 6000 and 2000 years ago (table S3, F to H).

### Genetic replacement on the Altiplano by at least 2000 years ago

**Fig. 3.**
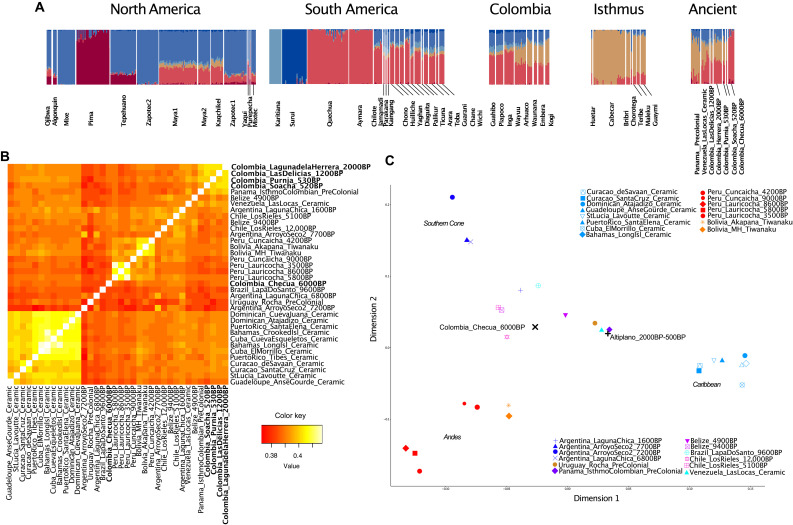
Population affinities of ancient Colombian individuals with ancient South Americans. (**A**) ADMIXTURE analysis including present-day Native American individuals genotyped on the Illumina panel and ancient individuals from Panama, Venezuela, and Colombia (*K* = 6). (**B**) Heatmap of *f*_3_ outgroup statistics between representatives of distinct ancient lineages among Central and South American groups. (**C**) MDS plot based on 1 − *f*_3_ outgroup statistics including the same populations as in (B) but with post-2000–yr B.P. ancient Colombians combined into a group called Altiplano_2000–500BP. Axes of genetic variation are labeled on the basis of their geographic origin in italic font.

To gain an overview of the genetic composition of all sequenced ancient individuals from the Altiplano, in comparison with other ancient populations from the broader region and present-day Native Americans, we ran an unsupervised ADMIXTURE analysis including only individuals where non-Native ancestries were either not present or masked (Fig. 3A and fig. S11). The results of this analysis confirm that the genetic profile of Colombia_Checua_6000BP is substantially different from all subsequent ancient Colombians. In turn, the Altiplano individuals spanning between 2000 and 500 yr B.P. show a highly similar genetic composition. Beside the ancestry components seen also in Colombia_Checua_6000BP, these younger individuals carry a large proportion of an ancestry found in major amounts in ancient individuals from Panama dated between 1400 and 600 yr B.P. (*20*). A similar ancestry profile is also observed in ceramic-associated individuals from Venezuela dated to ~2400 yr B.P. (*44*), although with a greater intrasite variability.

To assess whether the individuals from the Altiplano dated between 2000 and 500 yr B.P. show evidence of genetic continuity through time, we perform a series of analyses based on *f* statistics. First, we computed an *f*_3_ outgroup heatmap with representative ancient groups of distinct Central and South American ancestries. This analysis shows that ancient individuals from the Altiplano dated to the Herrera and Muisca periods form a cluster with high genetic affinity (Fig. 3B). This is further supported by *f*_4_ statistics and qpWave analysis (tables S4, A to G, and S5, A to J), providing further evidence that individuals from Colombia_LagunadelaHerrera_2000BP, LasDelicias_1200BP, and Soacha_520BP, all from the Bogotá plateau, carry a common genetic ancestry. In addition, the Colombia_Purnia_530BP group in the Los Curos area of the Altiplano is highly similar to our post-2000–yr B.P. ancient genomic time transect in the Bogotá plateau, confirming the previously observed genetic link between both regions (*35*). Collectively, these findings reveal a large genetic continuity on the Altiplano that persisted for at least 1500 years, despite major cultural transformations between the Herrera and Muisca periods.

We then attempted to characterize the source of the genetic ancestry that reached the Altiplano by at least 2000 years ago. We first built a multidimensional scaling (MDS) plot based on *f*_3_ statistics including populations that represent major axes of genetic variation discovered in Central and South America thus far (Fig. 3C). Because of the high genetic affinity shown before, for this analysis, we combined all post-2000–yr B.P. ancient Colombian individuals in a single group called Altiplano_2000–500BP. In the MDS plot, we identify three genetic clusters represented by ancient individuals from the Andes, Southern Cone, and Ceramic-age Caribbean, respectively (*8*, *44*, *45*). Colombia_Checua_6000BP falls in a central position of the MDS plot, while the Altiplano_2000–500BP group is close to both pre-Hispanic individuals from Panama and Ceramic-associated individuals from Venezuela, in agreement with the ADMIXTURE analysis. Direct *f*_4_ statistics comparing ancient Colombians against ancient Native American genomes confirm notable affinities of ancient Colombians younger than 2000 yr B.P. and ceramic-associated Venezuelans with ancient Panamanians (table S4, D to G).

To investigate the genetic relationships between ancient Colombians, Venezuelans, and Panamanians, we built two PCAs zooming in on the genetic diversity of central and northern South Americans onto which ancient data were projected (figs. S12 and S13). Our findings demonstrate that both ancient Colombians and Venezuelans fall in an intermediate position between ancient Panamanians and other previously detected ancestries in Central and South America. Notably, Ceramic-associated individuals from a single site in Venezuela are more spread out in PCA space than post-2000–yr B.P. ancient individuals from four sites in Colombia.

We further explored the branching and admixture patterns between ancient Central American and northern South American genomes by performing a TreeMix analysis (figs. S14 to S19). Because of the previously shown heterogeneity of the Ceramic-associated individuals from Venezuela, we used *f*_4_ statistics to test for differential affinity of specific individuals to other ancient Native American populations (table S6, A to D). These tests revealed that Venezuela_LasLocas_Ceramic_2 (three individuals) has a significantly higher affinity to ancient Panama than Venezuela_LasLocas_Ceramic_1 (five individuals). With TreeMix, we reveal a consistent pattern where Venezuela_LasLocas_Ceramic_2 is always closer to ancient Panama than any post-2000–yr B.P. ancient Colombian group, while any post-2000–yr B.P. ancient Colombian group is always closer to ancient Panama than Venezuela_LasLocas_Ceramic_1 (figs. S14 to S19). Ancient Colombian groups never form a clade with either of the ancient Venezuelan groups to the exclusion of ancient Panama. The sparse temporal sampling does not allow us to estimate if the Panamanian-related ancestry reached Colombia and Venezuela at similar times, but our results show that Ceramic-associated individuals from those two neighboring countries underwent distinct demographic processes.

We then tested whether ancient Colombians and Venezuelans are on an admixture cline between a Panamanian-related ancestry and another sources with *f*_4_ statistics of the form *f*_4_(ancient outgroup capture, *X* capture; Altiplano_2000–500BP/ancient Venezuela, ancient Panama shotgun) and *f*_4_(ancient outgroup shotgun, *X* shotgun; Altiplano_2000–500BP/ancient Venezuela, ancient Panama shotgun). Although the results from PCA and ADMIXTURE would be consistent with the presence of such an admixture cline, *f*_4_ statistics do not show any significant statistics when accounting for biases introduced by different sequencing protocols such as shotgun sequencing and targeted enrichment (table S6, E to I). One possibility is that our comparative dataset lacks an appropriate proxy for the source population that mixed with the Panama-related ancestry and gave rise to post-2000–yr B.P. ancient Colombian populations. Alternatively, it is also plausible that the incoming Panama-related ancestry did not mix with other genetic sources during its expansion to the Altiplano. In this scenario, the genetic differences between ancient Panamanians on one side and ancient Colombians and Venezuelans on the other might be due to a process of postsplit genetic drift in one or multiple of these lineages.

### Genetic affinities with modern-day Native Americans

Previous research has revealed a high level of genetic similarity between pre-Hispanic Panamanian individuals and speakers of Chibchan languages, particularly those from Costa Rica and Panama (*20*). We first investigated whether ancient Panamanians and ancient Colombians are equally related to present-day Chibchan speakers from Central America with *f*_4_(Mbuti, modern isthmus; ancient Panama, ancient Colombia) < 0. As expected, we found a higher level of allele sharing through time between Panama and Costa Rica rather than between Panama and Colombia (table S7A). This result aligns with the pattern observed in ADMIXTURE where ancient Panamanians have a much higher proportion of an ancestry component maximized in modern-day Chibchan speakers in comparison to ancient Colombians (Fig. 3A).

**Fig. 4.**
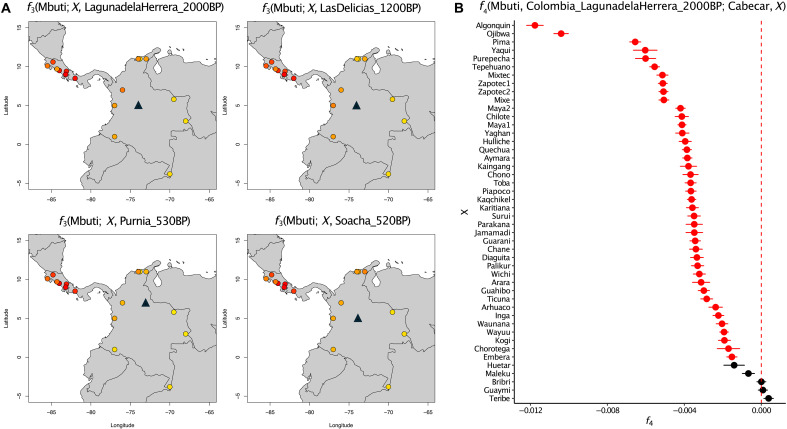
Genetic similarity of post-2000–yr B.P. ancient Colombians with present-day Native American populations. (**A**) *f*_3_ outgroup statistics between post-2000–yr B.P. ancient Colombians and present-day Indigenous populations from Costa Rica, Panama, and Colombia genotyped on the Illumina panel (colored circles). The location of the Colombian archaeological sites is indicated with a black triangle in each panel. (**B**) *f*_4_ statistics to test the relative affinity of Colombia_LagunadelaHerrera_2000BP compared to Cabécar (a modern Lower Central American population) and other present-day Native American populations genotyped on the Illumina panel. Tests are reported with 1 SE, and red symbols indicate *z*-scores below −3.

We then tested whether the link between ancient Panamanians and ancient Colombians extends into modern-day populations from the isthmus, using unadmixed individuals or individuals where recent admixture with non-Native American ancestries have been masked. *f*_3_ outgroup statistics of the form *f*_3_(Mbuti, ancient Colombia; present-day Native American) show the highest affinity of ancient Colombian groups younger than 2000 yr B.P. to Chibchan-speaking populations from Central America (Fig. 4A). Higher levels of shared genetic drift are observed with present-day populations from the isthmus rather than Native American populations from Colombia, some of whom also speak Chibchan languages (Fig. 4A and fig. S20). We confirm this pattern with two sets of *f*_4_ statistics. With *f*_4_(Mbuti, modern Native American; ancient Colombia 1, ancient Colombia 2) = 0, we show that there is no evidence of differential affinity of either Colombian group to modern-day populations within or outside the isthmus, confirming the homogenous genetic profile in the Altiplano at least from 2000 yr B.P. until colonization (table S7, B to G). Instead, *f*_4_(Mbuti, ancient Colombia; modern Native American, modern Lower Central America) > 0 shows that speakers of Chibchan languages from Central America (such as Cabécar) are significantly closer to post-2000–yr B.P. ancient Colombians than any other present-day population in our dataset is (Fig. 4B and fig. S21).

This finding is true even when comparing Chibchan-related populations from the isthmus against northern Colombian groups (Fig. 4B and table S7H). However, present-day Colombian groups are not all equally related to post-2000–yr B.P. ancient Colombians. Specifically, Indigenous populations from northern and western Colombia have higher affinity to ancient Colombians than Indigenous populations from other Colombian regions, as shown with *f*_4_(Mbuti, ancient Colombia; modern Colombia 1, modern Colombia 2) (table S7D). While some of these northern and western Colombian populations do speak Chibchan languages (i.e., Kogi, and Arhuaco), others do not (i.e., Embera, Waunana, and Wayuu), and their shared genetic affinity with post-2000–yr B.P. ancient Colombians might be due to the geographic proximity to Chibchan-speaking populations, as previously shown with present-day genotype data (*23*, *24*).

Genetic links can be attributed to the extensive cultural contacts across the Isthmo-Colombian area, as attested for example by the distribution of some recurrent motifs in gold and tumbaga ornaments (*13*). Furthermore, archaeological evidence suggests that in pre-Columbian times, speakers of Chibchan languages occupied a broader geographic area in Colombia, including portions of the Cauca Valley and the Sinú region where a prolonged interaction took place with speakers of Chocoan languages (*46*).

## DISCUSSION

In this study, we generated genome-wide data from 21 individuals spanning a time transect of almost 6000 years from the Altiplano, which represents the southern edge of the Isthmo-Colombian area. Our findings contribute to a better understanding of the population history of this area, a key region in the peopling process of South America. We show that the hunter-gatherer population from the Altiplano dated to around 6000 yr B.P. lack the genetic ancestry related to the Clovis-associated Anzick-1 genome and to ancient California Channel Island individuals, suggesting their affiliation to the southern Native American lineage that became the primary source of ancestry of South Americans by 9000 yr B.P. (*8*). However, unlike ancient genomes from the Andes and the Southern Cone that are associated with the same wave of ancestry, the analyzed Preceramic individuals from Colombia do not share distinct affinity with any ancient or modern-day population from Central and South America studied to date. Colombia_Checua_6000BP can thus be modeled as a previously undescribed distinct lineage deriving from the radiation event that gave rise to multiple populations across South America during its initial settlement.

The cultural transition between the Preceramic and Herrera periods is associated with a seemingly complete replacement of the local genetic profile. This challenges the model where local hunter-gatherers developed in situ as suggested by morphometric studies and an ancient mtDNA time transect (*31*, *47*). Instead, our study provides evidence for a major genetic turnover on the Altiplano occurring after 6000 yr B.P. but before 2000 yr B.P. Since the mechanisms and precise temporal scale of this replacement event remain uncertain, we cannot directly associate it with the emergence of maize cultivation ~3800 yr B.P. However, our data do support the archaeological hypothesis that the introduction of pottery associated with the Herrera ceramic complex was mediated through population dispersals (*25*, *48*).

Our results show that the incoming genetic ancestry on the Altiplano is related to ancient and present-day populations speaking Chibchan languages from Central America. This can be explained most parsimoniously by Chibchan-related migrations from Lower Central America to South America, rather than back-migration to the isthmus. A separate study found evidence for a previously unknown south-to-north expansion of Chibchan-related ancestry from Lower Central America into the Mayan territories of Belize by 5600 yr B.P. (*49*). Therefore, rather than modeling Central American populations associated with Chibchan languages as deriving from a mixture between North and South American ancestries (*23*), these results are consistent with an origin of Chibchan-related ancestries in Lower Central America, followed by bidirectional gene flow toward both Meso- and South America. This model of an original “Chibchan homeland” in Central America is supported not only by mtDNA studies on present-day populations who speak Chibchan languages but also from linguistic observations, indicating that the isthmus region exhibits the highest diversity within this language family (*17*, *22*).

From an archaeological perspective, the Chibchan-related ancestry is first identified in 2000-year-old individuals associated with Herrera ceramics. In addition, previously sequenced Ceramic-associated individuals from Venezuela dated to 2400 yr B.P. also showed a high affinity to Central American populations speaking Chibchan languages (*44*). Despite the similar ancestry pattern and temporal frame, the two populations do not appear to form a simple sister group. This could be in line with linguistic evidence that suggests multiple, distinct Chibchan language expansions into South America (*13*), but additional studies will be necessary to further clarify this issue.

After the arrival of the Chibchan-related ancestry, which completely reshaped the genetic landscape of the region, we find evidence of a long period of genetic continuity in the genetic profile of the local populations for over 1500 years (from at least 2000 to 500 yr B.P.). The stability in genetic ancestry encompasses the end of the Herrera period and the beginning of the Muisca period. This points to a scenario in which populations speaking languages from the Chibchan lineage would have settled the Altiplano before the emergence of traits normally associated with the Muisca culture, and it shows that this cultural transition took place without a substantial migration from regions with a distinct genetic ancestry composition. In addition, such a genetic continuity extends through different cultural phases within the Muisca period and persists until the Spanish colonization. Colonial linguistic documentation established that Muisca people spoke a now extinct Chibchan language. Our findings not only confirm their genetic link with speakers of Chibchan languages from Central America but also suggest that ancestral Chibchan languages, possibly basal to the Magdalenic branch that gave rise to the documented Muisca language, might have already been spoken on the Altiplano during the pre-Muisca Herrera period.

While the representation of Indigenous populations in our dataset is certainly not exhaustive, the observed spatial pattern in the genetic affinity of post-2000 yr B.P. ancient Colombians with present-day Indigenous populations raises questions regarding the uneven distribution of populations speaking Chibchan languages across the Isthmo-Colombian area at the time of the Hispanic colonization, also referred to as a Chibchan “archipelago” (*13*).

One possible explanation is that this distribution resulted from separate dispersals from Central America to different locations of northern South America rather than a single expansion wave, as suggested by the internal branching pattern of the Chibchan language family (*13*, *18*). However, it is also possible that the initial spread was more widespread and got later fragmented by post-Chibchan migration and admixture events. The observation that Chibchan-affiliated populations from northern Colombia have a significantly reduced genetic affinity to post-2000–yr B.P. ancient Colombians than to Lower Central Americans supports the role of population admixture in shaping the genetic diversity of northern South America.

Although this study largely enriches our understanding of the complex population history of the Isthmo-Colombian area, we highlight three main limitations. First, this study reports genome-wide capture data from ancient Native American populations, but the vast majority of the variation in the 1240K SNP capture panel is fixed in Native American populations. This limits the resolution of our analyses, and future efforts should be devoted to whole genome sequencing and/or target enrichment of American-specific SNP panels to increase statistical power and analysis complexity. Second, a denser genomic time transect on the Altiplano could narrow down the timing for the arrival of the Chibchan-related ancestry and potentially identify its admixture with a yet uncharacterized ancestry source. Third, since this is a regionally focused study, it is important to assess whether the described population transformations also influenced other regions beyond the Altiplano, both for areas with and without recorded evidence of Chibchan languages. Ancient genomic data from neighboring areas along the Northern Andes that have not yet been analyzed through ancient genomics, such as western Colombia, western Venezuela, and Ecuador, will be pivotal to better define the timing and ancestry sources of human migrations into South America.

## MATERIALS AND METHODS

### Archaeological sampling

Permission to perform genetic research at the University of Tübingen (Germany) on ancient individuals from the Altiplano housed in the anthropological collection of the National University of Bogotá was granted by the Colombian Institute of Anthropology and History, with the authorization for archaeological intervention no. 8826. We processed 21 individuals from five sites in a dedicated aDNA laboratory at the University of Tübingen: Checua (*n* = 7), Laguna de la Herrera (*n* = 9), Las Delicias (*n* = 2), Purnia (*n* = 2), and Soacha (*n* = 1). Detailed descriptions of the archaeological context at each site can be found in Supplementary Text. Each petrous portion of the temporal bone or tooth sample was photographed from multiple angles and micro–computed tomography (microCT) scanned before sampling. Teeth were processed by cutting at the dentin-enamel junction using an electric saw blade. We generated dentine powder from the inside of the crown using an electric dental drill. Petrous bones were sampled by drilling into the cochlea from the internal acoustic meatus after removing the first layer of bone surface. For each sample, 50 to 55 mg of bone powder was generated.

### Extraction and library preparation

The bone powder was used for DNA extraction. To digest the bone matrix, 1 ml of a buffer containing 0.45 M EDTA (0.5 M at pH 8), ultraviolet (UV)–treated water, and Proteinase K (0.25 mg/ml) was added to each sample. This was followed by a 24-hour incubation step at 37°C with constant rotation (*50*). The undigested remains of bone powder were pelleted, and the supernatant transferred into a tube containing 10 ml of UV-treated binding buffer (5 M guanidine hydrochloride, 40% isopropanol, and UV-treated H_2_O) and 400 μl of 3 M sodium acetate (pH 5.2). Each sample was transferred into a 50-ml Falcon tube containing a silica column for high volumes and purified (High Pure Viral Nucleic Acid Large Volume Kit, Roche). The purified DNA was eluted in 2× 50 μl of tris-EDTA-Tween (TE buffer and 0.05% Tween) and stored in a dedicated freezer at −20°C.

We used 25 μl of DNA to construct double-stranded, double-indexed libraries, implementing a partial uracil-DNA glycolase (half-UDG) protocol (*51*). Two indices per sample were selected by an in-house software to create a unique combination for each library. All libraries were initially amplified via polymerase chain reaction for 10 cycles and purified using MinElute columns in combination with QIAGEN reagents. Afterward, libraries were reamplified with library-specific cycles to aim for a concentration of 1.5 × 10^13^ copies per library before purification. Afterward, all libraries were quantified on the Agilent 4200 TapeStation System following the manufacturer’s protocol and pooled equimolarly (10 nM) for shallow shotgun sequencing. Single-end sequencing was performed on an Illumina HiSeq 4000 for ~5 million reads per library.

### Data processing, capture, and authentication

To assess the presence of aDNA, we considered the percentage of human DNA, the average fragment length, and the damage pattern at the 5′ end of the molecules. This screening process was performed with the software EAGER version 1.92.38 and integrated programs (*52*). The adapters were removed using AdapterRemoval with its default settings (*53*). Afterward, reads were aligned to the human reference genome hg19 (GRCH37) using the Burrows-Wheeler Aligner with disabled seed length, a mismatch parameter of 0.01 and a quality filter of 30 (*54*). Afterward, duplicates were removed with DeDup, and damage patterns were inferred using mapDamage2.0 (*55*). To include a sample for in-solution target enrichment, we set a preservation threshold of 0.1% of human DNA and a minimum of 5% terminal damage. The selected libraries were then reamplified and enriched for a targeted set of 1240K SNPs across the human genome. In addition, we captured each library for the human mitochondrial genome. Both target enrichments were performed in solution using a modified version of Fu *et al.* (*56*). Single-end sequencing was performed on an Illumina HiSeq 4000 between ~30 and 80 million reads for each 1240K captured library.

Additional authentication steps were implemented on captured data by estimating mtDNA as well as X-chromosomal contamination levels. MtDNA contamination was established using schmutzi (*57*), and X-chromosomal contamination was estimated using Angsd (*58*) for individuals genetically determined male (table S1).

### Genotyping, sexing, and haplogroup calling

Before calling variants, two base pairs at both reads termini were trimmed using the samtools trim package (https://github.com/samtools/samtools). Afterward, the pseudohaploid genotypes were called using a random calling approach with pileupCaller (https://github.com/stschiff/sequenceTools).

Biological sex of each individual was assessed using a custom script, which estimates SNP coverage on the autosomes and X and Y chromosomes, respectively. mtDNA haplogroups were assigned using the consensus output files of schmutzi with a quality score of 30. The assignment was done by the open access software Haplogrep3 (https://haplogrep.i-med.ac.at/). Y haplogroups were assigned using the bam files with Yleaf (https://github.com/genid/Yleaf) and Yhaplo (https://github.com/23andMe/yhaplo). All assigned sexes and haplogroups can be found in table S1.

### Kinship, ROHs, and IBD

Kinship was estimated using the software KIN (*40*), which was run with standard parameters (-cnt 0, -r 1) and KIN-estimated *P* value. However, because of a different baseline of relatedness observed in Colombia_Checua_6000BP, KIN was additionally run separately on this subset of individuals, yielding an estimate with enhanced resolution. For grouped population genetic analyses, first- and second-degree relationships were excluded by removing FORM004 and PREC005, who had multiple close-kin relationships with other individuals from the same site, respectively (table S1). We estimated ROHs, as well as the states of IBD, which is only possible for male individuals in hapROH (*41*).

### Principal components analysis

To conduct PCA, we used the software smartpca (*59*) with parameter lsqproject*:* YES. To maximize SNP overlap we created three separate unmasked datasets: (i) worldwide whole genome sequences genotyped on the 1240K panel (table S8A) (*37*); (ii) Native American populations genotyped on the Illumina panel (table S8D) (*23*); and (iii) Native American populations genotyped on the Affymetrix Human Origins panel (table S8B) (*20*, *60*). We used the modern-day variation to build the PCAs and projected ancient individuals if they passed the threshold of at least 10,000 overlapping SNPs.

### Admixture analysis

We performed unsupervised admixture analyses (from *K* = 3 to *K* = 15) using the tool Admixture (version 1.3.0) with *K* = 6 as the number of clusters with lowest cross-validation error. We created a dataset comprising the masked, modern Native American Illumina dataset (table S8C) (*23*) alongside ancient Colombians, Venezuelans, and Panamanians (*20*, *44*) to infer ancestry components primarily based on present-day genetic diversity.

### Grouping and labelling

To increase resolution in population-based analyses, we grouped individuals by site considering available radiocarbon dates, archaeological information, and genetic affinity (tables S1 and S2), which shows clear intrasite homogeneity. The group labels were chosen after the format of country, site, and average calibrated radiocarbon date in yr. B.P. for each group (Fig. 1).

### *f* statistics

We performed *f* statistics with the Admixtools package (*61*) on the 1240K, Human Origins and Illumina datasets separately (table S8, A to E). One exception is the *f*_3_ outgroup statistics in fig. S20, where we intersected the Illumina panel with a dataset of present-day populations genotyped on the Human Origins dataset published by Arias *et al.* (*60*) and Capodiferro *et al.* (*20*) (table S8, B and E). For *f* statistics, we used the versions of the Illumina (*23*) and Capodiferro *et al.* (*20*) datasets where European and/or African admixtures were masked (masking protocol are described in the original publications). *f*_3_ outgroup statistics were computed using qp3Pop v.7.0.2 in the form *f*_3_(Mbuti; Pop1, Pop2), which measures the shared genetic drift between population 1 and population 2. We also created a matrix of the outgroup *f*_3_ values between a selected set of individuals/groups representing distinct South American ancestries and used it for a pairwise heat map. In addition, we converted *f*_3_ values to dissimilarities by subtracting the values from 1 and creating an MDS plot with a custom-made R script. *f*_4_ statistics were run using qpDstat v.7.0.2 with f4mode = YES. Detailed information about the construction of the *f*_4_ statistics is provided in the Supplementary Materials.

### Admixture graphs

To test the placement of Colombia_Checua_6000BP on an admixture graph, we used the Admixtools package qpGraph (v7.0.2) with parameters AllSNPs = YES and outpop = NULL on the 1240K dataset. We started with the scaffold graph reported by Posth *et al.* (*8*) that recapitulates major events during the initial peopling of the Americas. We placed Colombia_Checua_6000BP in any possible position of the admixture graph, with or without contribution to other major South Americans lineages. We then evaluated each typology individually by its *z*-score, assuming the best fitting model being the one with the lowest score, as well as observing internal nodes and branch lengths.

### TreeMix

We converted the eigenstrat files of the 1240K dataset into the input for TreeMix v.1.13 and used the genome USA_Ancient_Beringian (*2*) to root all trees. We inferred between one and five admixture edges, implementing -k 100. Tree models were plotted using TreeMix internal plotting functions and model fit after each admixture edge addition was assessed by inspection of plotted residuals and SEs.

### Radiocarbon dating

Samples for dating were sent to the Curt Engelhorn Centre for Archaeometry in Mannheim. We report direct radiocarbon dates for nine individuals, while two individuals (PREC005 and PREC001) did not have enough collagen preserved (table S1). Either a tooth root or between 500 and 1000 mg of cochlea per individual was sent for dating. Collagen was extracted using a modified Longin method and purified by ultrafiltration, retaining fractions larger than 30 kDa. The samples were additionally freeze dried after purification and then combusted to CO_2_ in an Elemental Analyzer before being catalytically converted into graphite. All samples were analyzed with accelerator mass spectrometry using a MICADAS-type machine, where samples, calibration standard (Ocalic Acid-II), blanks, and controls were measured simultaneously. All ^14^C dates were normalized to δ^13^C + −25‰ and calibration occurred using the IntCal20 (*62*) dataset in combination with the SwissCal software (L.Wacker, ETH-Zürich).

## Supplementary Materials

### The PDF file includes:

Supplementary Text Figs. S1 to S21 Legends for tables S1 to S8 References

## References

[1] M. Raghavan, M. Steinrücken, K. Harris, S. Schiffels, S. Rasmussen, M. De Giorgio, A. Albrechtsen, C. Valdiosera, M. C. Ávila-Arcos, A.-S. Malaspinas, A. Eriksson, I. Moltke, M. Metspalu, J. R. Homburger, J. Wall, O. E. Cornejo, J. V. Moreno-Mayar, T. S. Korneliussen, T. Pierre, M. Rasmussen, P. F. Campos, P. de Barros Damgaard, M. E. Allentoft, J. Lindo, E. Metspalu, R. Rodríguez-Varela, J. Mansilla, C. Henrickson, A. Seguin-Orlando, H. Malmström, T. Stafford Jr., S. S. Shringarpure, A. Moreno-Estrada, M. Karmin, K. Tambets, A. Bergström, Y. Xue, V. Warmuth, A. D. Friend, J. Singarayer, P. Valdes, F. Balloux, I. Leboreiro, J. L. Vera, H. Rangel-Villalobos, D. Pettener, D. Luiselli, L. G. Davis, E. Heyer, C. P. E. Zollikofer, M. S. Ponce de León, C. I. Smith, V. Grimes, K.-A. Pike, M. Deal, B. T. Fuller, B. Arriaza, V. Standen, M. F. Luz, F. Ricaut, N. Guidon, L. Osipova, M. I. Voevoda, O. L. Posukh, O. Balanovsky, M. Lavryashina, Y. Bogunov, E. Khusnutdinova, M. Gubina, E. Balanovska, S. Fedorova, S. Litvinov, B. Malyarchuk, M. Derenko, M. J. Mosher, D. Archer, J. Cybulski, B. Petzelt, J. Mitchell, R. Worl, P. J. Norman, P. Parham, B. M. Kemp, T. Kivisild, C. Tyler-Smith, M. S. Sandhu, M. Crawford, R. Villems, D. G. Smith, M. R. Waters, T. Goebel, J. R. Johnson, R. S. Malhi, M. Jakobsson, D. J. Meltzer, A. Manica, R. Durbin, C. D. Bustamante, Y. S. Song, R. Nielsen, E. Willerslev, Genomic evidence for the Pleistocene and recent population history of Native Americans. Science 349, aab3884 (2015). 26198033 10.1126/science.aab3884 PMC4733658

[2] J. V. Moreno-Mayar, B. A. Potter, L. Vinner, M. Steinrücken, S. Rasmussen, J. Terhorst, J. A. Kamm, A. Albrechtsen, A.-S. Malaspinas, M. Sikora, J. D. Reuther, J. D. Irish, R. S. Malhi, L. Orlando, Y. S. Song, R. Nielsen, D. J. Meltzer, E. Willerslev, Terminal Pleistocene Alaskan genome reveals first founding population of Native Americans. Nature 553, 203–207 (2018). 29323294 10.1038/nature25173

[3] M. Sikora, V. V. Pitulko, V. C. Sousa, M. E. Allentoft, L. Vinner, S. Rasmussen, A. Margaryan, P. de Barros Damgaard, C. de la Fuente, G. Renaud, M. A. Yang, Q. Fu, I. Dupanloup, K. Giampoudakis, D. Nogués-Bravo, C. Rahbek, G. Kroonen, M. Peyrot, H. McColl, S. V. Vasilyev, E. Veselovskaya, M. Gerasimova, E. Y. Pavlova, V. G. Chasnyk, P. A. Nikolskiy, A. V. Gromov, V. I. Khartanovich, V. Moiseyev, P. S. Grebenyuk, A. Y. Fedorchenko, A. I. Lebedintsev, S. B. Slobodin, B. A. Malyarchuk, R. Martiniano, M. Meldgaard, L. Arppe, J. U. Palo, T. Sundell, K. Mannermaa, M. Putkonen, V. Alexandersen, C. Primeau, N. Baimukhanov, R. S. Malhi, K.-G. Sjögren, K. Kristiansen, A. Wessman, A. Sajantila, M. M. Lahr, R. Durbin, R. Nielsen, D. J. Meltzer, L. Excoffier, E. Willerslev, The population history of northeastern Siberia since the Pleistocene. Nature 570, 182–188 (2019). 31168093 10.1038/s41586-019-1279-z PMC7617447

[4] H. Yu, M. A. Spyrou, M. Karapetian, S. Shnaider, R. Radzevičiūtė, K. Nägele, G. U. Neumann, S. Penske, J. Zech, M. Lucas, P. LeRoux, P. Roberts, G. Pavlenok, A. Buzhilova, C. Posth, C. Jeong, J. Krause, Paleolithic to Bronze Age Siberians reveal connections with first Americans and across Eurasia. Cell 181, 1232–1245.e20 (2020). 32437661 10.1016/j.cell.2020.04.037

[5] J. V. Moreno-Mayar, L. Vinner, P. de Barros Damgaard, C. de la Fuente, J. Chan, J. P. Spence, M. E. Allentoft, T. Vimala, F. Racimo, T. Pinotti, S. Rasmussen, A. Margaryan, M. Iraeta Orbegozo, D. Mylopotamitaki, M. Wooller, C. Bataille, L. Becerra-Valdivia, D. Chivall, D. Comeskey, T. Devièse, D. K. Grayson, L. George, H. Harry, V. Alexandersen, C. Primeau, J. Erlandson, C. Rodrigues-Carvalho, S. Reis, M. Q. R. Bastos, J. Cybulski, C. Vullo, F. Morello, M. Vilar, S. Wells, K. Gregersen, K. L. Hansen, N. Lynnerup, M. Mirazón Lahr, K. Kjær, A. Strauss, M. Alfonso-Durruty, A. Salas, H. Schroeder, T. Higham, R. S. Malhi, J. T. Rasic, L. Souza, F. R. Santos, A.-S. Malaspinas, M. Sikora, R. Nielsen, Y. S. Song, D. J. Meltzer, E. Willerslev, Early human dispersals within the Americas. Science 362, eaav2621 (2018). 30409807 10.1126/science.aav2621

[6] C. L. Scheib, H. Li, T. Desai, V. Link, C. Kendall, G. Dewar, P. W. Griffith, A. Mörseburg, J. R. Johnson, A. Potter, S. L. Kerr, P. Endicott, J. Lindo, M. Haber, Y. Xue, C. Tyler-Smith, M. S. Sandhu, J. G. Lorenz, T. D. Randall, Z. Faltyskova, L. Pagani, P. Danecek, T. C. O’Connell, P. Martz, A. S. Boraas, B. F. Byrd, A. Leventhal, R. Cambra, R. Williamson, L. Lesage, B. Holguin, E. Ygnacio-de Soto, J. T. Rosas, M. Metspalu, J. T. Stock, A. Manica, A. Scally, D. Wegmann, R. S. Malhi, T. Kivisild, Ancient human parallel lineages within North America contributed to a coastal expansion. Science 360, 1024–1027 (2018). 29853687 10.1126/science.aar6851

[7] M. Rasmussen, S. L. Anzick, M. R. Waters, P. Skoglund, M. DeGiorgio, T. W. Stafford Jr., S. Rasmussen, I. Moltke, A. Albrechtsen, S. M. Doyle, G. D. Poznik, V. Gudmundsdottir, R. Yadav, A. S. Malaspinas, S. S. W. V, M. E. Allentoft, O. E. Cornejo, K. Tambets, A. Eriksson, P. D. Heintzman, M. Karmin, T. S. Korneliussen, D. J. Meltzer, T. L. Pierre, J. Stenderup, L. Saag, V. M. Warmuth, M. C. Lopes, R. S. Malhi, S. Brunak, T. Sicheritz-Ponten, I. Barnes, M. Collins, L. Orlando, F. Balloux, A. Manica, R. Gupta, M. Metspalu, C. D. Bustamante, M. Jakobsson, R. Nielsen, E. Willerslev, The genome of a Late Pleistocene human from a Clovis burial site in western Montana. Nature 506, 225–229 (2014). 24522598 10.1038/nature13025 PMC4878442

[8] C. Posth, N. Nakatsuka, I. Lazaridis, P. Skoglund, S. Mallick, T. C. Lamnidis, N. Rohland, K. Nägele, N. Adamski, E. Bertolini, N. Broomandkhoshbacht, A. Cooper, B. J. Culleton, T. Ferraz, M. Ferry, A. Furtwängler, W. Haak, K. Harkins, T. K. Harper, T. Hünemeier, A. M. Lawson, B. Llamas, M. Michel, E. Nelson, J. Oppenheimer, N. Patterson, S. Schiffels, J. Sedig, K. Stewardson, S. Talamo, C.-C. Wang, J.-J. Hublin, M. Hubbe, K. Harvati, A. N. Delaunay, J. Beier, M. Francken, P. Kaulicke, H. Reyes-Centeno, K. Rademaker, W. R. Trask, M. Robinson, S. M. Gutierrez, K. M. Prufer, D. C. Salazar-García, E. N. Chim, L. M. P. Gomes, M. L. Alves, A. Liryo, M. Inglez, R. E. Oliveira, D. V. Bernardo, A. Barioni, V. Wesolowski, N. A. Scheifler, M. A. Rivera, C. R. Plens, P. G. Messineo, L. Figuti, D. Corach, C. Scabuzzo, S. Eggers, P. De Blasis, M. Reindel, C. Méndez, G. Politis, E. Tomasto-Cagigao, D. J. Kennett, A. Strauss, L. Fehren-Schmitz, J. Krause, D. Reich, Reconstructing the deep population history of Central and South America. Cell 175, 1185–1197.e22 (2018). 30415837 10.1016/j.cell.2018.10.027 PMC6327247

[9] N. Nakatsuka, B. Holguin, J. Sedig, P. E. Langenwalter, J. Carpenter, B. J. Culleton, C. García-Moreno, T. K. Harper, D. Martin, J. Martínez-Ramírez, A. Porcayo-Michelini, V. Tiesler, M. E. Villapando-Canchola, A. Valdes Herrera, K. Callan, E. Curtis, A. Kearns, L. Iliev, A. M. Lawson, M. Mah, S. Mallick, A. Micco, M. Michel, J. N. Workman, J. Oppenheimer, L. Qiu, F. Zalzala, N. Rohland, J. L. Punzo Diaz, J. R. Johnson, D. Reich, Genetic continuity and change among the Indigenous peoples of California. Nature 624, 122–129 (2023). 37993721 10.1038/s41586-023-06771-5 PMC10872549

[10] T. Ferraz, X. S. Villagran, K. Nägele, R. Radzevičiūtė, R. B. Lemes, D. C. Salazar-García, V. Wesolowski, M. L. Alves, M. Bastos, A. R. Py-Daniel, H. P. Lima, J. M. Cardoso, R. Estevam, A. Liryo, G. M. Guimarães, L. Figuti, S. Eggers, C. R. Plens, D. M. A. Erler, H. A. V. Costa, I. da Silva Erler, E. Koole, G. Henriques, A. Solari, G. Martin, S. F. S. M. da Silva, R. Kipnis, L. M. Müller, M. Ferreira, J. C. Resende, E. Chim, C. A. da Silva, A. C. Borella, T. Tomé, L. M. P. Gomes, D. B. Fonseca, C. S. da Rosa, J. D. de Moura Saldanha, L. C. Leite, C. M. S. Cunha, S. A. Viana, F. O. Almeida, D. Klokler, H. L. A. Fernandes, S. Talamo, P. De Blasis, S. M. de Souza, C. de Paula Moraes, R. E. Oliveira, T. Hünemeier, A. Strauss, C. Posth, Genomic history of coastal societies from eastern South America. Nat. Ecol. Evol. 7, 1315–1330 (2023). 37524799 10.1038/s41559-023-02114-9 PMC10406606

[11] N. Nakatsuka, I. Lazaridis, C. Barbieri, P. Skoglund, N. Rohland, S. Mallick, C. Posth, K. Harkins-Kinkaid, M. Ferry, É. Harney, M. Michel, K. Stewardson, J. Novak-Forst, J. M. Capriles, M. A. Durruty, K. A. Álvarez, D. Beresford-Jones, R. Burger, L. Cadwallader, R. Fujita, J. Isla, G. Lau, C. L. Aguirre, S. LeBlanc, S. C. Maldonado, F. Meddens, P. G. Messineo, B. J. Culleton, T. K. Harper, J. Quilter, G. Politis, K. Rademaker, M. Reindel, M. Rivera, L. Salazar, J. R. Sandoval, C. M. Santoro, N. Scheifler, V. Standen, M. I. Barreto, I. F. Espinoza, E. Tomasto-Cagigao, G. Valverde, D. J. Kennett, A. Cooper, J. Krause, W. Haak, B. Llamas, D. Reich, L. Fehren-Schmitz, A paleogenomic reconstruction of the deep population history of the Andes. Cell 181, 1131–1145.e21 (2020). 32386546 10.1016/j.cell.2020.04.015 PMC7304944

[12] J. W. Hoopes, O. Fonseca, “Goldwork and Chibchan identity: Endogenous change and diffuse unity in the Isthmo-Colombian area,” in *Gold and Power in Ancient Costa Rica, Panama, and Colombia* (Dumbarton Oaks, 2003), pp. 49–89.

[13] J. C. Niño Vargas, S. Beckerman, *“*Universo chibcha, universos chibchas: introducción a la unidad y la diversidad del área istmocolombiana,” in *Universos chibchas: Nuevas aproximaciones a la unidad y la diversidad humana del área istmocolombiana.* J. C. Nino Vargas, S. Beckerman eds. (Universidad de los Andes, 2024), pp. 1–59.

[14] M. Pache, “Contribution to Chibchan historical linguistics,” thesis, University Leiden (2018).

[15] M. Pache, Tracing sound change in Nasa Yuwe (western Colombia): Evidence from Andaqui (western Colombia) and Misumalpan languages (Central America). LIAMES: Línguas Indígenas Americanas 24, e024003 (2024).

[16] M. Urban, Language classification, language contact and Andean prehistory: The North. Lang. Linguist. Compass 15, e12414 (2021).

[17] A. Constenla Umaña, L. Campbell, V. Grondona, “Chibchan languages,” in *The Indigenous Languages of South America: A Comprehensive Guide,* V. G. L. Campbell, Ed. (De Gruyter Mouton, 2012), pp. 391–439.

[18] A. Constenla Umaña, Sobre el estudio diacrónico de las lenguas chibchenses y su contribución al conocimiento del pasado de sus hablantes. Boletín Museo del Oro 3839, 1356 (1995).

[19] L. Casas Vargas, L. M. Romero, W. Usaquén, S. Zea, M. Silva, I. Briceño, A. Gomez, J. V. Rodríguez, Mitochondrial DNA diversity in Prehispanic bone remains on the Eastern Colombian Andes. Biomedica 37, 548–560 (2017). 29373774 10.7705/biomedica.v37i4.3377

[20] M. R. Capodiferro, B. Aram, A. Raveane, N. Rambaldi Migliore, G. Colombo, L. Ongaro, J. Rivera, T. Mendizábal, I. Hernández-Mora, M. Tribaldos, U. A. Perego, H. Li, C. L. Scheib, A. Modi, A. Gòmez-Carballa, V. Grugni, G. Lombardo, G. Hellenthal, J. M. Pascale, F. Bertolini, G. S. Grieco, C. Cereda, M. Lari, D. Caramelli, L. Pagani, M. Metspalu, R. Friedrich, C. Knipper, A. Olivieri, A. Salas, R. Cooke, F. Montinaro, J. Motta, A. Torroni, J. G. Martín, O. Semino, R. S. Malhi, A. Achilli, Archaeogenomic distinctiveness of the Isthmo-Colombian area. Cell 184, 1706–1723.e24 (2021). 33761327 10.1016/j.cell.2021.02.040 PMC8024902

[21] P. E. Melton, I. Briceño, A. Gómez, E. J. Devor, J. E. Bernal, M. H. Crawford, Biological relationship between central and South American Chibchan speaking populations: Evidence from mtDNA. Am. J. Phys. Anthropol. 133, 753–770 (2007). 17340631 10.1002/ajpa.20581

[22] M. C. Noguera-Santamaría, C. E. Anderson, D. Uricoechea, C. Durán, I. Briceño-Balcázar, J. Bernal Villegas, Mitochondrial DNA analysis suggests a Chibchan migration into Colombia. Universitas Scientiarum 20, 261–278 (2015).

[23] D. Reich, N. Patterson, D. Campbell, A. Tandon, S. Mazieres, N. Ray, M. V. Parra, W. Rojas, C. Duque, N. Mesa, L. F. García, O. Triana, S. Blair, A. Maestre, J. C. Dib, C. M. Bravi, G. Bailliet, D. Corach, T. Hünemeier, M. C. Bortolini, F. M. Salzano, M. L. Petzl-Erler, V. Acuña-Alonzo, C. Aguilar-Salinas, S. Canizales-Quinteros, T. Tusié-Luna, L. Riba, M. Rodríguez-Cruz, M. Lopez-Alarcón, R. Coral-Vazquez, T. Canto-Cetina, I. Silva-Zolezzi, J. C. Fernandez-Lopez, A. V. Contreras, G. Jimenez-Sanchez, M. J. Gómez-Vázquez, J. Molina, A. Carracedo, A. Salas, C. Gallo, G. Poletti, D. B. Witonsky, G. Alkorta-Aranburu, R. I. Sukernik, L. Osipova, S. A. Fedorova, R. Vasquez, M. Villena, C. Moreau, R. Barrantes, D. Pauls, L. Excoffier, G. Bedoya, F. Rothhammer, J.-M. Dugoujon, G. Larrouy, W. Klitz, D. Labuda, J. Kidd, K. Kidd, A. D. Rienzo, N. B. Freimer, A. L. Price, A. Ruiz-Linares, Reconstructing Native American population history. Nature 488, 370–374 (2012). 22801491 10.1038/nature11258 PMC3615710

[24] A. Moreno-Estrada, S. Gravel, F. Zakharia, J. L. McCauley, J. K. Byrnes, C. R. Gignoux, P. A. Ortiz-Tello, R. J. Martínez, D. J. Hedges, R. W. Morris, C. Eng, K. Sandoval, S. Acevedo-Acevedo, P. J. Norman, Z. Layrisse, P. Parham, J. C. Martínez-Cruzado, E. G. Burchard, M. L. Cuccaro, E. R. Martin, C. D. Bustamante, Reconstructing the population genetic history of the Caribbean. PLOS Genet. 9, e1003925 (2013). 24244192 10.1371/journal.pgen.1003925 PMC3828151

[25] G. Correal Urrego, *Aguazuque*. *Evidencia de cazadores, recolectores y plantadores en la altiplanicie de la cordillera oriental* (FIAN, 1990).

[26] A. M. Groot, *Checua: Una secuencia cultural entre 8 500 y 3 000 años antes del presente* (FIAN, 1992).

[27] J. V. Rodríguez, *Tras las huellas de los chibchas de los Andes Orientales de Colombia* (ICANH, 2024).

[28] A. Gómez, J. C. Berrio, H. Henry, M. Becerra, R. Marchant, A Holocene pollen record of vegetation change and human impact from Pantano de Vargas, an intra-Andean basin of Duitama, Colombia. Rev. Palaeobot. Palynol. 145, 143–157 (2007).

[29] S. M. Broadbent, Reconocimiento arqueológico de la laguna de La Herrera. Revista colombiana de antropología 15, 173–191 (1970).

[30] M. E. Delgado Burbano, Mid and Late Holocene population changes at the Sabana de Bogotá (Northern South America) inferred from skeletal morphology and radiocarbon chronology. Quat. Int. 256, 2–11 (2012).

[31] M. Delgado, F. Rodríguez, K. Kassadjikova, L. Fehren-Schmitz, A paleogenetic perspective of the Sabana de Bogotá (Northern South America) population history over the Holocene (9000–550 cal BP). Quat. Int. 578, 73–86 (2021).

[32] A. M. Boada Rivas, *The Evolution of Social Hierarchy in a Muisca Chiefdom of the Northern Andes of Colombia* (University of Pittsburgh,Universidad de los Andes, 2007).

[33] S. Archila, A. M. Groot, J. P. Ospina, M. Mejía, C. Zorro, Dwelling the hill: Traces of increasing sedentism in hunter-gatherers societies at Checua site, Colombia (9500-5052 cal BP). Quat. Int. 578, 102–119 (2021).

[34] C. H. Langebaek, *Regional Archaeology in the Muisca territory: A Study of the Fúquene and Susa Valleys* (University of Pittsburgh, Universidad de los Andes, 1995).

[35] A. Casas-Vargas, A. Gómez, I. Briceño, M. Díaz-Matallana, J. E. Bernal, J. V. Rodríguez, High genetic diversity on a sample of pre-Columbian bone remains from Guane territories in northwestern Colombia. Am. J. Phys. Anthropol. 146, 637–649 (2011). 21990065 10.1002/ajpa.21626

[36] M. Díaz-Matallana, A. Gómez, I. Briceño, J. V. Rodríguez, Genetic analysis of Paleo-Colombians from Nemocón, Cundinamarca provides insights on the early peopling of northwestern South America. Revista Acad. Colomb. Ci. Exact. 40, 461–483 (2016).

[37] S. Mallick, H. Li, M. Lipson, I. Mathieson, M. Gymrek, F. Racimo, M. Zhao, N. Chennagiri, S. Nordenfelt, A. Tandon, P. Skoglund, I. Lazaridis, S. Sankararaman, Q. Fu, N. Rohland, G. Renaud, Y. Erlich, T. Willems, C. Gallo, J. P. Spence, Y. S. Song, G. Poletti, F. Balloux, G. van Driem, P. de Knijff, I. G. Romero, A. R. Jha, D. M. Behar, C. M. Bravi, C. Capelli, T. Hervig, A. Moreno-Estrada, O. L. Posukh, E. Balanovska, O. Balanovsky, S. Karachanak-Yankova, H. Sahakyan, D. Toncheva, L. Yepiskoposyan, C. Tyler-Smith, Y. Xue, M. S. Abdullah, A. Ruiz-Linares, C. M. Beall, A. di Rienzo, C. Jeong, E. B. Starikovskaya, E. Metspalu, J. Parik, R. Villems, B. M. Henn, U. Hodoglugil, R. Mahley, A. Sajantila, G. Stamatoyannopoulos, J. T. S. Wee, R. Khusainova, E. Khusnutdinova, S. Litvinov, G. Ayodo, D. Comas, M. F. Hammer, T. Kivisild, W. Klitz, C. A. Winkler, D. Labuda, M. Bamshad, L. B. Jorde, S. A. Tishkoff, W. S. Watkins, M. Metspalu, S. Dryomov, R. Sukernik, L. Singh, K. Thangaraj, S. Pääbo, J. Kelso, N. Patterson, D. Reich, The Simons genome diversity project: 300 genomes from 142 diverse populations. Nature 538, 201–206 (2016). 27654912 10.1038/nature18964 PMC5161557

[38] Y.-Z. Huang, H. Pamjav, P. Flegontov, V. Stenzl, S.-Q. Wen, X.-Z. Tong, C.-C. Wang, L.-X. Wang, L.-H. Wei, J.-Y. Gao, L. Jin, H. Li, Dispersals of the Siberian Y-chromosome haplogroup Q in Eurasia. Mol. Genet. Genomics 293, 107–117 (2018). 28884289 10.1007/s00438-017-1363-8 PMC5846874

[39] V. Grugni, A. Raveane, L. Ongaro, V. Battaglia, B. Trombetta, G. Colombo, M. R. Capodiferro, A. Olivieri, A. Achilli, U. A. Perego, J. Motta, M. Tribaldos, S. R. Woodward, L. Ferretti, F. Cruciani, A. Torroni, O. Semino, Analysis of the human Y-chromosome haplogroup Q characterizes ancient population movements in Eurasia and the Americas. BMC Biol. 17, 3 (2019). 30674303 10.1186/s12915-018-0622-4 PMC6345020

[40] D. Popli, S. Peyrégne, B. M. Peter, KIN: A method to infer relatedness from low-coverage ancient DNA. Genome Biol. 24, 10 (2023). 36650598 10.1186/s13059-023-02847-7 PMC9843908

[41] H. Ringbauer, J. Novembre, M. Steinrücken, Parental relatedness through time revealed by runs of homozygosity in ancient DNA. Nat. Commun. 12, 5425 (2021). 34521843 10.1038/s41467-021-25289-w PMC8440622

[42] J. L. Garcia, “The foods and crops of the Muisca: A dietary reconstruction of the intermediate chiefdoms of Bogotá (Bacatá) and Tunja (Hunza), Colombia,” thesis, University of Central Florida (2012).

[43] F. M. Olivares, J. M. Madero, A. Casas-Vargas, S. Z. Montoya, D. S. Medellín, L. Gusmão, W. Usaquén, Contrasting the ancestry patterns of three distinct population groups from the northernmost region of South America. Am. J. Phys. Anthropol. 173, 437–447 (2020). 32856314 10.1002/ajpa.24130

[44] D. M. Fernandes, K. A. Sirak, H. Ringbauer, J. Sedig, N. Rohland, O. Cheronet, M. Mah, S. Mallick, I. Olalde, B. J. Culleton, N. Adamski, R. Bernardos, G. Bravo, N. Broomandkhoshbacht, K. Callan, F. Candilio, L. Demetz, K. S. D. Carlson, L. Eccles, S. Freilich, R. J. George, A. M. Lawson, K. Mandl, F. Marzaioli, W. C. McCool, J. Oppenheimer, K. T. Özdogan, C. Schattke, R. Schmidt, K. Stewardson, F. Terrasi, F. Zalzala, C. A. Antúnez, E. V. Canosa, R. Colten, A. Cucina, F. Genchi, C. Kraan, F. La Pastina, M. Lucci, M. V. Maggiolo, B. Marcheco-Teruel, C. T. Maria, C. Martínez, I. París, M. Pateman, T. M. Simms, C. G. Sivoli, M. Vilar, D. J. Kennett, W. F. Keegan, A. Coppa, M. Lipson, R. Pinhasi, D. Reich, A genetic history of the pre-contact Caribbean. Nature 590, 103–110 (2021). 33361817 10.1038/s41586-020-03053-2 PMC7864882

[45] K. Nägele, C. Posth, M. Iraeta Orbegozo, Y. Chinique de Armas, S. T. Hernández Godoy, U. M. González Herrera, M. A. Nieves-Colón, M. Sandoval-Velasco, D. Mylopotamitaki, R. Radzeviciute, J. Laffoon, W. J. Pestle, J. Ramos-Madrigal, T. C. Lamnidis, W. C. Schaffer, R. S. Carr, J. S. Day, C. Arredondo Antúnez, A. Rangel Rivero, A. J. Martínez-Fuentes, E. Crespo-Torres, I. Roksandic, A. C. Stone, C. Lalueza-Fox, M. Hoogland, M. Roksandic, C. L. Hofman, J. Krause, H. Schroeder, Genomic insights into the early peopling of the Caribbean. Science 369, 456–460 (2020). 32499399 10.1126/science.aba8697

[46] J. W. Hoopes, The emergence of social complexity in the Chibchan world of southern Central America and northern Colombia, AD 300–600. J. Archaeol. Res. 13, 1–47 (2005).

[47] J. V. Rodríguez, C. Vargas Vargas, Evolución y tamaño dental en poblaciones humanas de Colombia. Revista Acad. Colomb. Ci. Exact. 34, 423–439 (2010).

[48] M. Delgado, Stable isotope evidence for dietary and cultural change over the Holocene at the Sabana de Bogotá region, Northern South America. Archaeol. Anthropol. Sci. 10, 817–832 (2018).

[49] D. J. Kennett, M. Lipson, K. M. Prufer, D. Mora-Marín, R. J. George, N. Rohland, M. Robinson, W. R. Trask, H. H. J. Edgar, E. C. Hill, E. E. Ray, P. Lynch, E. Moes, L. O’Donnell, T. K. Harper, E. J. Kate, J. Ramos, J. Morris, S. M. Gutierrez, T. M. Ryan, B. J. Culleton, J. J. Awe, D. Reich, South-to-north migration preceded the advent of intensive farming in the Maya region. Nat. Commun. 13, 1530 (2022). 35318319 10.1038/s41467-022-29158-y PMC8940966

[50] J. Dabney, M. Knapp, I. Glocke, M.-T. Gansauge, A. Weihmann, B. Nickel, C. Valdiosera, N. García, S. Pääbo, J.-L. Arsuaga, M. Meyer, Complete mitochondrial genome sequence of a Middle Pleistocene cave bear reconstructed from ultrashort DNA fragments. Proc. Natl. Acad. Sci. U.S.A. 110, 15758–15763 (2013). 24019490 10.1073/pnas.1314445110 PMC3785785

[51] N. Rohland, E. Harney, S. Mallick, S. Nordenfelt, D. Reich, Partial uracil–DNA–glycosylase treatment for screening of ancient DNA. Philos. Trans. R Soc. Lond. B Biol. Sci. 370, 20130624 (2015). 25487342 10.1098/rstb.2013.0624 PMC4275898

[52] A. Peltzer, G. Jäger, A. Herbig, A. Seitz, C. Kniep, J. Krause, K. Nieselt, EAGER: Efficient ancient genome reconstruction. Genome Biol. 17, 60 (2016). 27036623 10.1186/s13059-016-0918-z PMC4815194

[53] M. Schubert, S. Lindgreen, L. Orlando, AdapterRemoval v2: Rapid adapter trimming, identification, and read merging. BMC. Res. Notes 9, 88 (2016). 26868221 10.1186/s13104-016-1900-2 PMC4751634

[54] H. Li, R. Durbin, Fast and accurate short read alignment with Burrows-Wheeler transform. Bioinformatics 25, 1754–1760 (2009). 19451168 10.1093/bioinformatics/btp324 PMC2705234

[55] H. Jónsson, A. Ginolhac, M. Schubert, P. L. F. Johnson, L. Orlando, mapDamage2.0: Fast approximate Bayesian estimates of ancient DNA damage parameters. Bioinformatics 29, 1682–1684 (2013). 23613487 10.1093/bioinformatics/btt193 PMC3694634

[56] Q. Fu, M. Meyer, X. Gao, U. Stenzel, H. A. Burbano, J. Kelso, S. Pääbo, DNA analysis of an early modern human from Tianyuan Cave, China. Proc. Natl. Acad. Sci. U.S.A. 110, 2223–2227 (2013). 23341637 10.1073/pnas.1221359110 PMC3568306

[57] G. Renaud, V. Slon, A. T. Duggan, J. Kelso, Schmutzi: Estimation of contamination and endogenous mitochondrial consensus calling for ancient DNA. Genome Biol. 16, 224 (2015). 26458810 10.1186/s13059-015-0776-0 PMC4601135

[58] T. S. Korneliussen, A. Albrechtsen, R. Nielsen, ANGSD: Analysis of next generation sequencing data. BMC Bioinformatics 15, 356 (2014). 25420514 10.1186/s12859-014-0356-4 PMC4248462

[59] N. Patterson, A. L. Price, D. Reich, Population structure and eigenanalysis. PLOS Genet. 2, e190 (2006). 17194218 10.1371/journal.pgen.0020190 PMC1713260

[60] L. Arias, N. Q. Emlen, S. Norder, N. Julmi, M. Lemus Serrano, T. Chacon, J. Wiegertjes, A. Howard, M. C. B. C. Azevedo, A. Caine, S. Dunn, M. Stoneking, R. Van Gijn, Interpreting mismatches between linguistic and genetic patterns among speakers of Tanimuka (Eastern Tukanoan) and Yukuna (Arawakan). Interface Focus 13, 20220056 (2023). 36655193 10.1098/rsfs.2022.0056 PMC9732642

[61] N. Patterson, P. Moorjani, Y. Luo, S. Mallick, N. Rohland, Y. Zhan, T. Genschoreck, T. Webster, D. Reich, Ancient admixture in human history. Genetics 192, 1065–1093 (2012). 22960212 10.1534/genetics.112.145037 PMC3522152

[62] M. Stuiver, H. A. Polach, Discussion reporting of ^14^C data. Radiocarbon 19, 355–363 (1977).

[63] S. I. Perez, V. Bernal, P. N. Gonzalez, M. Sardi, G. G. Politis, Discrepancy between cranial and DNA data of early Americans: Implications for American peopling. PLOS ONE 4, e5746 (2009). 19478947 10.1371/journal.pone.0005746 PMC2684646

[64] M. Hubbe, W. A. Neves, K. Harvati, Testing evolutionary and dispersion scenarios for the settlement of the new world. PLOS ONE 5, e11105 (2010). 20559441 10.1371/journal.pone.0011105 PMC2885431

[65] S. de Azevedo, A. Nocera, C. Paschetta, L. Castillo, M. González, R. González-José, Evaluating microevolutionary models for the early settlement of the New World: The importance of recurrent gene flow with Asia. Am. J. Phys. Anthropol. 146, 539–552 (2011). 21805463 10.1002/ajpa.21564

[66] R. González-José, M. C. Bortolini, F. R. Santos, S. L. Bonatto, The peopling of America: Craniofacial shape variation on a continental scale and its interpretation from an interdisciplinary view. Am. J. Phys. Anthropol. 137, 175–187 (2008). 18481303 10.1002/ajpa.20854

[67] G. Keyeux, C. Rodas, N. Gelvez, D. Carter, Possible migration routes into South America deduced from mitochondrial DNA studies in Colombian Amerindian populations. Hum. Biol. 74, 211–233 (2002). 12030650 10.1353/hub.2002.0022

[68] J. V. Rodríguez Cuenca, *La identificación humana en Colombia. Avances y perspectivas* (Universidad Nacional de Colombia, 2011).

[69] J. V. Rodríguez Cuenca, *El Parque Arqueológico de Facatativá* (CAR, Universidad Nacional, 2015).

[70] C. H. Langebaek, in *Mercados, poblamiento e integración étnica entre los muiscas* (Banco de la República, 1986).

[71] R. Lleras, Los Muiscas en la literatura histórica y antropológica. Bol. Hist. Antig. 92, 307–338 (2005).

[72] A. M. Groot, Arqueología y patrimonio: Conocimiento y apropiación social. Revista Acad. Colomb. Ci. Exact. 10.18257/raccefyn.30(114).2006.2210, (2006).

[73] A. M. Groot, “Checua: Un aporte para el conocimiento del precerámico de la sabana de Bogotá,” in *Ámbito y Ocupaciones Tempranas de la América Tropical* (Instituto Colombiano de Antropología, 1995), pp. 45–58.

[74] A. Minelli, M. Cozzolino, A. Di Nucci, S. Guglielmi, M. Giannantonio, D. D'Amore, E. Pittoni, A. M. Groot, The prehistory of the Colombian territory: The result of the Italian archaeological investigation on the Checua Site (Municipality of Nemocòn, Cundinamarca Department). J. Biol. Res.-Boll. Soc. Ital. Biol. Sper. 85, 10.4081/jbr.2012.4073 (2012).

[75] W. A. Neves, M. Hubbe, G. Correal, Human skeletal remains from Sabana de Bogotá, Colombia: A case of Paleoamerican morphology late survival in South America? Am. J. Phys. Anthropol. 133, 1080–1098 (2007). 17554759 10.1002/ajpa.20637

[76] H. M. Pucciarelli, S. I. Perez, G. G. Politis, Early Holocene human remains from the Argentinean Pampas: Additional evidence for distinctive cranial morphology of early South Americans. Am. J. Phys. Anthropol. 143, 298–305 (2010). 20623674 10.1002/ajpa.21347

[77] E. E. R. Braida, Arqueología de rescate, en el barrio las delicias (Bogotá). Rev. Colomb. Antropol. 28, 156–160 (1991).

[78] A. Cifuentes Toro, Reseña de un sitio arqueológico en la Mesa de los Santos (Santander). Bol. Arqueol. FIAN 4, 33–40 (2014).

[79] S. Mallick, A. Micco, M. Mah, H. Ringbauer, I. Lazaridis, I. Olalde, N. Patterson, D. Reich, The Allen Ancient DNA Resource (AADR) a curated compendium of ancient human genomes. Sci. Data 11, 182 (2024). 38341426 10.1038/s41597-024-03031-7 PMC10858950

[80] M. H. Kruschek, “The evolution of the Bogotá chiefdom: A household view,” thesis, University of Pittsburgh (2003).

[81] S. Rivas, D. Calderón, C. Marulanda, L. F. Mendoza, G. R. Scott, S. R. Poulson, M. Delgado, Stable isotopes and paleodiet of the ancient inhabitants of Nueva Esperanza: A late Holocene site from Sabana de Bogotá (Colombia). Int. J. Osteoarchaeol. 34, e3244 (2024).

[82] J. P. Quintero-Guzmán, El Dorado offerings in Lake Guatavita: A muisca ritual archaeological site. Latin Am. Antiq. 35, 483–499 (2024).

[83] P. A. Sánchez-Castañeda, Memory in sacred places: The revitalization process of the Muisca community. Urban Plan. 5, 263–273 (2020).

[84] J. V. Rodríguez, *Los chibchas: Hijos del sol, la luna y los Andes. Orígenes de su diversidad* (Universidad Nacional de Colombia, 2011).

