# Supplementary material for: A 6000-year-long genomic transect from the Bogotá Altiplano reveals multiple genetic shifts in the demographic history of Colombia
Source: Sci Adv. 2025 May 28;11(22):eads6284. doi: 10.1126/sciadv.ads6284 (PMC12118548; doi:10.1126/sciadv.ads6284)
Supplement: Supplementary file 1 — Supplementary Text Figs. S1 to S21 Legends for tables S1 to S8 References [file sciadv.ads6284_sm.pdf]

Science Advances
AAAS

Supplementary Materials for

A 6000-year-long genomic transect from the Bogotá Altiplano reveals multiple genetic shifts in the demographic history of Colombia

Kim-Louise Krettek *et al.*

Corresponding author: Kim-Louise Krettek, kim-louise.krettek@uni-tuebingen.de;
Andrea Casas-Vargas, lacasasv@unal.edu.co; Cosimo Posth, cosimo.posth@uni-tuebingen.de

Sci. Adv. **11**, eads6284 (2025)
DOI: 10.1126/sciadv.ads6284

The PDF file includes:

Supplementary Text
Figs. S1 to S21
Legends for tables S1 to S8
References

Other Supplementary Material for this manuscript includes the following:

Tables S1 to S8

Supplementary Text

Anthropological and archaeological context

The reconstruction of the peopling of South America has raised different hypotheses on the origin of the first settlers. Two of the main theories for interpreting biological variation in Native American populations, which have exhibited heterogeneous traits in their cranial and dental morphology, are based on archeological and anthropological evidence. The oldest skeletons differ morphologically from those of late Native Americans. The first, so-called "Paleoamericans", are characterized by having longer, narrower and smaller skulls (Dolichocephalic) with larger teeth (Sundadont), while later populations, so-called "Amerindians", tend to have a more rounded skull and a wider cranial vault (Brachycephalic) with smaller teeth (Sinodont) (fig. S1-S5) (63). These characteristics have led to different hypotheses about the possible origin of these settlers: 1) Migratory hypothesis, which suggests that variation among South American groups was the result of multiple waves of migration and 2) Microevolutionary hypothesis, which explains the emergence of biological diversity as consequence of evolutionary processes (e.g. genetic drift, natural selection), so that all Indigenous groups would be descendants of the same ancestral population (*64, 65, 66*).
Given its geographical location, Colombia was a mandatory steppingstone for entering (and leaving) South America. Several studies have been carried out from both genetic and archeological perspectives, to elucidate possible routes into Colombia and the subcontinent, with no definite conclusions (*21, 67*).
According to archaeological data, one region that has shed light on the first human settlements of Colombia is the Eastern Andes, since this region allows to test several hypotheses regarding their origins. The pre-colonial phase is commonly divided into three periods in which human populations occupied this mountain range: Preceramic (~12,000 to 3,000 BP), Formative (~3,000 BP to 1,000 BP) and Muisca (~1,000 BP to 400 BP) (*68, 69*). Populations from these periods possibly had different origins and were the product of several migration waves, displacing or integrating predecessor groups (*70, 71*). Bio-anthropological studies have suggested that the Muisca settlements can be traced back to the Formative period without major migratory processes of foreign peoples, but rather through microevolutionary processes (*68*).

Description of analyzed individuals and archeological sites

In the current study we analyzed a total of 21 ancient individuals from five sites associated with the Preceramic period (Checua), Formative period (Laguna de La Herrera) and Muisca period (Las Delicias, Soacha, Purnia). A description of the archeological sites and analyzed specimens is provided below and in table S1.

Checua (PREC), Nemocon, Bogotá ~6,000 BP
Coordinates: 5.0674157 Latitude, -73.8849739 Longitude

- PREC001 (93-CHII-03): no collagen preserved, MAMS-54219
- PREC002 (CHII-08): 3,951-3,789 calBC, MAMS-54221
- PREC003 (CHII-06)

- PREC004 (CHII-07): 4,699-4,545 calBC, MAMS-5418
- PREC005 (CHII-04): no collagen preserved, MAMS-52227
- PREC006 (CHII02A)
- PREC007 (CHII-03A): 3,951-3,788 calBC, MAMS-54220

The Checua site in Nemocón, Bogotá is an open-air site extending over the top of a hill, rising about 15 feet above the surrounding area. It was named after the subdivision of the municipality containing the site, a region of subtropical lower-montane dry forest with a 2,600 m.a.s.l. average altitude, harboring the Checua river and the Neusa river (*26, 72-74*). The site has produced archeological evidence for hunter-gatherer presence between 8,500 and 3,000 BP (radiocarbon dated) with two extensive excavation campaigns subdividing the site into eight (first excavation) and seven (more recent excavation) stratigraphic layers (*26, 72-74*). The first excavation, covering 300 m^2, conducted by A.M. Groot and colleagues in 1991, revealed eight stratigraphic levels with two human occupation zones, with zone one being dated to 9,500-8,700 BP and zone two to 9,100-8,300 BP (*26, 75*). Human remains found at the site were identified as Indigenous people based on anthropological assessment (*26, 76*). Lithic tools, faunal remains and bone fragments linked the site to hunter-gatherer activities (*26, 72-74*), painting a picture of complex funerary processes and cultural practices.

Laguna de La Herrera (FORM), Madrid, Bogotá ~2,000 BP
Coordinates: 4.7333069 Latitude, -74.2827857 Longitude

- FORM001 (Madrid 2-41 individuo 8)
- FORM002 (Madrid Ind 1): 36 cal BC-117 cal AD, MAMS-52224
- FORM003 (Madrid Ind 5)
- FORM004 (Madrid 2-41)
- FORM005 (Madrid 2-41 Indv 6)
- FORM006 (Madrid 2-41 Indv 2)
- FORM007 (Madrid 2-41 Indv 14)
- FORM008 (Madrid 2-41 Indv 15 corte 0)
- FORM009 (Madrid 2-41 corte 8 cráneo infantile)

Madrid in Cundinamarca is a collective group of burials belonging to the Early Formative period (3,000 to 2,000 BP) and located on the edge of the ancient Laguna de La Herrera. The remains were accompanied by foreign pottery from the Magdalena River valley, animal bones and ochre. Their physical features display a Paleoamerican morphology, and some individuals showed osteological signs that they suffered from infections of treponematosis origin. A remarkable feature of the site is an astronomical observatory, which was built during the Late Formative period (2,000 BP to 1,000 BP), consisting of inverted pyramids dug into the ground, cones and canals aligned 24˚ NW.

Las Delicias (EMUI), Bogotá ~1,200BP
Coordinates: 4.6486259 Latitude, -74.2478931 Longitude

- EMUI001 (Delicias Marzo 14-90): 664-773 cal BC, MAMS-52223
- EMUI002 (Delicias RS-1)

The site Las Delicias, within the neighborhood of Las Delicias in Bogotá, was discovered during construction work. The site has been severely destroyed by looting as well as environmental processes. Initial assessment of suitability for becoming an archeological study site was conducted by the Colombian Institute of Anthropology in collaboration with Dr. Francisco Guacheta. The site comprises a residential area and a burial area located on an alluvial terrace of the Tunjuelito River and was inhabited between 1010 ± 60 BP and 1180 ± 70 BP according to radiocarbon dates obtained from charcoal as well as archeological material found at the site. Within the site, houses containing artifacts as well as occasional burials without funerary goods were found. Based on archeological evidence, the inhabitants consumed flora and fauna that is currently extinct in the area, as well as maize. Furthermore, the archeological material was associated with the Muisca culture (*77*). During an excavation conducted by Enciso, the remains of 19 human individuals were found within the boundaries of the burial area.

Soacha (MUIS), Soacha, Bogotá ~520 BP
Coordinates: 4.5754900 Latitude, -74.2312017 Longitude

- MUIS001 (Soacha 1987 T50): 1,413-1,447 calAD, MAMS-52226

At the Portalegre site of Soacha, Cundinamarca, Álvaro Botiva (1988) excavated a total of 130 tombs and four floors of houses dated to 900 to 700 BP. Most of the tombs are simple rectangular shallow pits of which 10 % were covered with slabs. The bodies were in the extended dorsal decubitus position, predominantly facing south and east. The grave goods consisted of moccasins, bowls, cups, jars, two-handled globular pots, seashell beads and some lithic artifacts (spindle whorls, grinding hands, metates and an axe). Shuttle hooks and bone needles seem to be associated with male individuals, while spindle whorls seem to be associated with female individuals.

Purnia (GUAN), Mesa de los Santos, Santander ~530 BP
Coordinates: 6.9425442 Latitude, -73.0445416 Longitude

- GUAN001 (Purnia 19): 1,402-1,436 calAD, MAMS-52225
- GUAN002 (Purnia 01)

Purnia refers to an archeological site located within the La Mesa de los Santos district in Santander, Colombia. It encompasses two rock shelters near the El Salto del Duende waterfall, which were reportedly used by people associated with the Guane as a burial ground. During a campaign in 1988, led by Professor Arturo Cifuentes from the Universidad Industrial de Santander, students found multiple human remains and artifacts within the caves (*78*). The human remains in question entailed a mummified human body of a possibly high-rank pre-Guane individual inhumated with various grave goods like several rolled blankets, a loom, a baton stick, several ceramic pieces, necklaces, food, and surrounded by scattered human skeletons (*35*). While the mummified body, dating back to 900-1000 BP, showed severe cephalic deformation, only one other skeleton showed paleopathological signs, possibly congenital syphilis (*35, 78*).

Sample selection and sequencing strategy

We screened 21 individuals from five different archeological sites and four different time periods from the Altiplano for ancient DNA preservation. Our sample set encompasses seven individuals from the preceramic site Checua, with an average age for the radiocarbon dated individuals of 6,000 BP, nine individuals form the site Laguna de la Herrera dated to 2,000 BP, two individuals from the early Muisca site Las Delicias dated to 1,200 BP, two individuals from the Guane-associated site Purnia dated to 530 BP, and one individual from the late Muisca site Soacha dated to 520 BP (table S1).

For each individual we constructed double-stranded, double-indexed Illumina libraries (*51*) and evaluated endogenous DNA content via shotgun sequencing. We considered a human endogenous DNA content above 0.1 % in combination with the presence of the characteristic damage pattern for ancient DNA (*55*), as necessary preservation features for in-solution capture (*56*). All screened individuals met these criteria with preservation of human endogenous DNA between 0.31 % and 48 %. Therefore, we proceeded for all individuals with both nuclear and mitochondrial DNA in-solution captures. For individuals with less than 1 % human DNA, we performed two rounds of capture, while for individuals with above 1 %, we performed one round of capture. We reached an average capture efficiency of 25 and 228 for one and two rounds of capture, respectively. Additionally, we sequenced each library multiple times to maximize the number of available SNPs for each individual. With this strategy, we were able to obtain usable genome-wide data for 19 individuals from the Bogotá plateau and two individuals from the Los Curos area, both on the Atiplano Cundiboyacense.
We integrated the genomic data with previously published ancient and modern genomes from the Allen Ancient Genome Diversity project version v.54.1 genotyped on ~1,24M SNPs (table S8.A) (*79*), the Human Origins panel genotyped on ~600K SNPs (table S8.B,C) (*37*) and the Native American Illumina panel genotyped on ~365K SNPs (table S8.C,D) (*23*), resulting in three different datasets to obtain the highest overlapping SNP coverage for different analyses.

Uniparental markers and biological kinship

We observed substantially different mtDNA haplogroup frequencies across time in our dataset corresponding to the observation of a complete genetic replacement of preceramic individuals by an incoming ancestry sometime after 6,000 BP. In the preceramic group, the most prevalent mtDNA haplogroup is A2 and its subgroups, with haplogroup C being present in only one individual (table S1). Even though we were able to establish second- and third-degree kinship between preceramic individuals from Colombia_Checua_6000BP, they do not share the same mtDNA haplogroup, excluding a connection through the maternal line. Instead, we do observe some patterns of Y-haplogroup sharing. Nevertheless, our Checua samples represent a limited subset of the individuals buried at the site, which limits our ability to assess the social structure of the population in greater detail.
Interestingly, individuals from Colombia_LagunadelaHerrera_2000BP show a prevalence of mtDNA haplogroup B2d, with haplogroup C1c being present in two individuals and D1 in one (table S1). This suggests a turnover in mtDNA haplogroup diversity, which is in line with the genetic replacement observed at the nuclear DNA level. In addition, haplogroups B2d and A2w were previously found in pre-Hispanic individuals from Panama, confirming the genetic

link between the post-2,000 BP individuals from the Altiplano and ancient populations from Lower Central America.
We were able to reconstruct a parent-offspring relationship between two female individuals (FORM004 and FORM006) and a second-degree kinship between the female individuals FORM004 and FORM008. Based on our analysis, these females share the same mtDNA haplogroup, namely B2d, which might indicate that they derive from a shared maternal lineage. From 1,200 BP onwards, we observe the survival of mtDNA haplogroup B2d, in agreement with large-scale genetic continuity between ~2,000 and 500 BP in the Altiplano.

Population structure and effective population size

Archeological and anthropological evidence has been used to derive population size dynamics of human groups on the Altiplano. It was suggested that preceramic hunter-gatherer populations lived in relatively small groups and relied on a foraging lifestyle that transitioned towards sedentism through time (*27, 80, 81*). Populations associated with the Herrera ceramics are instead considered to have had larger group size and to be mainly based on a farming subsistence (*80, 81*). With the beginning of the Muisca period, roughly around 1,200 BP, population size increased even further, until it declined again due to demographic impact of the Spanish colonization (*82, 83*).
We investigated the changes in effective population size, as well as mating patterns, by analyzing runs of homozygosity (ROH) and Identity by decent (IBD) using hapROH (*41*). The analysis of the Colombia_Checua_6000BP group shows a large proportion of short (4-8 cM) ROHs in all individuals with a few medium (8-20 cM) and even fewer long (>20 cM) ROHs (fig. S9). This pattern is indicative of a small effective population size rather than a close kin mating pattern. To further expand on this observation, we inferred IBD for male individuals as implemented in HapROH. We were able to detect several short IBD stretches, again indicative of a small effective population size but with limited close kin mating (fig S8).
In individuals from the Colombia_LagunadelaHerrera_2000BP group, ROHs of short and medium size decrease significantly compared to Checua (fig. S9). However, a substantial amount of long ROHs is present in FORM006 and FORM009, inferring a higher level of consanguinity than the other individuals analyzed from the same archeological site. The male individuals from Laguna de la Herrera do not share any IBDs (fig S7), supporting the scenario of a larger effective population size in this site than in Checua.
In all other individuals we observe a general decline in short and medium ROHs through time, which is consistent with the described population size increase from the early Muisca period onwards. Finally, we report a potential case of close-kin mating offspring for GUAN002. The pattern of ROHs is the closest resembling that of two second cousins mating (fig S9).

Principal Component Analysis

Since Principal Component Analysis (PCA) is significantly influenced by missing data, we created three different datasets to build PCAs by merging our generated genomes with the 1240K SNP panel downloaded from the Allen Ancient Genome diversity project (*79*) (table S8.A), two datasets of modern-day Native American individuals genotyped on the Human Origins panel (table S8.B) (*20, 60*) and the unmasked, unadmixed version of the Illumina Native American panel (table S8.D) (*23*). We analyzed these datasets separately to retain as many SNPs as possible while minimizing missing data.

We first built a PCA using present-day genomes from the SGDP panel (*37*) and projected the ancient individuals on this variation to verify their placements within a worldwide genetic diversity map as a visual indicator of potential non-Native American admixture or contamination (fig S6). As expected from their pre-colonial age and the low-level levels of contamination estimated with schmutzi and ANGSD (*57, 58*), all analyzed ancient individuals fall within the genetic diversity of modern Native Americans. This confirms no signs of recent admixture with non-Native American ancestries nor modern-day DNA contamination (table S1 and fig S11).

We further explored the relationship of the generated ancient genomes from Colombia and previously published ancient genome from Panama and Venezuela in relation to the unadmixed and unmasked Native American populations from a dataset published by Reich *et al.* (*23*). We restricted the number of individuals used to build the PCA by selecting populations from the Isthmo-Colombian area and projected the ancient individuals on this modern-day genetic variation (fig. S12). In addition, we projected Ceramic-age and Archaic-age individuals from the Caribbean (*44, 45*) due to general affinities with individuals from the Isthmo-Colombian area revealed through *f*-statistics (table S4.D). In this PCA we find a pattern where ancient Colombians from the Altiplano fall in-between East Colombian populations like Piapoco and Guahibo, and Panamanian and Costa Rican populations like Guna, Cabécar, Teribe and Bribri, as well as ancient Panamanians. Interestingly, when we project Ceramic-age Venezuelans into the same PCA space, these individuals fall on a widespread cline with some being more drawn towards ancient and present-day Isthmus, while others more towards eastern Colombians. As expected from previous studies (*44, 45*). Ceramic-Age Caribbeans are the closest groups to Piapoco, while Archaic-age Caribbeans fall in an intermediate position between Ceramic-Age Caribbeans on one side, and Venezuelans and Colombians on the other. Interestingly, present-day populations from Northern Colombia like Waunana and Embera, but also some Costa Rican populations like Maleku and Guaymi are removed from the main cline and extend along PC2. Lastly, all individuals from the Colombia_Checua_6000BP plot with Archaic- and Ceramic-age individuals from the Caribbean, which is interpreted as an artifact due to the equal genetic distance of this group from all present-day Indigenous populations from South Americans observed in *f*-statistics.

Finally, our analyses showed a distinct affinity of ancient individuals younger than 2,000 BP from the Bogotá plateau to ancient and modern individuals form the Isthmus. Thus, we used previously published genotypes of present-day individuals from Panama (*20*) to build a third PCA and project ancient individuals on this variation (fig. S13). As previously reported by Capodiferro and colleagues, Guna is a highly drifted population, as also visible in our PCA. Instead, the other modern Panamanian populations form two distinct clines, one mainly comprised of Ngabe and Naso individuals, and the other of Embera individuals. Ancient individuals from Panama, Colombia and Venezuela plot close to the center of this triangle, with post-2,000 BP Colombians largely overlapping and ancient Venezuelans placed along a gradient of higher-to-lower affinity to ancient Panamanians. Therefore, this PCA built with a Human Origins dataset confirms the main findings observed in the previous PCA built with an Illumina dataset (fig. S12).

ADMIXTURE

To obtain a general overview of the clustering pattern in different genetic ancestry components of ancient and modern Native American individuals we used ADMIXTURE (Version 1.3.0). We created a dataset comprising the generated ancient genomes from Colombia, previously published ancient Panamanians and Ceramic-age Venezuelans *(20, 44)*, as well a subset of masked individuals from the Illumina dataset *(23)* (fig. S11). We ran ADMIXTURE in unsupervised mode with the number of ancestral populations K ranging from 3 to 15. The calculation of the cross-validation error for each K reveals that K=6 has the lowest value, with a clear increase from K=7 onwards.
Starting from K=3, ancient Colombians as well as Panamanians and Venezuelans are largely comprised of a component shared with Cabecar, one with Quechua/Pima, and a third component virtually shared with all Native South Americans. In the run of K=6 (Fig. 3A), we observe different proportions of a component maximized with Cabecar in ancient Colombians younger than 2,000 BP, Panamanians and Venezuelans, but it is absent in individuals from Checua. Nevertheless, we observed a component shared between all these ancient groups, including Checua, which is maximized in Quechua and Aymara. While ADMIXTURE analysis suggests a shared genetic ancestry component between these ancient and present-day populations, we were unable to confirm this with *f*-statistics (see below). Therefore, we interpret this shared component as the results of an appropriate modeling of the genetic ancestry profile of ancient Colombians.

F-statistics

We conducted multiple *f*-statistics by creating three separate datasets, one containing only ancient and present-day genome-wide data overlapping up to 1.24M SNPs (*79*), one with the masked individuals from the Illumina dataset *(23)* and one with the Human Origins genotypes for present-day Colombian individuals *(60)*.
We initially tested whether the individuals of one site could be grouped by conducting a f_3-outgroup statistic in the form of f_3(Mbuti, Individual 1; Individual 2). We ran this statistic both within and among the sites to exclude the possibility of closer inter-site affinities. We were able to show that there is intra-site homogeneity, and we grouped individuals by site (tables S1 and S2.A-B).
Additionally, we performed a f_3-outgroup statistic of the form f_3(Mbuti, X; Ancient South America), where X stands for our reported individuals and 'Ancient South America' for major genetic ancestry lineages of the sub-continent. We then built a genetic dissimilarity matrix for multidimensional scaling (MDS) through 1-f_3-outgroup statistics (Fig. 3B). To confirm the patterns observed with the f_3-outgroup statistics, we performed f_4-statistics in the form of f_4(Mbuti, X; Ancient Americans, Ancient Americans), with X representing the sequenced ancient Colombian groups (tables S3.C and S4.H).
We then tested for genetic affinities of ancient Colombian individuals with available modern Native American populations through f_3(Mbuti; Ancient Colombia, X), with X being individuals from Reich *et al.* (*23*). We found a strong similarity between ancient Colombians younger than 2,000 BP and modern-day populations from the Isthmus, significantly less affinity to northern Colombian populations, and no direct links to other neighboring populations within and outside of Colombia (Fig. 4A). However, we also observed a generalized affinity of Colombia_Checua_6000BP to all available Central and South Americans, consistent with its placement as a deeply divergent lineage (Fig. 2B). Additionally, despite the limited SNP

overlap between the Illumina and Human Origins panels (~78,000 SNPs), we run f_3(Mbuti; X, Ancient Colombia) combining the Reich et al., (*23*) and Arias *et al*. *(60)* datasets to maximize the number of present-day individuals from the Isthmo-Colombia area. We observe the same pattern of generalized affinity of Colombia_Checua_6000BP with populations from the Isthmus and South America (fig. S20A). Individuals from the Altiplano younger than 2,000 BP confirm a specific affinity to Isthmian populations to the exception of the highly drifted Chorotega group (fig. S20B-E). We further confirmed the differential affinities highlighted with f_3-outgroup statistics in corresponding f_4-outgroup statistics of the form f_4(Mbuti, Ancient Colombia; Modern Native Americans, Modern Native Americans) (tables S3.D and S4.C). Moreover, we conducted a series of f_4-statistics to specifically test for distinct genetic links to Anzick-1- and California Channel Island-related ancestries with f_4(Mbuti, Anzick-1; Chile_Los Rieles_12000BP/Peru_Lauricocha_8600BP, X) and f_4(Mbuti, California Channel Island; Peru_Cuncaicha_4200BP/Peru_Lauricocha_8600BP, X), where X is our generated ancient Colombian data (table S3.A-B).

We then conducted the following f_4-statistics f_4(Mbuti, Ancient Colombia; Modern Northern Colombia, Modern Isthmus) (table S7.K). These reveal a significant affinity of ancient Colombians younger than 2,000 BP to modern-day as well as ancient populations of the Isthmus, Ceramic-age Caribbean, and Ceramic-age Venezuelans with an absence of significant affinity towards modern northern Colombian populations.

Furthermore, we tested whether other modern-day Colombians provide a better proxy for ancient Colombian than either modern Northern Colombians or modern Isthmus populations by performing two f_4-statistics of the form f_4(Mbuti, Ancient Colombians; Modern Colombians, Modern Northern Colombians) and f_4(Mbuti, Ancient Colombians; Modern Colombians, Modern Isthmus) (table S7.D).

Lastly, we tested whether ancient and modern-day populations from the Isthmus are equally related to ancient Colombians by conducting the following f_4-outgroup statistic f_4(Mbuti, Ancient Colombians; Ancient Isthmus, Modern Isthmus). By extension we also tested whether ancient Colombians and Isthmian populations exhibit differential affinity to the modern-day Isthmus groups with f_4(Mbuti, Modern Isthmus; Ancient Colombians, Ancient Isthmus) (table S7A-J).

The affinity to Ceramic-age Venezuela was tested in multiple ways, firstly in relation to populations from the Isthmus and the Ceramic-age Caribbeans. We tested whether ancient and modern-day Isthmus populations remain the best proxy compared to Ceramic-age Venezuelans with f_4(Mbuti, Ancient Colombians; Ancient Isthmus, Ceramic-age Venezuela) and f_4(Mbuti, Modern Americans; Ancient Colombians, Ceramic-age Venezuela) (table S4.G-H). In addition, we investigated the Ceramic-age Caribbean affinity with the following f_4-statistics f_4(Mbuti, Ancient Colombians; Ceramic-age Venezuela, Ceramic-age Caribbean) and f_4(Mbuti, Ancient Colombians; Ancient Isthmus, Ceramic-age Caribbean) (table S4.I-J).

qpGraph

The *f*-statistics performed on our reported ancient Colombian individuals from Colombia_Checua_6000BP showed a generalized genetic affinity to all previously sequenced ancient and modern-day Native South Americans, and lack of Anzick-1 and California Channel Island-related ancestries. We therefore hypothesize these individuals being part of the initial South American radiation event. In fact, a previous study *(8)* has proposed that the ancestral population of South Americans likely experienced a rapid diversification process giving rise to multiple South American lineages. However, there was

no genome-wide data available for hunter-gatherer individuals from northern South America, making it difficult to assess where this process took place. Here, we attempted to model Colombia_Checua_6000BP within this previously defined admixture graph topology using the Admixtools package qpGraph (v7.0.2).
We tested multiple placements of the Colombia_Checua_6000BP group, placing it as: 1) a lineage splitting within the North American variation; 2) as part of the radiation event; 3) splitting off from each lineage leading to distinct South American ancestries (fig. S10). Placing Colombia_Checua_6000BP as a lineage splitting from a North American source, yields a worst Z-score that deviates significantly from zero (Z=-5.1), indicating a non-viable model (fig. S10J). Instead, when positioning Colombia_Checua_6000BP as deriving from a basal split before the South American radiation we obtain the best available Z-score of 3.2 but with an internal edge length of 0 (fig. S10A). This Z-score value is maintained when modeling Colombia_Checua_6000BP as deriving from the radiation (fig. S10B-D, S10G). Instead, worst Z-score values are obtained when Colombia_Checua_6000BP is placed as derived within one of the South American lineages (fig. S10E-F, S10H). We therefore consider as the most viable model the one where Colombia_Checua_6000BP derive from the initial radiation event into South America, suggesting that this process took place latest as human groups reached the southern sub-continent.

TreeMix

We used TreeMix v.1.13 to better understand the relative relationships between ancient Colombians, Panamanians and Venezuelans in our dataset, in comparison to Ceramic-and Archaic-age Caribbeans. Previous analyses provide evidence for a significant genetic heterogeneity within the Ceramic-age Venezuelan group, where three individuals show a higher attraction to ancient Panamanians compared to the other five individuals from the same archeological site and similar date (table S4.G). We therefore subdivided this group into two clusters, Venezuela_LasLocas_Ceramic_1 and Venezuela_LasLocas_Ceramic_2, with the latter including the three individuals with more Panamanian-related ancestry.
For each Treemix analysis we included an outgroup, Ceramic- and Archaic-age individuals from the Caribbean, ancient Panamanians plus one ancient Colombian group and one Venezuelan group at the time to exclude the possibility of distorting the analysis by intra-site genetic attraction (fig. S14-S18). We used USA_Ancient_Beringian (*2*) at the outgroup, due to its equal genetic distance to most ancient and modern American populations. Earlier publications have established Archaic-age and Ceramic-age populations from the Caribbean to be largely homogenous populations with little to no admixture between them (*44, 45*). To keep complexity to a minimum, we included only one group for each Caribbean-related ancestry. In f_4-outgroup statistics, we have observed Cuba_PlayadelMango_Archaic to reveal some affinity with ancient Venezuelan and Colombian groups, and thus we specifically chose this population as a representative for the Archaic-age populations (table S4). To represent the Ceramic-age Caribbean group, we chose the best covered site in terms of number of individuals available (Dominican_LaCaleta_Ceramic). For each tree composition we inferred admixture edges from 0 to 5.
When performing the analysis including Colombia_Checua_6000BP as the ancient Colombian group, all trees reveal it as an outgroup to the exclusion off all other populations (except USA_Ancient_Beringian). This confirms the modeled placement of Colombia_Checua_6000BP in qpGraph. Additionally, some admixture edges suggest the contribution of an ancient source into Venezuela_LasLocas_Ceramic_1 and

Colombia_Checua_6000BP. However, we were unable to confirm such link with *f*-statistics or ADMIXTURE analysis (fig. S14).

The group Colombia_LagunadelaHerrera_2000BP exhibits a differential behavior when modelled in relation to both ancient Venezuelan clusters. When analyzed with cluster 1, individuals from Laguna de la Herrera form a sister group with ancient Panamanians. Instead, when plotted with cluster 2, ancient Venezuelans form a siter group with ancient Panamanians. Admixture edges infer gene flow between ancient Venezuelans, Panamanians and Colombia_LagunadelaHerrera_2000BP. However, we were unable to infer directionality or direct admixture proportions with other analyses. This observed pattern extents to Colombians between 1,200 BP and 520 BP (fig. S15-S18).

We interpret this pattern as a cline in the proportion of Chibchan-related ancestry in ancient Venezuelans and post-2,000 BP ancient Colombians. This suggests that Chibchan-related genetic contributions in Venezuela and Colombia underwent different demographic processes.

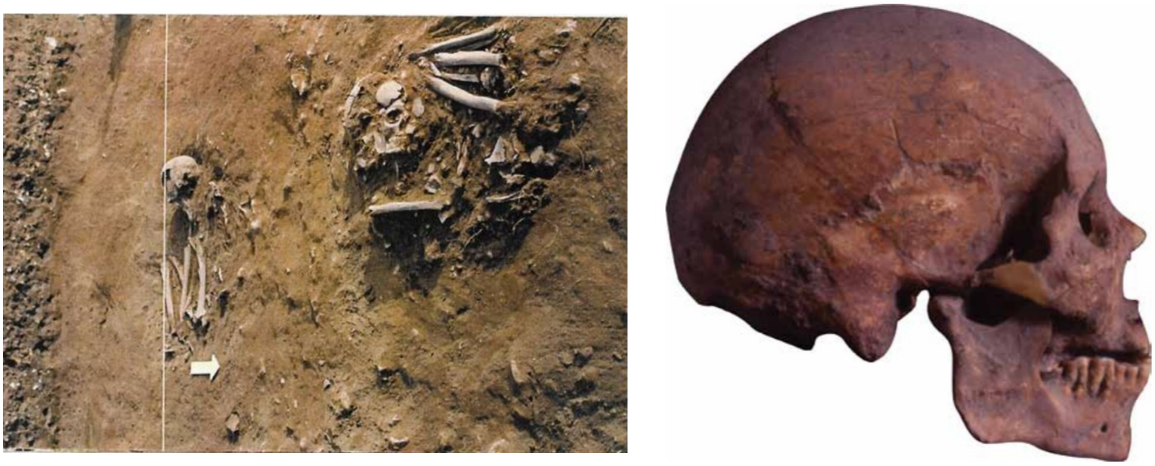

Figure S1: Photographs taken of the archeological site Checua and excavated human remains. Pictures of the Checua archeological site (left) and of a largely complete skull of a hunter-gatherer individual excavated at the site (right), Photo Credit: Jose-Vicente Rodriguez Cuenca, Universidad Nacional de Colombia (84).

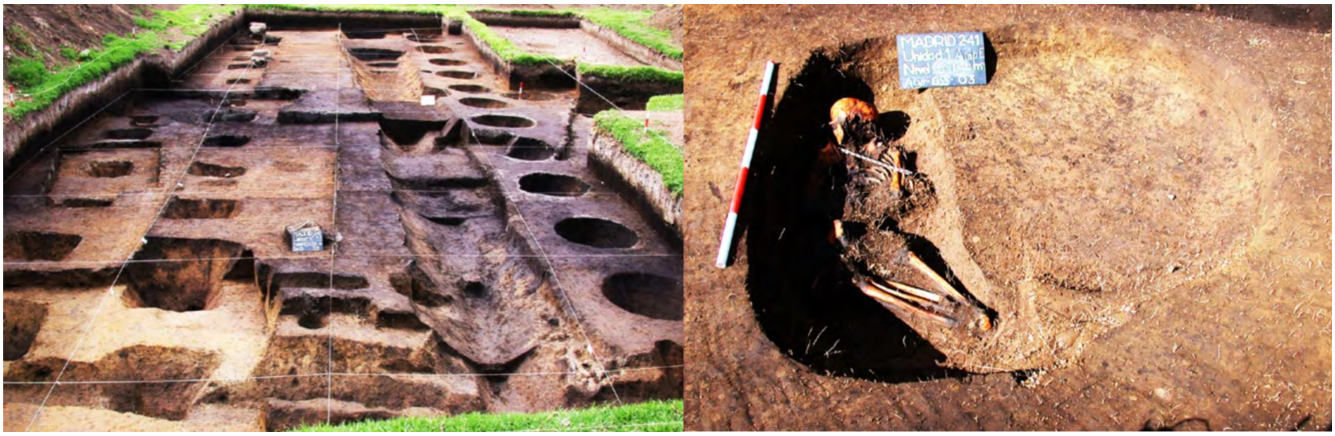

Figure S2: Excavation photograph of Madrid and excavated human remains *in situ*. Pictures of Madrid 2-41 in the Laguna de La Herrera archeological site (left) and of an almost complete skeleton (UE1-F6) excavated at the site (right), Photo Credit: Jose-Vicente Rodriguez Cuenca, Universidad Nacional de Colombia (84).

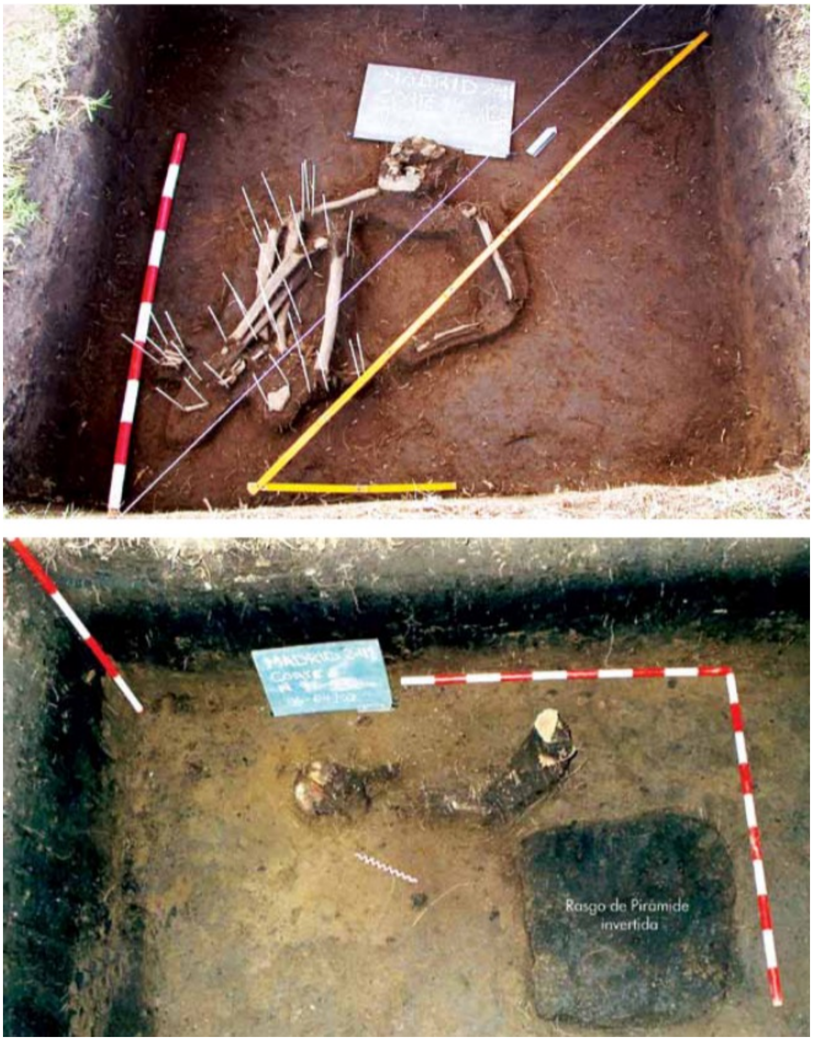

Figure S3: Human remains *in situ* excavated at Madrid. Burial 18 of an incomplete skeleton (above) and infant burial (below) at Madrid 2-41, Cundinamarca, Photo Credit: Jose-Vicente Rodriguez Cuenca, Universidad Nacional de Colombia (84).

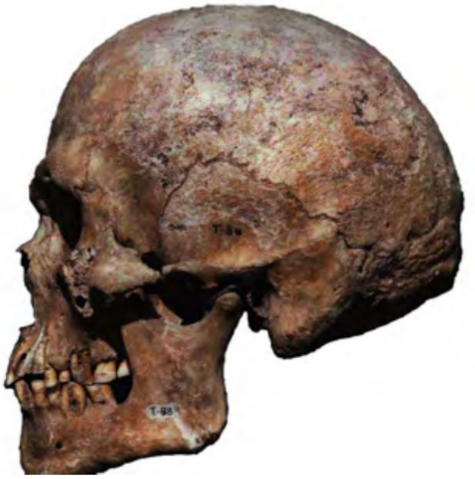

Figure S4: Picture of an almost complete skull. The individual was excavated at the Soacha site in Colombia and is associated with the Muisca culture, Photo Credit: Jose-Vicente Rodriguez Cuenca, Universidad Nacional de Colombia (84).

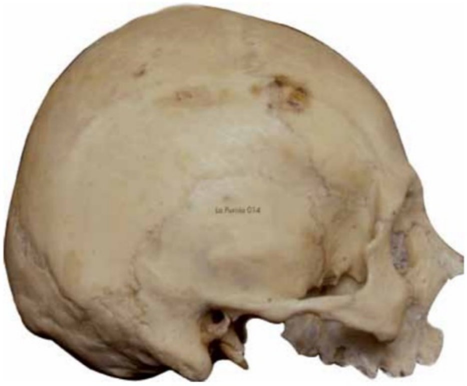

Figure S5: Picture of a skull without mandible. This individual was excavated at the Purnia site in the Los Curos area and was associated with the Guane culture, Photo Credit: Jose-Vicente Rodriguez Cuenca, Universidad Nacional de Colombia (84).

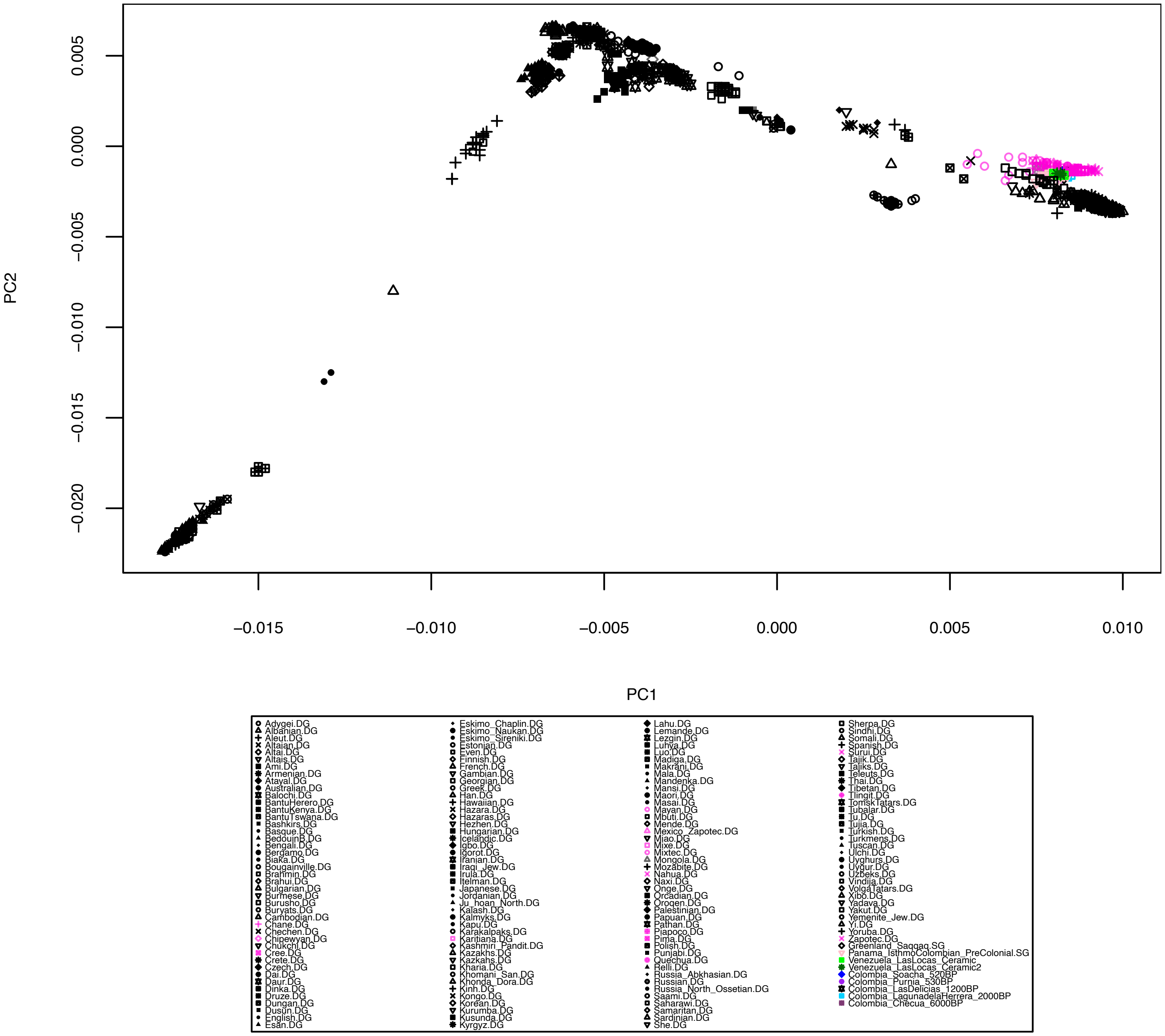

Figure S6: Principal Component Analysis built on worldwide present-day genetic diversity Mallick et al. (37). Ancient individuals were projected. Native American populations are depicted in yellow, other present-day individuals in black and ancient individuals in other colors.

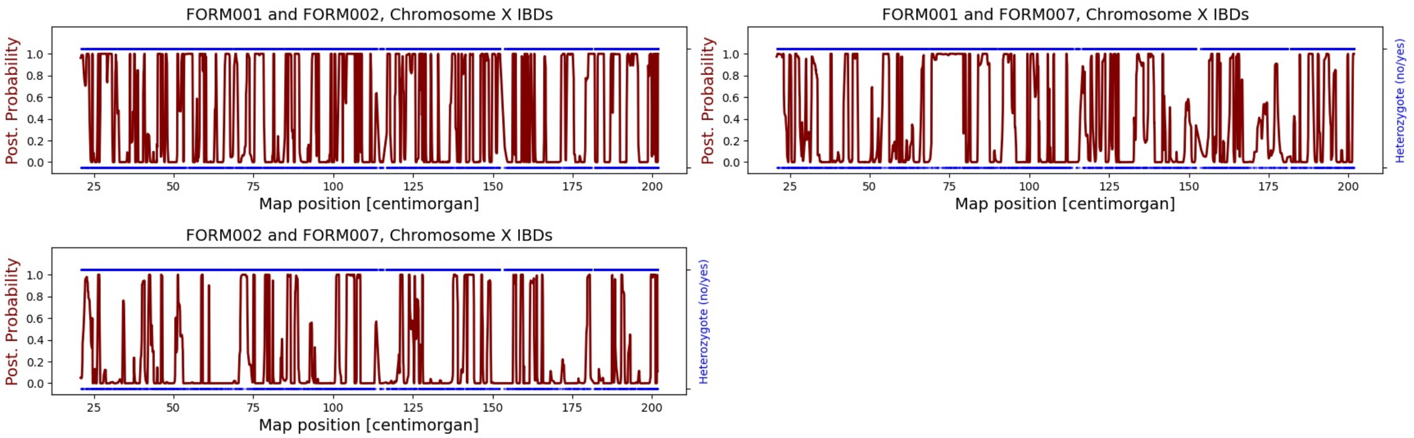

Figure S7: Identity by Descent (IBD) assigned with hapROH Ringbauer et al. (41). Assignment was based on the X-Chromosome for pairs of male individuals within the Colombia_LagunadelaHerrera_2000BP group.

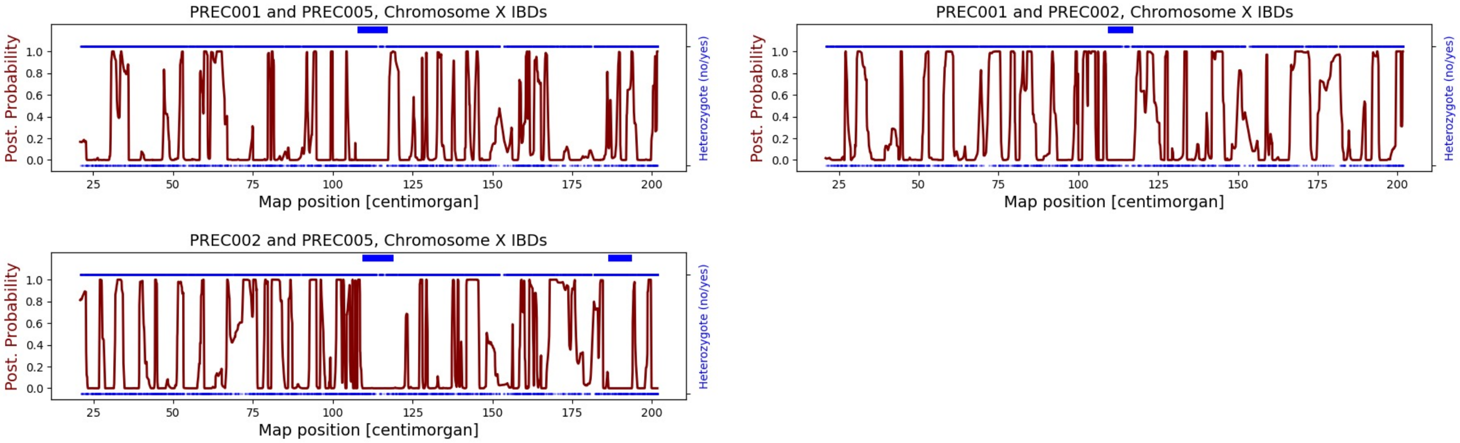

Figure S8: Identity by Descent (IBD) assigned with hapROH Ringbauer et al. (41). Assignment was based on the X-Chromosome for pairs of male individuals within the Colombia_Checua_6000BP group.

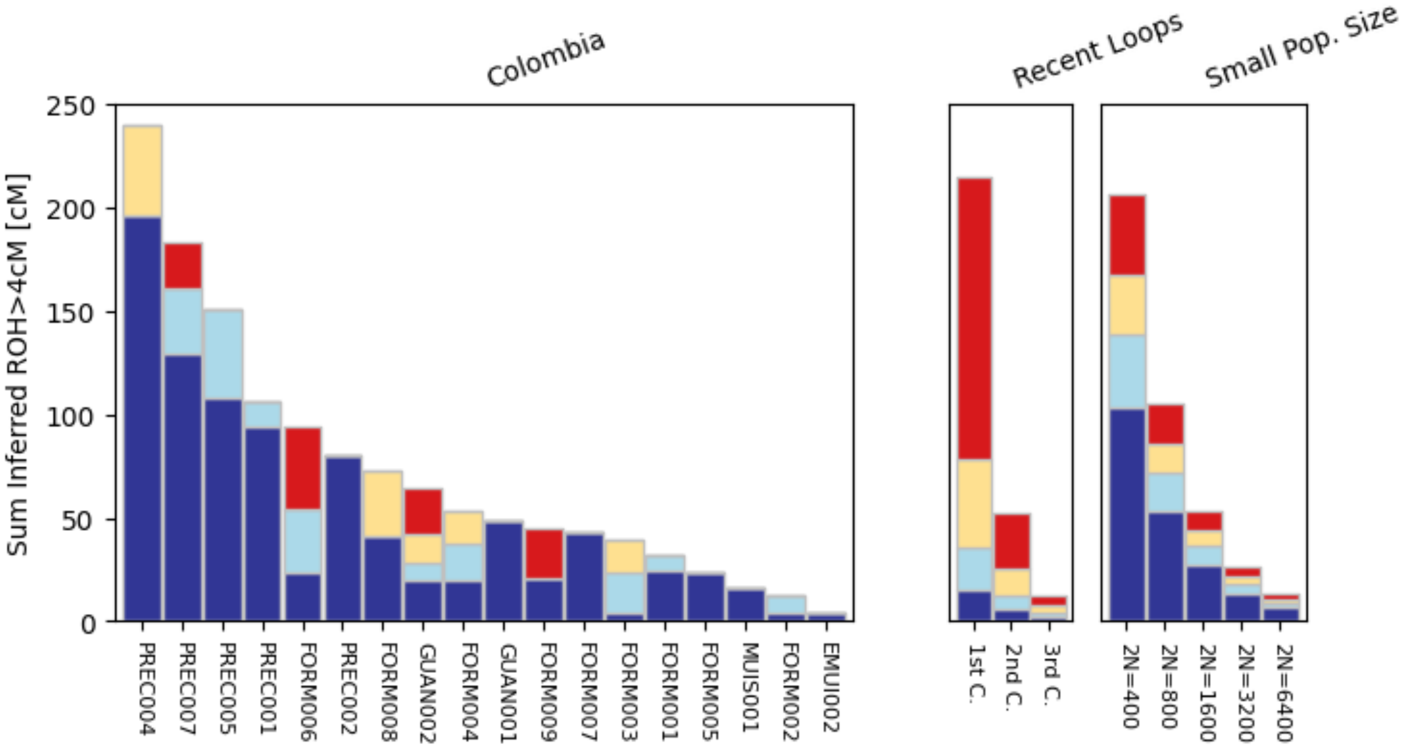

Figure S9: Runs of Homozygosity (ROH) assigned with hapROH for every individual, Ringbauer et al. (41). Individual IDs are indicated in Supplementary Table 1. Color code refers to ROH lengths as follows: dark blue (4-8 cM), light blue (8-12 cM), yellow (12-20 cM), red (20-300 cM).

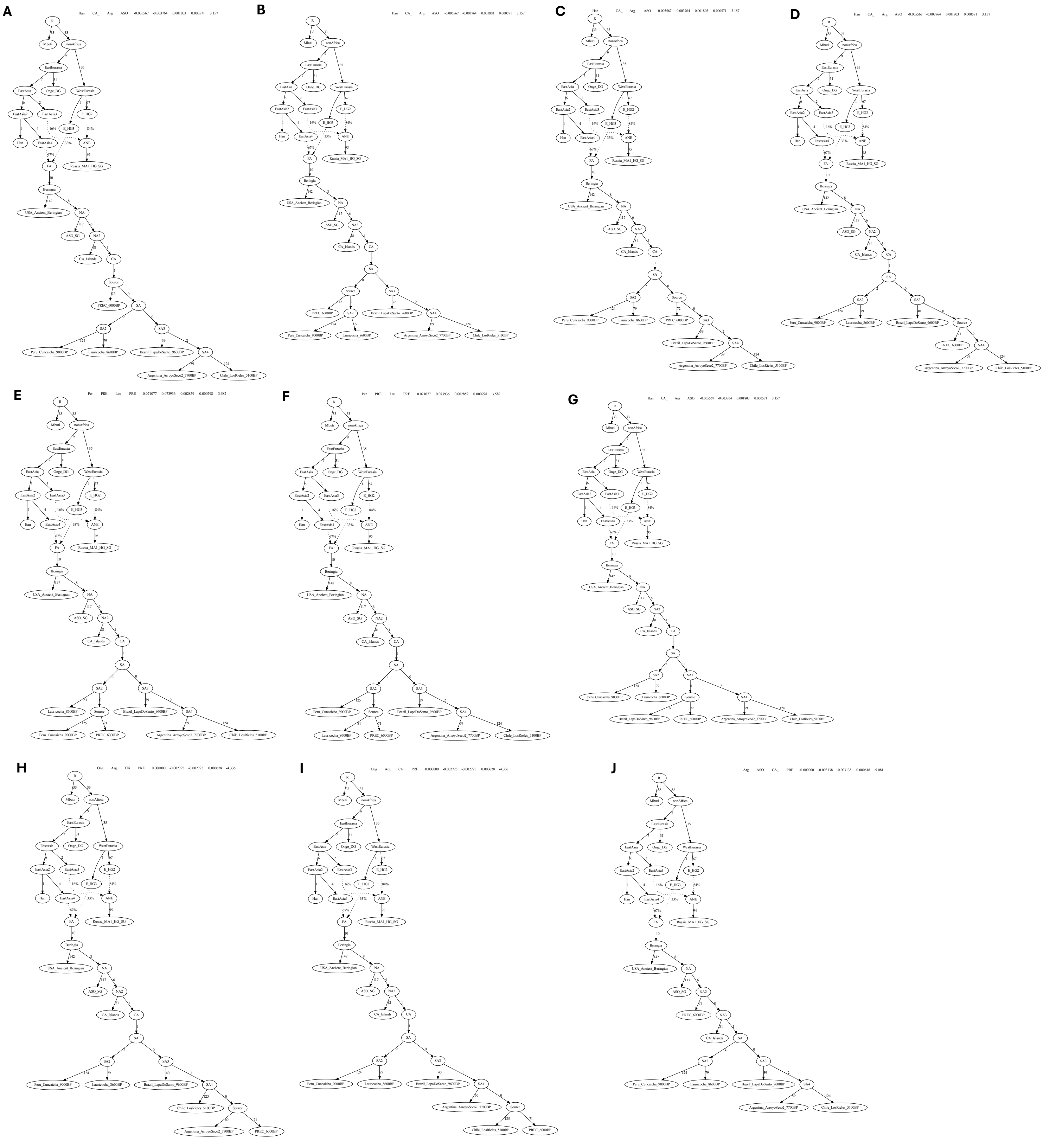

Figure S10: Admixture graph of Colombia_Checua_6000BP in multiple positions among the modelled North and South American genetic lineages. Colombia_Checua_6000BP is here indicated as “PREC_6000BP”. Each panel (A-J) shows a different branching of the Checua group from a fixed tree scaffold topology.

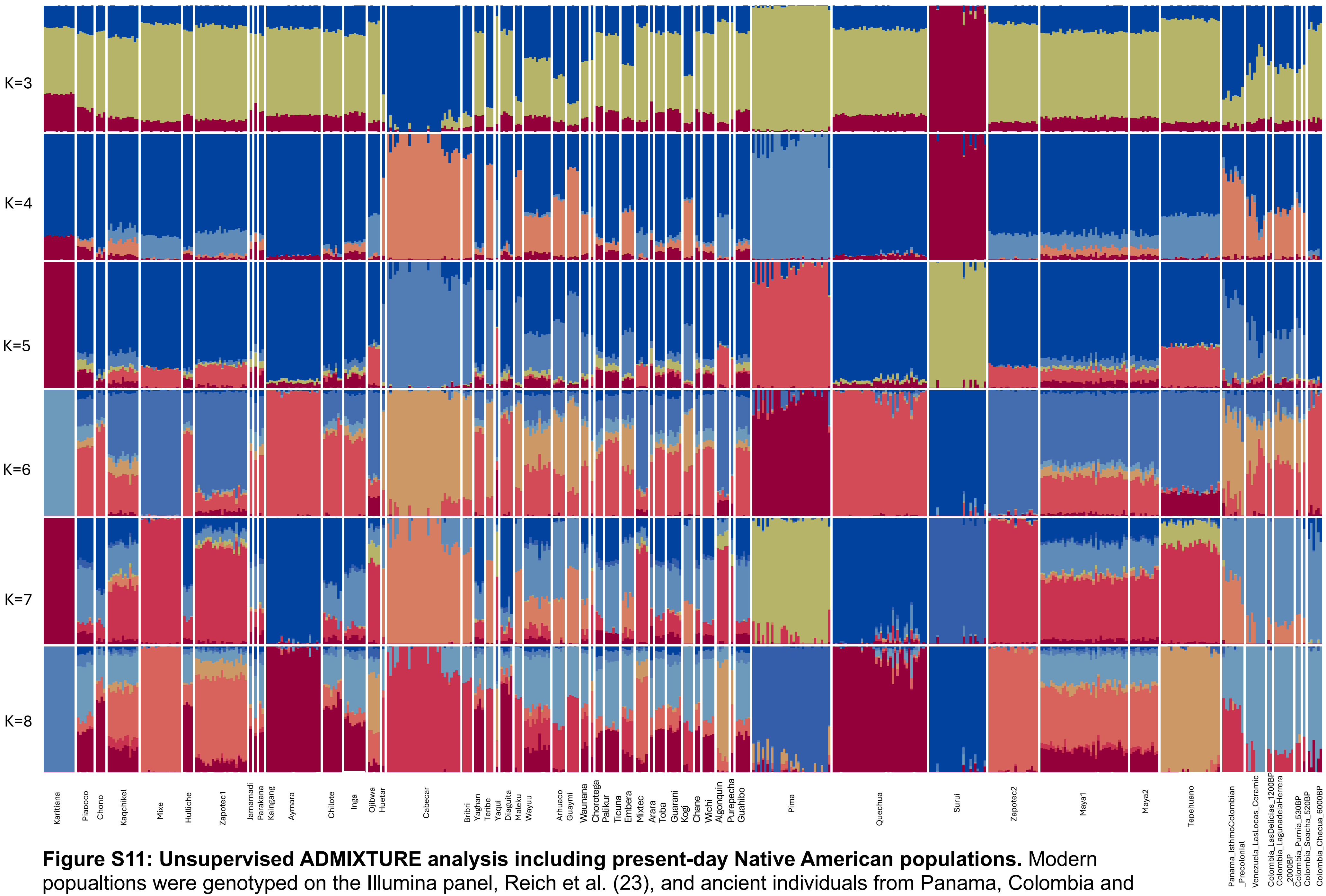

Figure S11: Unsupervised ADMIXTURE analysis including present-day Native American populations. Modern popualtions were genotyped on the Illumina panel, Reich et al. (23), and ancient individuals from Panama, Colombia and Venezuela on the 1240K. Analyses are ranging from K=3 to K=8.

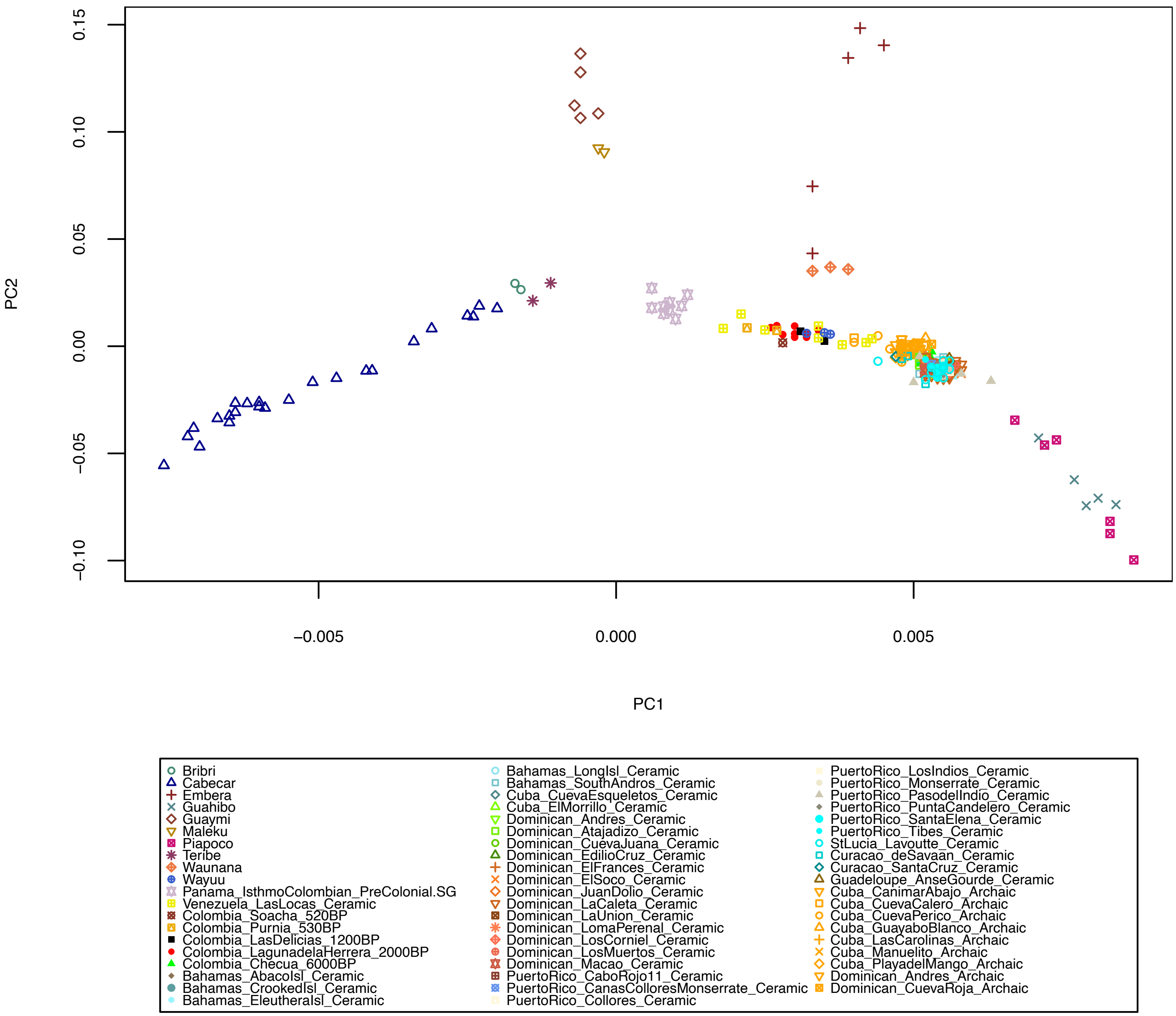

Figure S12: Principal Component Analysis built with the genetic variation of a subset of modern-day individuals from the unmasked and unadmixed Illumina dataset, Reich et al. (23). Ancient individuals were projected onto the PCA.

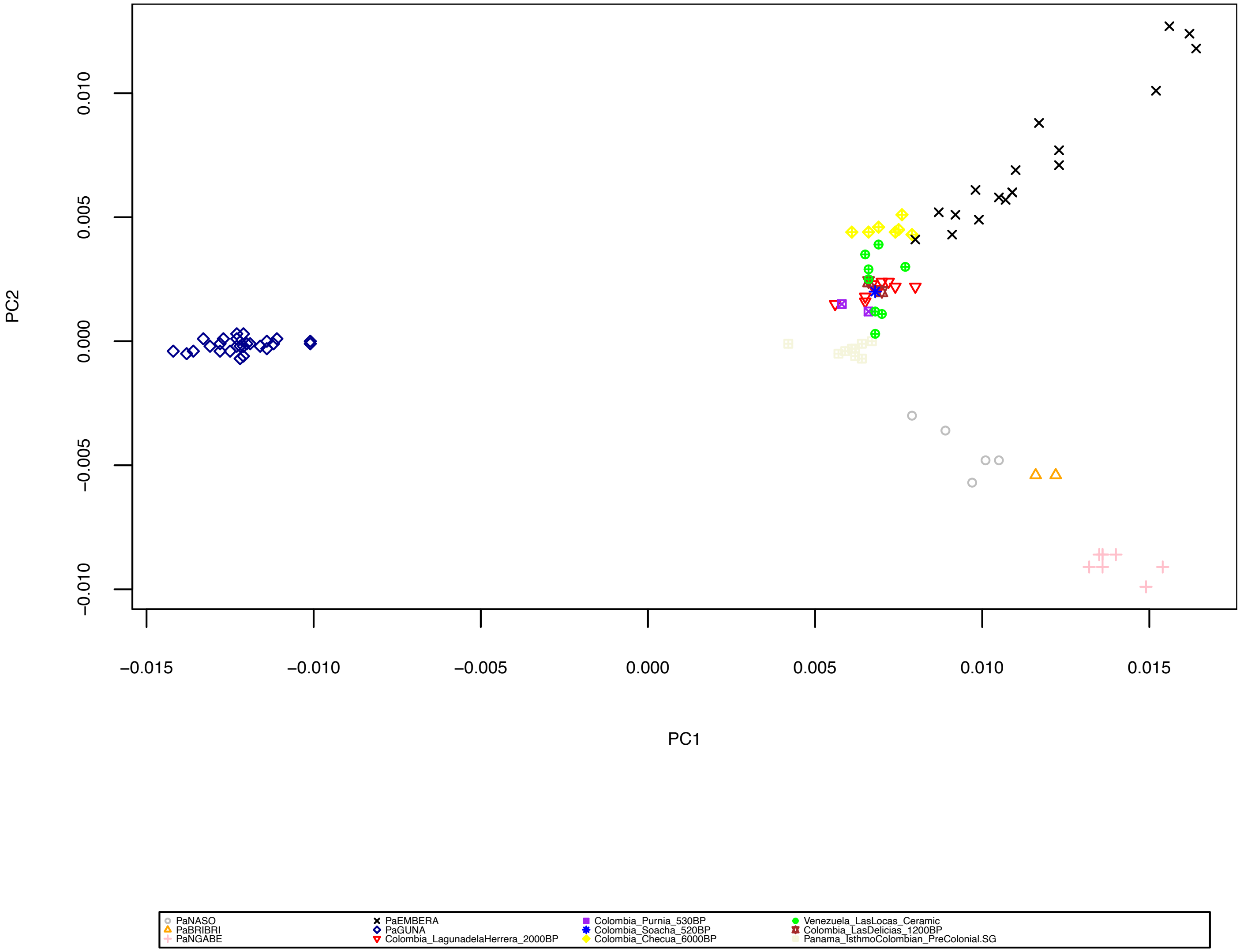

Figure S13: Principal Component Analysis built with the genetic variation of unadmixed modern-day Panamanian individuals genotyped on the Human Origins panel, Capodiferro et al. (20). Ancient individuals were projected onto the built PCA.

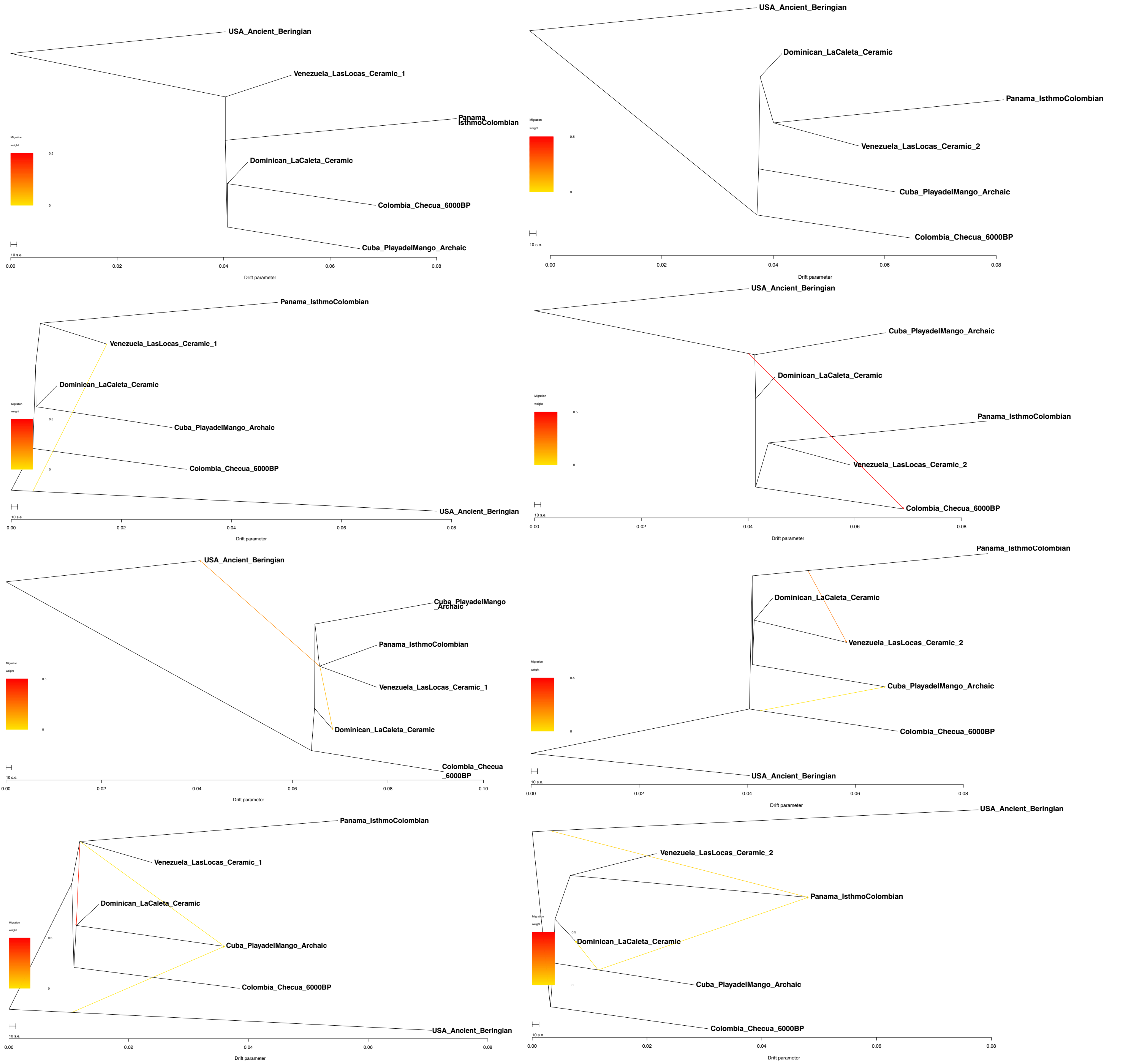

Figure S14: Treemix analysis of the Colombia_Checua_6000BP group, including ancient individuals from Panama and Ceramic-age Venezuela. Trees have been constructed using USR1 as an outgroup and one representative of Ceramic-age and Archaic-age Caribbean populations. Due to their heterogenous genetic profile, Ceramic-age Venezuelan individuals were subdivided into two groups, namely Venezuela_LasLocas_Ceramic_1 (left) and Venezuela_LasLocas_Ceramic_2 (right). Trees are reported from 0 to 3 admixture edges (from top to bottom).

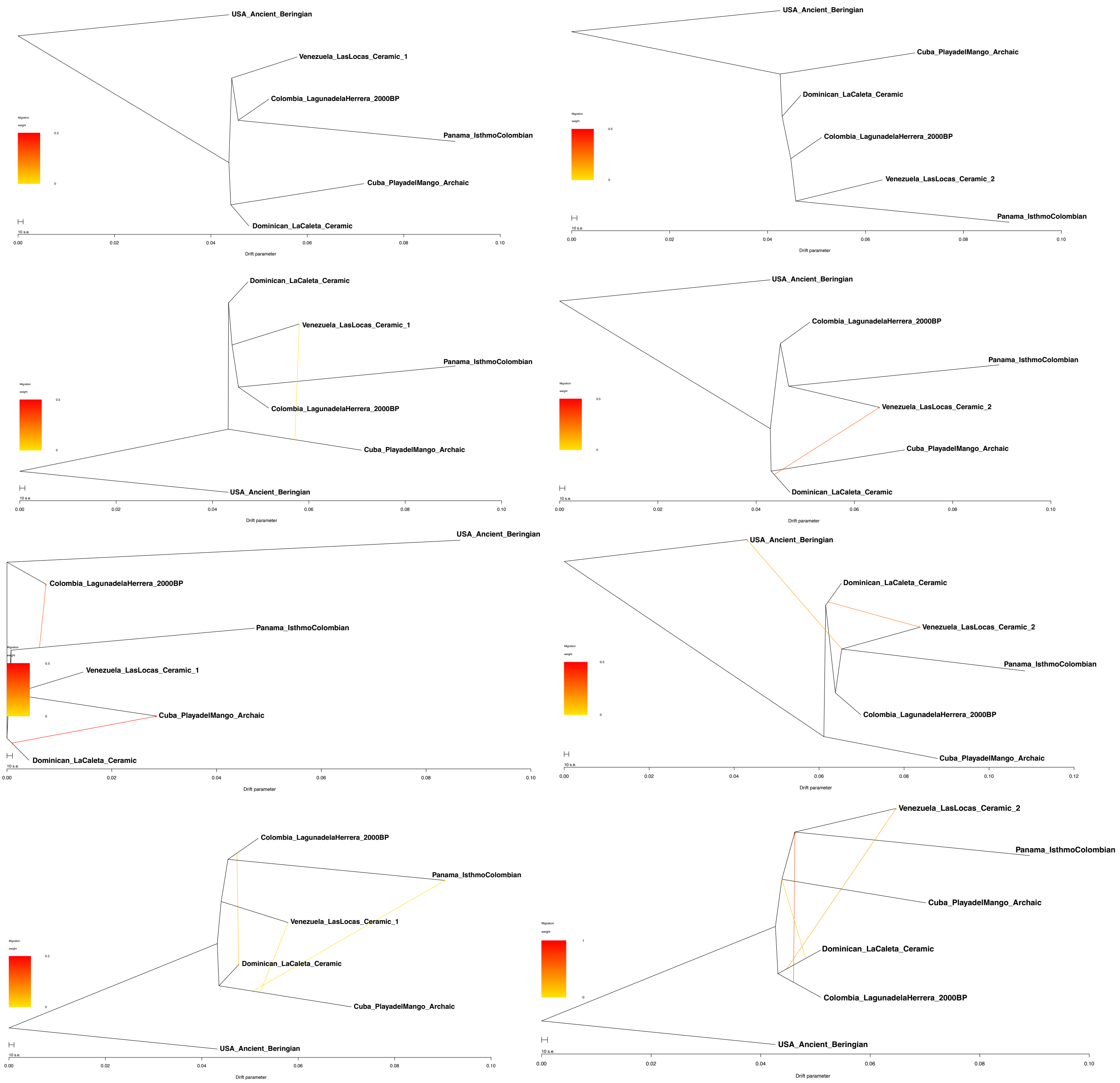

Figure S15: Treemix analysis of the Colombia_LagunadelaHerrera_2000BP group, including ancient individuals from Panama and Ceramic-age Venezuela. Trees have been constructed using USR1 as an outgroup and one representative of Ceramic-age and Archaic-age Caribbean populations. Due to their heterogenous genetic profile, Ceramic-age Venezuelan individuals were subdivided into two groups, namely Venezuela_LasLocas_Ceramic_1 (left) and Venezuela_LasLocas_Ceramic_2 (right). Trees are reported from 0 to 3 admixture edges (from top to bottom).

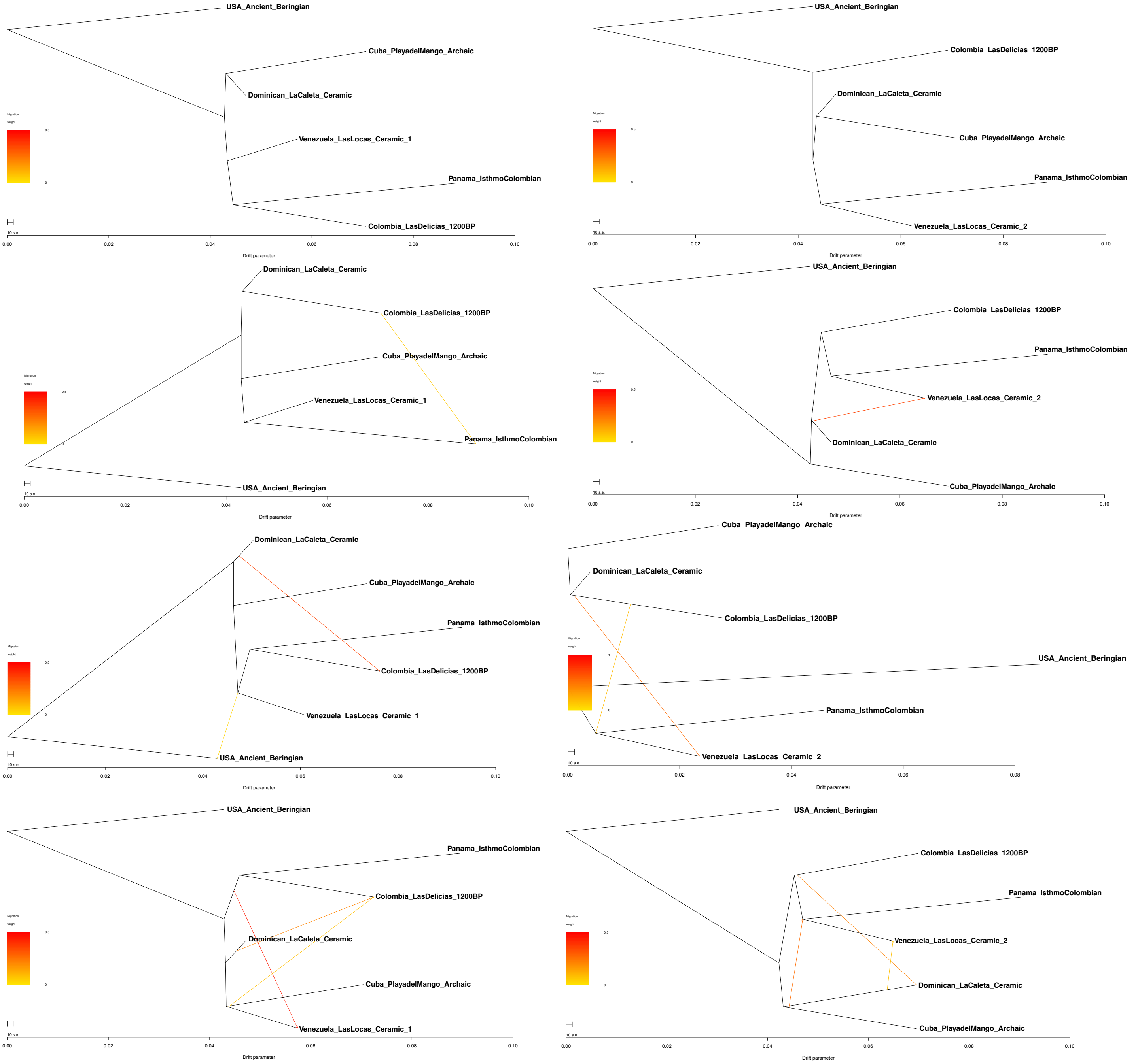

Figure S16: Treemix analysis of the Colombia_LasDelicias_1200BP group, including ancient individuals from Panama and Ceramic-age Venezuela. Trees have been constructed using USR1 as an outgroup and one representative of Ceramic-age and Archaic-age Caribbean populations. Due to their heterogenous genetic profile, Ceramic-age Venezuelan individuals were subdivided into two groups, namely Venezuela_LasLocas_Ceramic_1 (left) and Venezuela_LasLocas_Ceramic_2 (right). Trees are reported from 0 to 3 admixture edges (from top to bottom).

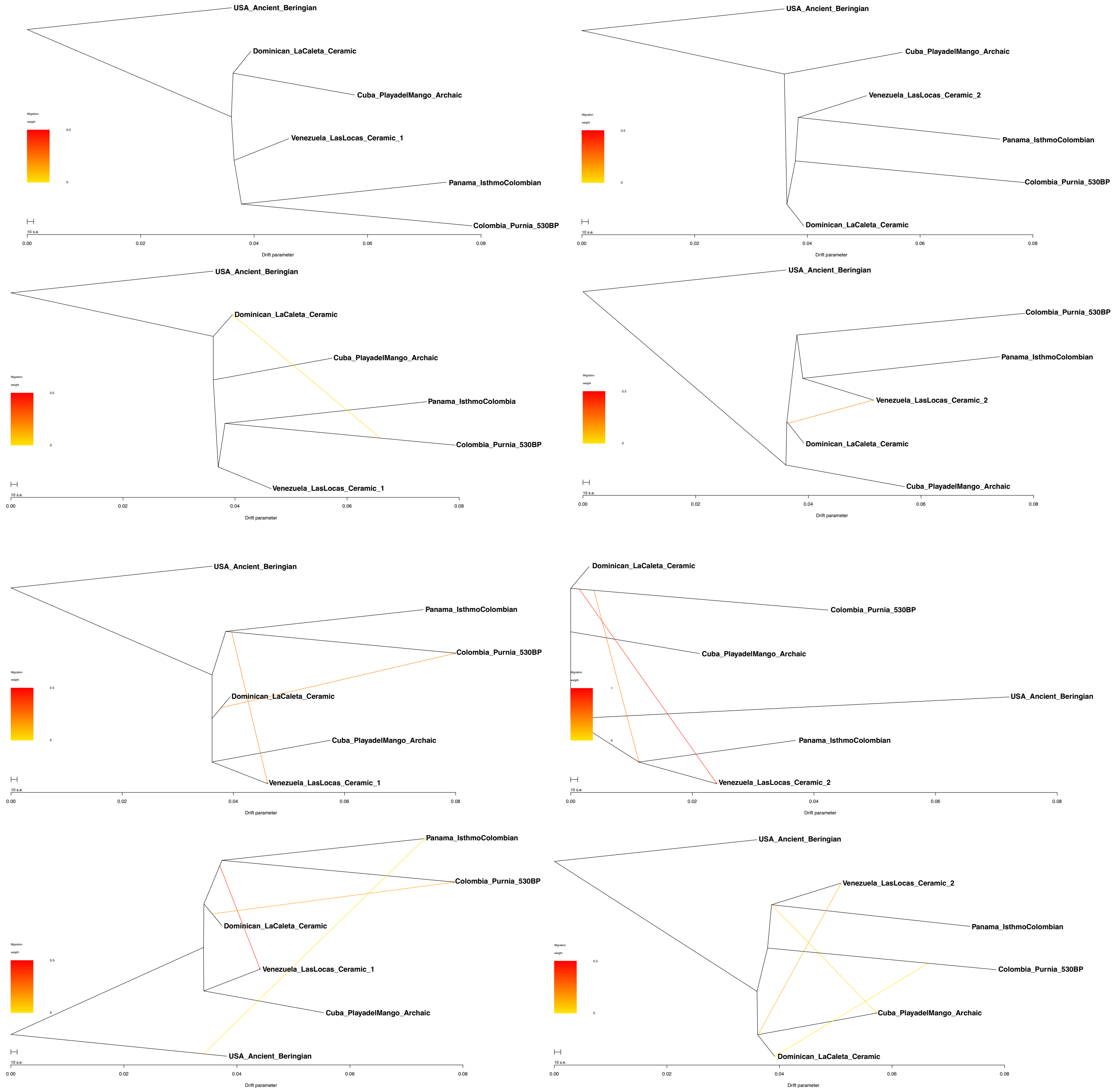

Figure S17: Treemix analysis of the Colombia_Purnia_530BP group, including ancient individuals from Panama and Ceramic-age Venezuela. Trees have been constructed using USR1 as an outgroup and one representative of Ceramic-age and Archaic-age Caribbean populations. Due to their heterogenous genetic profile, Ceramic-age Venezuelan individuals were subdivided into two groups, namely Venezuela_LasLocas_Ceramic_1 (left) and Venezuela_LasLocas_Ceramic_2 (right). Trees are reported from 0 to 3 admixture edges (from top to bottom).

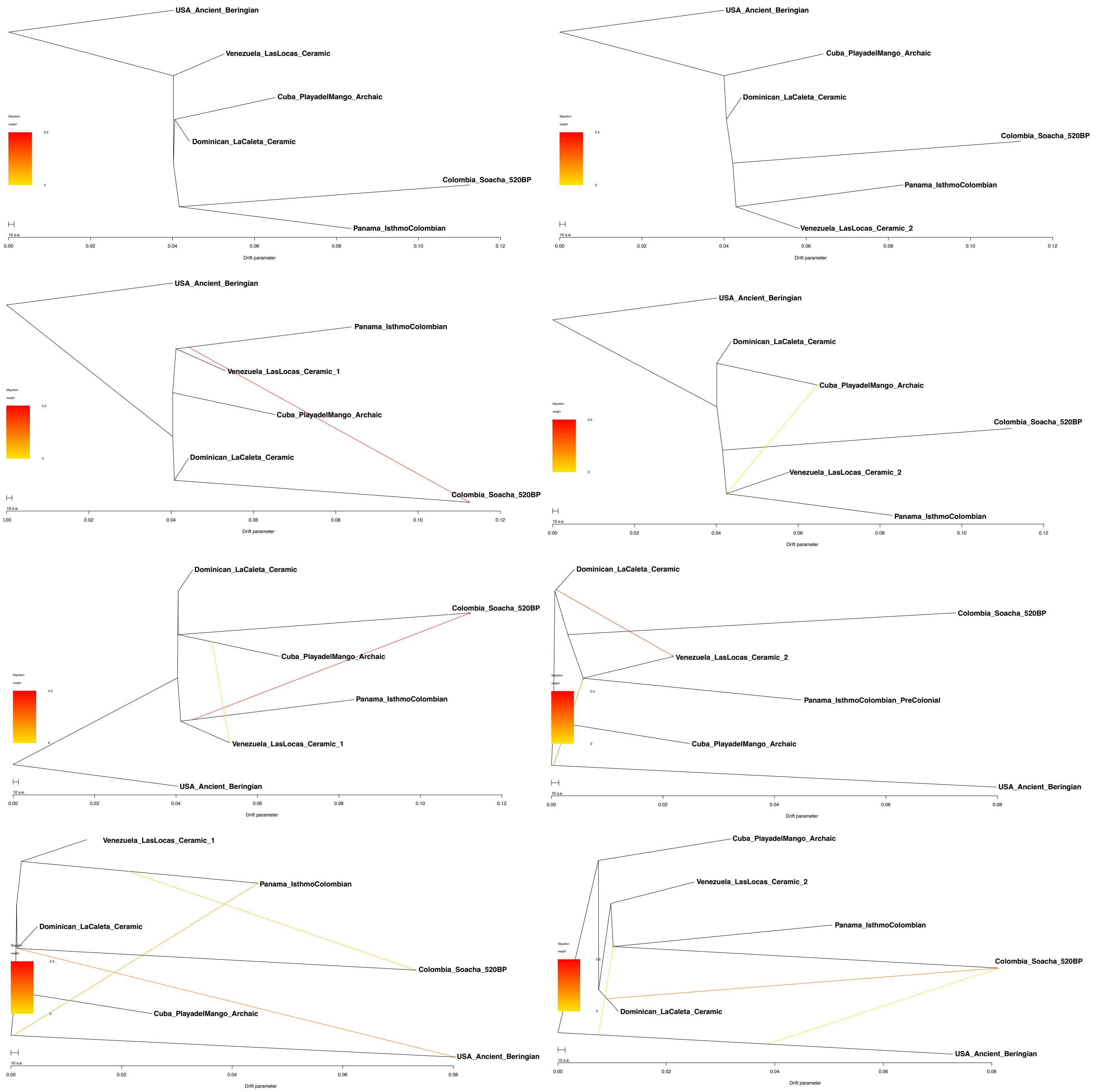

Figure S18: Treemix analysis of Colombia_Soacha_520BP, including ancient individuals from Panama and Ceramic-age Venezuela. Trees have been constructed using USR1 as an outgroup and one representative of Ceramic-age and Archaic-age Caribbean populations. Due to their heterogenous genetic profile, Ceramic-age Venezuelan individuals were subdivided into two groups, namely Venezuela_LasLocas_Ceramic_1 (left) and Venezuela_LasLocas_Ceramic_2 (right). Trees are reported from 0 to 3 admixture edges (from top to bottom).

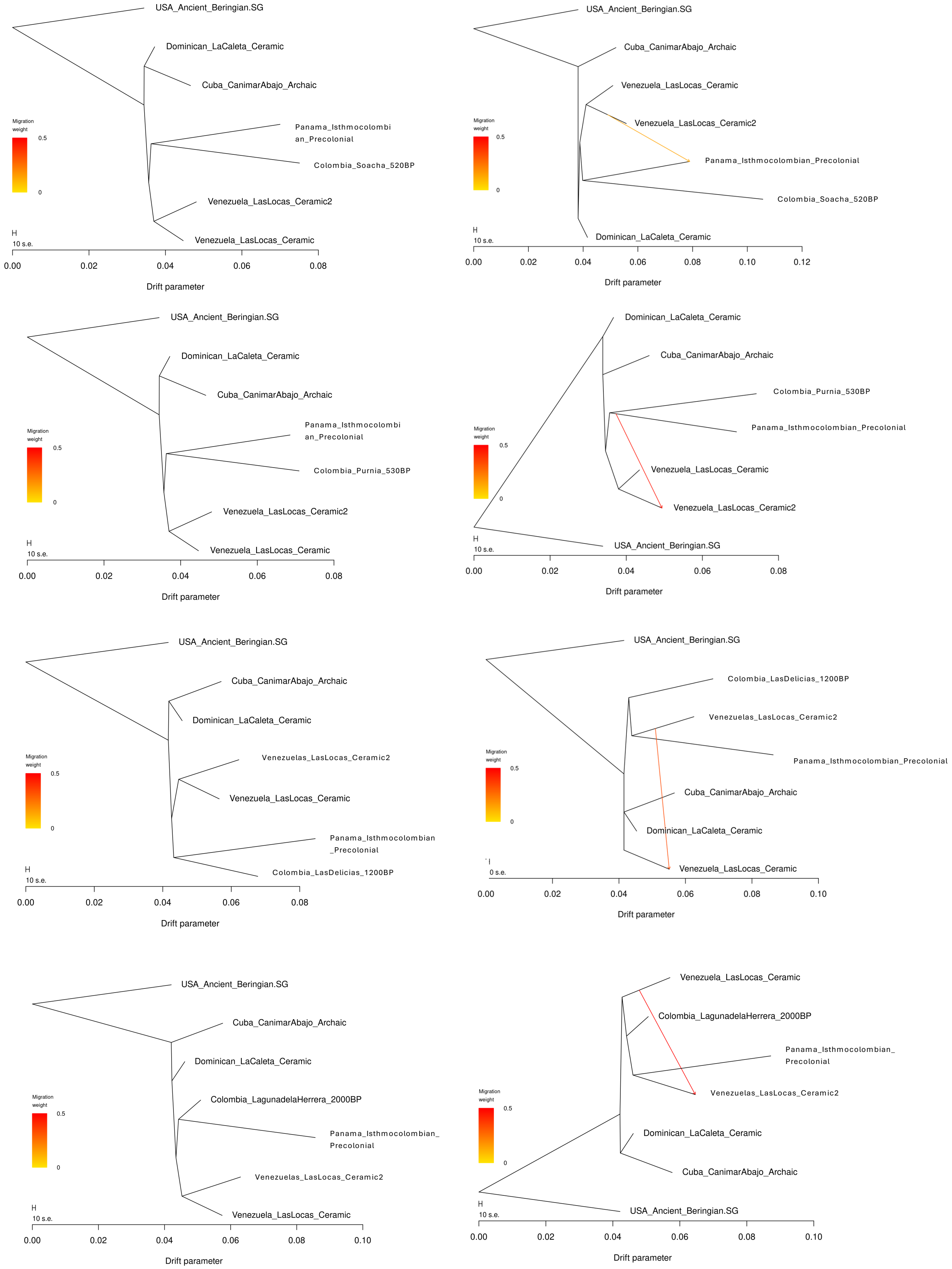

Figure S19: Treemix analysis of ancient Colombians, including ancient individuals from Panama and both Ceramic-age Venezuela groups (Venezuela_LasLocas_Ceramic_1 and Venezuela_LasLocas_Ceramic_2). Trees have been constructed using USR1 as an outgroup and one representative of Ceramic-age and Archaic-age Caribbean populations. Trees are reported for all four post-2000BP ancient Colombian groups, and from 0 to 1 admixture edges (left and right panels, respectively).

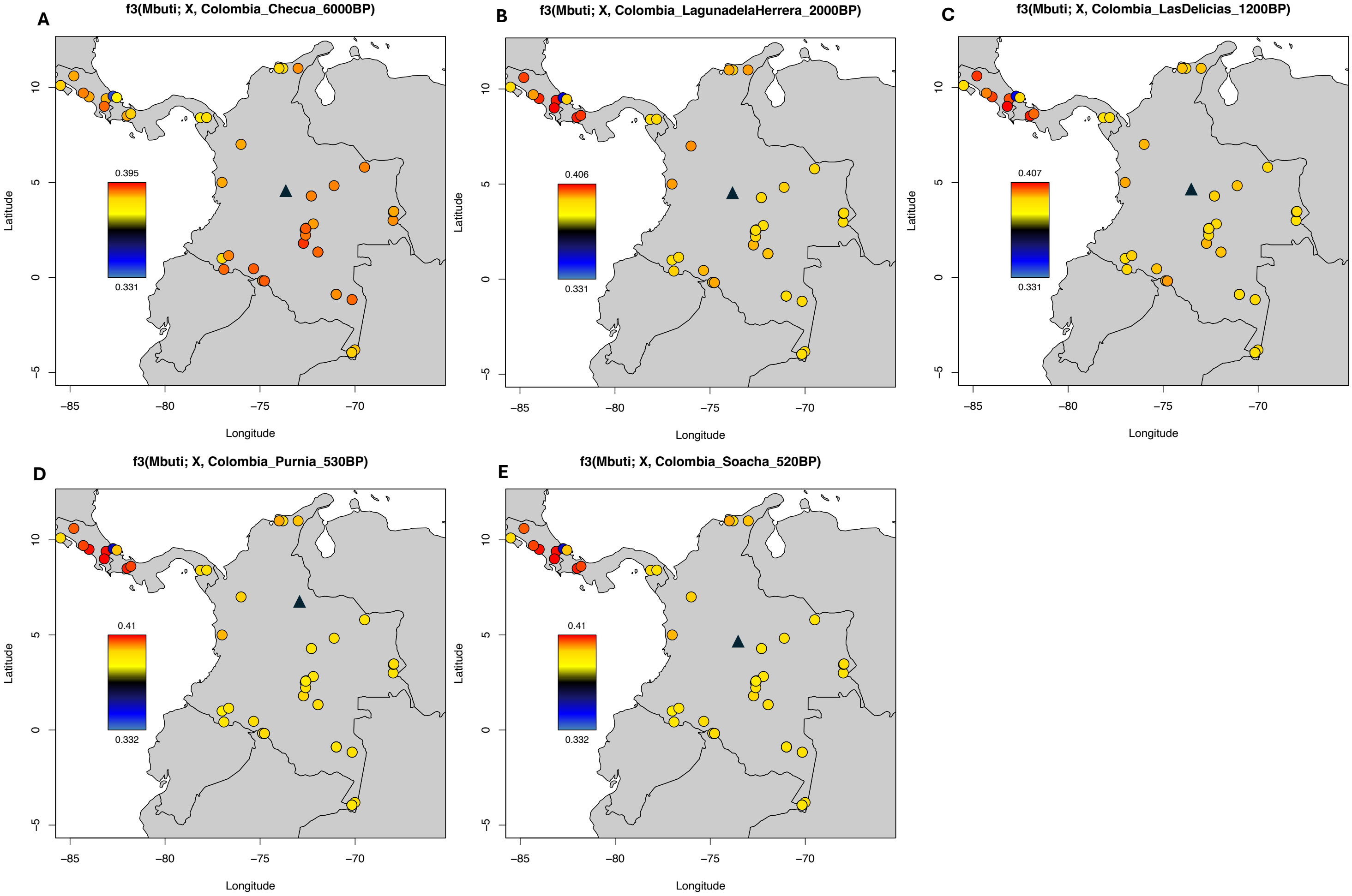

Figure S20: Map with *f3*-outgroup statistics measuring the shared genetic drift of ancient Colombian individuals with present-day Indigenous populations from Panama and Costa Rica. The Costa Rican populations were genotyped on the Illumina panel, Reich et al. (23), and the Panamanian and Colombian populations were genotyped on the Human Origins panel, Capodiferro et al. (20) and Arias et al. (60). Each panel (A-E) reports the *f3*-outgroup statistics of present-day populations (indicated with colored circles) with different ancient groups analyzed in this study (indicated with a black triangle).

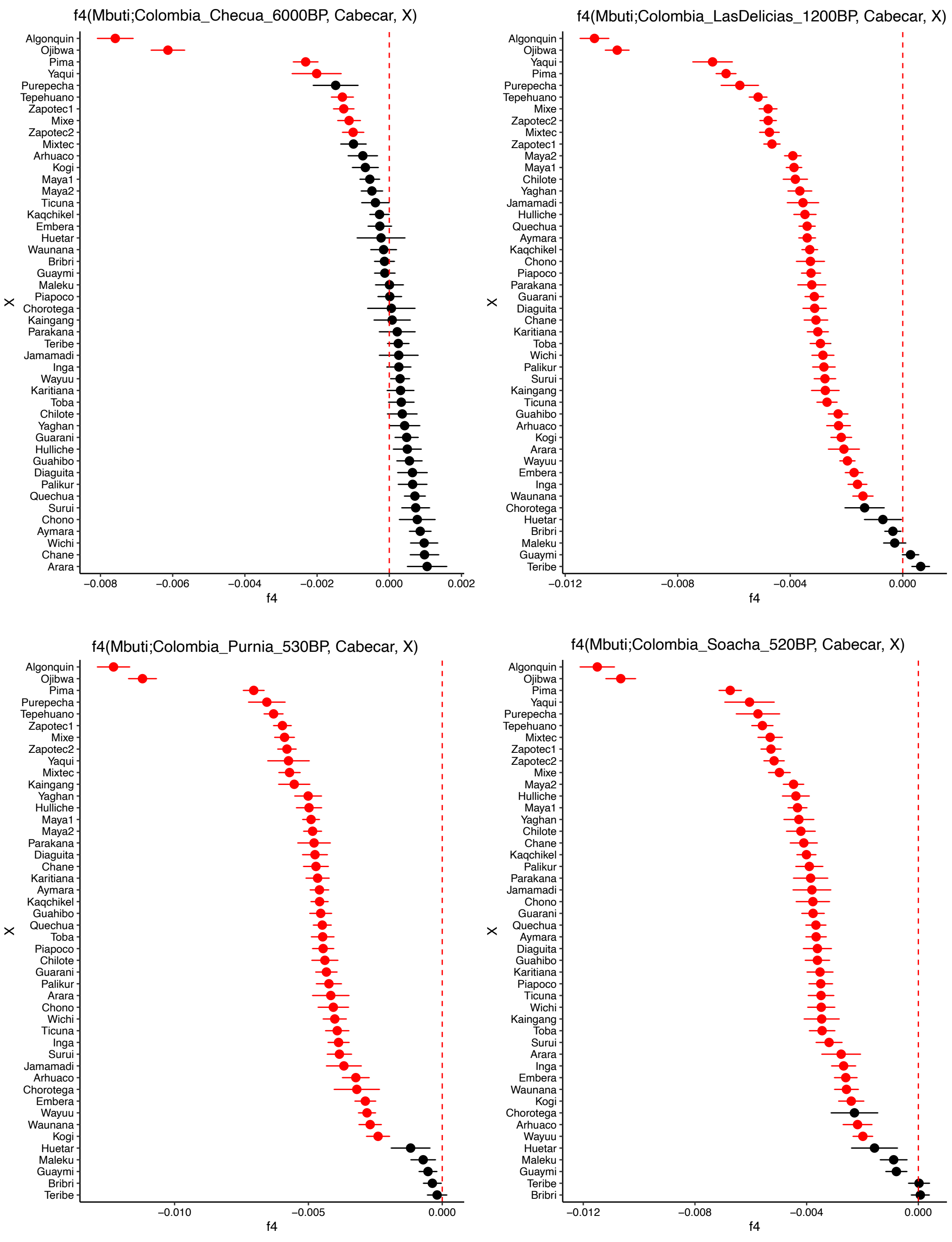

Figure S21: f_4-statistics to test the relative affinity of ancient Colombian individuals compared to Cabecar and other present-day Native American populations. All populations were genotyped on the Illumina dataset, Reich et al. (23). Tests are reported with 1 SE and red symbols indicate Z-scores below -3.

Captions for Supplementary tables

Table S1: Colombian dataset. Meta data of ancient Colombian individuals analyzed in this study.

Table S2.A: f4-outgroup-statistics exploring potential within-group asymmetric relationships. Ancient Colombian populations are compared with ancient populations available in the 1240K.

Table S2.B: f4-outgroup-statistics exploring potential asymmetric relationships within ancient Colombian groups. Ancient Colombian populations are compared with present-day Indigenous populations available in the Illumina dataset Reich et al. (23).

Table S3.A: f4-outgroup statistic describing the affinity of Colombia_Checua_6000BP to Anzick-1. This table shows the results of testing the allele sharing between Colombia_Checua_6000BP and the Anzick-1 individual.

Table S3.B: f4-outgroup statistic describing the affinity of Colombia_Checua_6000BP to the California Channel Islands. Results showcase the allele sharing between both populations.

Table S3.C: f4-outgroup statistic exploring Colombia_Checua's relationship with ancient individuals available in the 1240K panel. This table contains comparisons with all available Native American populations without prior pre-selection.

Table S3.D: f4-statistic describing Colombia_Checua's relationship with modern-day Indigenous individuals. Populations were not pre-selected and are available in the masked Illumina dataset, Reich et al (23).

Table S3.E: f4-outgroup-statistics exploring potential excess affinity of Colombia_Checua_6000BP to Indigenous individuals. All populations are available in the 1240K v54.

Tabel S3.F: f4-outgroup-statistics describing the relationship between Colombia_checua_6000BP and post-2000BP ancient Colombians. Individuals have been grouped by site.

Tabel S3.G: f4-outgroup-statistics exploring the relationship between post-2000BP ancient Colombians, Colombia_Checua_6000BP and Indigenous populations availabe in the 1240K. Results show the allele sharing between these populations. No pre-selection of 1240K populations took place.

Tabel S3.H: f4-outgroup-statistics exploring the relationship between Colombia_Checua_6000BP, post-2000BP ancient Colombians and present-day Indigenous populations. Within all populations European and/or African ancestry has been masked. Populations are available in the Illumina dataset, Reich et al. (23).

Table S4.A: f4-outgroup-statistics exploring the relationship of ancient Colombians with present-day Indigenous populations of the Americas. Non-Indigenous ancestry as been masked and populations are available in the Illumina dataset (Reich et al. (23)).

Table S4.B: f4-outgroup-statistics exploring the relationship between ancient Colombian populations and present-day Indigenous populations (Reich et al. (23)). All populations are available in the masked version of the Illumina dataset.

Table S4.C: f4-outgroup-statistics showing the relationship between ancient Colombian populations, present-day Indigenous Colombians (Arias et al. (60)), and present-day Indigenous populations in the Illumina dataset (Reich et al 2012 (23)). All non-Native American ancestry was masked by the respective authors prior to publication.

Table S4.D: f4-outgroup-statistics exploring the relationship of ancient Colombians and ancient Indigenous populations of the Americas. Results show asymmetric allele sharing of ancient Colombian populations through time.

Table S4.E: f4-outgroup-statistics describing the relationship between ancient Panamanians and ancient Colombians compared to Indigenous populations of the 1240K. Populations from the 1240K have not been preselected and results suggest affinity of post-2000BP Colombians to ancient Isthmians.

Table S4.F: f4-outgroup-statistics describing the relationship between ceramic-age Venezuelans and ancient Colombians compared to ancient Indigenous populations of the 1240K. Ceramic-age Venezuelans have been subdevided into two groups by their respective affinities to ancient Panamanians with Venezuela_LasLocas_Ceramic2 exhibiting higher affinity to ancient Panamanians.

Table S4.G: f4-outgroup-statistics describing the relationship between ancient Indigenous populations of Colombia, ceramic-age Venezuelans, and ancient Panamanians. The test has been conducted for two set ups of ceramic-age Venezuelan grouping; 1) no sub-division, 2) subdivision based on Panamanian affinity.

Table S4.H: f4-outgroup-statistics highlighting the relationship between ancient Indigenous populations of Colombia, ceramic-age Venezuelans, and modern Isthmian populations. Within modern populations non-Native American ancestry has been masked. Venezuelan populations have been subdivided based on their affinity to ancient Isthmian populations.

Table S4.I: f4-outgroup-statistics showcasing the relatinship between ancient Indigenous populations of Colombia, ceramic-age Venezuelans, and ancient Indigenous Caribbeans. Venezuelans have been subdivided into their respective groups based on affinity to ancient Panamanians. This test uses all available ceramic and archaic Caribbean populations.

Table S4.J: f4-outgroup-statistics describing the allele sharing between ancient Indigenous populations of Colombia, ancient Panamanians, and ancient Indigenous Caribbeans. This test utilizes all available ceramic and archaic Caribbean populations of the 1240K v54.

Table S5.A: qpWave analysis. This analysis is testing whether Colombia_Checua_6000BP and Colombia_LagunadelaHerrera_2000BP can be modeled as one ancestry wave.

Table S5.B: qpWave analysis. This test is exploring whether Colombia_Checua_6000BP and Colombia_LasDelicias_1200BP can be modeled as one ancestry wave.

Table S5.C: qpWave analysis. This analysis is testing whether Colombia_Checua_6000BP and Colombia_Purnia_530BP can be modeled as coming from the same ancestry wave.

Table S5.D: qpWave analysis. This test is rejecting H0 for Colombia_Checua_6000BP and Colombia_Soacha_520BP.

Table S5.E: qpWave analysis. These results are proving that H0 can not be rejected for Colombia_LagunadelaHerrera_2000BP and Colombia_LasDelicias_1200BP.

Table S5.F: qpWave analysis. For Colombia_LagunadelaHerrera_2000BP and Colombia_Purnia_530BP a one wave model cannot be rejected.

Table S5.G: qpWave analysis. qpWave is suggesting a potential one-wave model for Colombia_LagunadelaHerrera_2000BP and Colombia_Soacha_520BP.

Table S5.H: qpWave analysis. For Colombia_LasDelicias_1200BP and Colombia_Purnia_530BP we are assuming a one wave model.

Table S5.I: qpWave analysis. We are testing whether Colombia_LasDelicias_1200BP and Colombia_Soacha_520BP can be fitted within a one wave model.

Table S5.J: qpWave analysis. We are testing whether Colombia_Purnia_530BP and Colombia_Soacha_520BP can be fit withing the validity of a one wave model.

Table S6.A: f4-outgroup-statistics describing the relationship between ceramic-age Venezuelans and ancient Colombians. Ancient Venezuelans have been subdivided into Venezuela_LasLocas_Ceramic and Venezuela_LasLocas_Ceramic2 in regards to their affinity to ancient Panamanians with the Venezuela_LasLocas_Ceramic2 showing more allele sharing with Panama.

Table S6.B: f4-outgroup-statistics exploring the asymmetrical relationship of ceramic-age Venezuelans to ancient Panamanians. Individuals of ceramic age Venezuela have been numbered from one to eight as a unique identifier. The results show that Venezuela 6, 5, and 8 share more affinity to ancient Panamanians.

Table S6.C: f4-outgroup-statistics exploring the asymmetrical allele sharing of ceramic-age Venezuelans and ancient Indigenous populations within the 1240K. Populations within the 1240K have not been pre-selected to avoid bias in analysis.

Table S6.D: f4-outgroup-statistics exploring the asymmetrical relationship of grouped ceramic-age Venezuelans to ancient Panamanians. The three Venezuelans that show more affinity to ancient Panamanians have been grouped into Venezuela_LasLocas_Ceramic2. Results confirm their excess allele sharing with Panama compared to Venezuela_LasLocas_Ceramic.

Table S6.E: f4-outgroup-statistics showing the relationship of ceramic-age Venezuelans, ancient Colombians and Indigenous populations of the 1240K. This analysis utilizes an ancient African captured outgroup, which was chosen based on number of available individuals to yield the highest coverage possible. This analysis inquires whether there is a sequencing protocol bias and/or 1240K batch effect.

Table S6.F: f4-outgroup-statistics describing the relationship of two ceramic-age Venezuelan groups and Indigenous individuals of the 1240K. We are mplementing a captured ancient African outgroup to correct for sequencing protocols and 1240K batch effect.

Table S6.G: f4-outgroup-statistics exploring the asymmetrical relationship of ceramic-age Venezuelans to ancient Panamanians. Using a captured ancient African outgroup corrcts for sequencing protocol biases.

Table S6.H: f4-outgroup-statistics exploring the relationship of ancient Panamanians, ancient Colombians and Indigenous populations of the 1240K. With a captured ancient African outgroup we account for sequencing procotol biases.

Table S6.I: f4-outgroup-statistics highlighting the relationship of ancient Colombians and Indigenous populations of the 1240K. Through implementing a captured ancient African outgroup we correct for sequencing protocol biases and 1240K batch effect.

Table S7.A: f4-outgroup-statistics describing the relationship between ancient Colombians, present-day and ancient populations from the Isthmus. Present-day populations have been selected from the masked Illumina dataset from Reich et al. (23).

Table S7.B: f4-outgroup-statistic exploring the allele sharing between different ancient Colombians to modern-day Indigenous Native Americans. All populations are available in the Illumina dataset without pre-selection and non_Native ancestry has been masked by Reich et al. (23).

Table S7.C: f4-outgroup-statistic exploring the allele sharing between ancient Colombians and modern-day Indigenous Native Americans from the whole of Colombia (Arias et al. (60)). Modern-day Colombians have been genotpyed on the HO and non-Native American ancestry has been masked prior to publication.

Table S7.D: f4-outgroup-statistic exploring the relationship of ancient Colombians, present-day Indigenous people (Reich et al. (23)) and modern-day Indigenous Native Americans from Colombia (Arias et al. (60)). All non-Native ancestry was maksed in the repsective presentday datasets.

Table S7.E: f4-outgroup-statistics describing the allele sharing between present-day people from Costa Rica and ancient Colombians. Costa Ricans are part of the masked Illumina dataset from Reich et al. (23).

Table S7.F: f4-outgroup-statistic showing the allele sharing between different ancient Colombians and modern-day Indigenous Native Americans available in the Illumina dataset (Reich et al. (23)). Non-native ancestry was masked and no pre-selection pf specific populations took place.

Table S7.G: f4-outgroup-statistic exploring the relationship between ancient Colombians and present-day Indigenous peoples from Colombia (Arias et al. (60)) and the Isthmus (Reich et al. (23)). Modern-day individuals form Colombia were genotyped on the HO, Isthmian populations on the Illumina panel. Non-Native ancestry was masked.

Table S7.H: f4-outgroup-statistics comparing ancient Colombians with present-day Indigenous Colombians available in the Illumina dataset (Reich et al. (23)). Present-day Colombians were taken from the masked Illumina panel.

Table S7.I: f4-outgoup-statistics comparing post-2000BP Colombians to Cabecar and present-day Indigenous populations (Reich et al. (23)). Cabecar was chosen based on previous f_4-statistic showing the highest allele sharing with ancient Colombians.

Table S7.J: f_4-outgroup-statistics comparing Isthmian populations to present-day Indigenous populations (Reich et al. (23)). All populations are from the masked Illumina dataset.

Table S7.K: f4-outgroup-statistics comparing North Colombian Indigenous populations and Isthmian populations (Reich et al. (23)) against ancient Colombains. All present-day Indigenous populations had their non-Native ancestry masked.

Table S8.A: 1240K meta dataset used in this study. The dataset, version v54, has been restricted to the individuals and populations used in this study.

Table S.8.B: Meta data of present-day Indigenous Panamanians genotyped on the HO. The table was adapted from its respective publication. Data was restricted to individuals used in this study's analyses.

Table S.8.C: Meta data of present-day Indigenous Americans in the Illumina dataset. Native American ancestry has been masked prior to publication by Reich et al. (23). The data table has been adapted from Reich et al. (23) and Capodiferro et al. (20).

Table S.8.D: Meta data of unadmixed present-day Indigenous Americans in the Illumina dataset. This dataset only contains individuals without non-Native ancestry.

Table S.8.E: Meta data of present-day Indigenous Colombians genotyped on the HO. Data has been provided by Arias et al. (60).

Auxiliary Files:
Supplementary Table S1: Meta data table for the generated ancient Colombian genomes.
Supplementary Table S2: f_4-outgroup statistics for inter- and intra-site affinities
Supplementary Table S3: f_4-outgroup statistics for Colombia_Checua_6000BP
Supplementary Table S4: f_4-outgroup statistics for post-2000P ancient Colombians
Supplementary Table S5: *qpWave*-analyses of ancient Colombians
Supplementary Table S6: Comparative f_4-outgroup statistics for Venezuela_LasLocas_Ceramic and ancient Colombians
Supplementary Table S7: f_4-outgroup statistics between ancient Colombians and present-day Indigenous populations
Supplementary Table S8: Comparative meta data used in this study

REFERENCES AND NOTES

1. M. Raghavan, M. Steinrücken, K. Harris, S. Schiffels, S. Rasmussen, M. De Giorgio, A. Albrechtsen, C. Valdiosera, M. C. Ávila-Arcos, A.-S. Malaspinas, A. Eriksson, I. Moltke, M. Metspalu, J. R. Homburger, J. Wall, O. E. Cornejo, J. V. Moreno-Mayar, T. S. Korneliussen, T. Pierre, M. Rasmussen, P. F. Campos, P. de Barros Damgaard, M. E. Allentoft, J. Lindo, E. Metspalu, R. Rodríguez-Varela, J. Mansilla, C. Henrickson, A. Seguin-Orlando, H. Malmström, T. Stafford Jr., S. S. Shringarpure, A. Moreno-Estrada, M. Karmin, K. Tambets, A. Bergström, Y. Xue, V. Warmuth, A. D. Friend, J. Singarayer, P. Valdes, F. Balloux, I. Leboreiro, J. L. Vera, H. Rangel-Villalobos, D. Pettener, D. Luiselli, L. G. Davis, E. Heyer, C. P. E. Zollikofer, M. S. Ponce de León, C. I. Smith, V. Grimes, K.-A. Pike, M. Deal, B. T. Fuller, B. Arriaza, V. Standen, M. F. Luz, F. Ricaut, N. Guidon, L. Osipova, M. I. Voevoda, O. L. Posukh, O. Balanovsky, M. Lavryashina, Y. Bogunov, E. Khusnutdinova, M. Gubina, E. Balanovska, S. Fedorova, S. Litvinov, B. Malyarchuk, M. Derenko, M. J. Mosher, D. Archer, J. Cybulski, B. Petzelt, J. Mitchell, R. Worl, P. J. Norman, P. Parham, B. M. Kemp, T. Kivisild, C. Tyler-Smith, M. S. Sandhu, M. Crawford, R. Villems, D. G. Smith, M. R. Waters, T. Goebel, J. R. Johnson, R. S. Malhi, M. Jakobsson, D. J. Meltzer, A. Manica, R. Durbin, C. D. Bustamante, Y. S. Song, R. Nielsen, E. Willerslev, Genomic evidence for the Pleistocene and recent population history of Native Americans. *Science* **349**, aab3884 (2015).

2. J. V. Moreno-Mayar, B. A. Potter, L. Vinner, M. Steinrücken, S. Rasmussen, J. Terhorst, J. A. Kamm, A. Albrechtsen, A.-S. Malaspinas, M. Sikora, J. D. Reuther, J. D. Irish, R. S. Malhi, L. Orlando, Y. S. Song, R. Nielsen, D. J. Meltzer, E. Willerslev, Terminal Pleistocene Alaskan genome reveals first founding population of Native Americans. *Nature* **553**, 203–207 (2018).

3. M. Sikora, V. V. Pitulko, V. C. Sousa, M. E. Allentoft, L. Vinner, S. Rasmussen, A. Margaryan, P. de Barros Damgaard, C. de la Fuente, G. Renaud, M. A. Yang, Q. Fu, I. Dupanloup, K. Giampoudakis, D. Nogués-Bravo, C. Rahbek, G. Kroonen, M. Peyrot, H. McColl, S. V. Vasilyev, E. Veselovskaya, M. Gerasimova, E. Y. Pavlova, V. G. Chasnyk, P. A. Nikolskiy, A. V. Gromov, V. I. Khartanovich, V. Moiseyev, P. S. Grebenyuk, A. Y. Fedorchenko, A. I. Lebedintsev, S. B. Slobodin, B. A. Malyarchuk, R. Martiniano, M. Meldgaard, L. Arppe, J. U. Palo, T. Sundell, K. Mannermaa, M. Putkonen, V. Alexandersen, C. Primeau, N. Baimukhanov, R. S. Malhi, K.-G. Sjögren, K. Kristiansen, A. Wessman, A. Sajantila, M. M. Lahr, R. Durbin,

R. Nielsen, D. J. Meltzer, L. Excoffier, E. Willerslev, The population history of northeastern Siberia since the Pleistocene. *Nature* **570**, 182–188 (2019).

4. H. Yu, M. A. Spyrou, M. Karapetian, S. Shnaider, R. Radzevičiūtė, K. Nägele, G. U. Neumann, S. Penske, J. Zech, M. Lucas, P. LeRoux, P. Roberts, G. Pavlenok, A. Buzhilova, C. Posth, C. Jeong, J. Krause, Paleolithic to Bronze Age Siberians reveal connections with first Americans and across Eurasia. *Cell* **181**, 1232–1245.e20 (2020).

5. J. V. Moreno-Mayar, L. Vinner, P. de Barros Damgaard, C. de la Fuente, J. Chan, J. P. Spence, M. E. Allentoft, T. Vimala, F. Racimo, T. Pinotti, S. Rasmussen, A. Margaryan, M. Iraeta Orbegozo, D. Mylopotamitaki, M. Wooller, C. Bataille, L. Becerra-Valdivia, D. Chivall, D. Comeskey, T. Devièse, D. K. Grayson, L. George, H. Harry, V. Alexandersen, C. Primeau, J. Erlandson, C. Rodrigues-Carvalho, S. Reis, M. Q. R. Bastos, J. Cybulski, C. Vullo, F. Morello, M. Vilar, S. Wells, K. Gregersen, K. L. Hansen, N. Lynnerup, M. Mirazón Lahr, K. Kjær, A. Strauss, M. Alfonso-Durruty, A. Salas, H. Schroeder, T. Higham, R. S. Malhi, J. T. Rasic, L. Souza, F. R. Santos, A.-S. Malaspinas, M. Sikora, R. Nielsen, Y. S. Song, D. J. Meltzer, E. Willerslev, Early human dispersals within the Americas. *Science* **362**, eaav2621 (2018).

6. C. L. Scheib, H. Li, T. Desai, V. Link, C. Kendall, G. Dewar, P. W. Griffith, A. Mörseburg, J. R. Johnson, A. Potter, S. L. Kerr, P. Endicott, J. Lindo, M. Haber, Y. Xue, C. Tyler-Smith, M. S. Sandhu, J. G. Lorenz, T. D. Randall, Z. Faltyskova, L. Pagani, P. Danecek, T. C. O'Connell, P. Martz, A. S. Boraas, B. F. Byrd, A. Leventhal, R. Cambra, R. Williamson, L. Lesage, B. Holguin, E. Ygnacio-de Soto, J. T. Rosas, M. Metspalu, J. T. Stock, A. Manica, A. Scally, D. Wegmann, R. S. Malhi, T. Kivisild, Ancient human parallel lineages within North America contributed to a coastal expansion. *Science* **360**, 1024–1027 (2018).

7. M. Rasmussen, S. L. Anzick, M. R. Waters, P. Skoglund, M. DeGiorgio, T. W. Stafford Jr., S. Rasmussen, I. Moltke, A. Albrechtsen, S. M. Doyle, G. D. Poznik, V. Gudmundsdottir, R. Yadav, A. S. Malaspinas, S. S. W. V, M. E. Allentoft, O. E. Cornejo, K. Tambets, A. Eriksson, P. D. Heintzman, M. Karmin, T. S. Korneliussen, D. J. Meltzer, T. L. Pierre, J. Stenderup, L. Saag, V. M. Warmuth, M. C. Lopes, R. S. Malhi, S. Brunak, T. Sicheritz-Ponten, I. Barnes, M. Collins, L. Orlando, F. Balloux, A. Manica, R. Gupta, M. Metspalu, C. D. Bustamante, M.

Jakobsson, R. Nielsen, E. Willerslev, The genome of a Late Pleistocene human from a Clovis burial site in western Montana. *Nature* **506**, 225–229 (2014).

8. C. Posth, N. Nakatsuka, I. Lazaridis, P. Skoglund, S. Mallick, T. C. Lamnidis, N. Rohland, K. Nägele, N. Adamski, E. Bertolini, N. Broomandkhoshbacht, A. Cooper, B. J. Culleton, T. Ferraz, M. Ferry, A. Furtwängler, W. Haak, K. Harkins, T. K. Harper, T. Hünemeier, A. M. Lawson, B. Llamas, M. Michel, E. Nelson, J. Oppenheimer, N. Patterson, S. Schiffels, J. Sedig, K. Stewardson, S. Talamo, C.-C. Wang, J.-J. Hublin, M. Hubbe, K. Harvati, A. N. Delaunay, J. Beier, M. Francken, P. Kaulicke, H. Reyes-Centeno, K. Rademaker, W. R. Trask, M. Robinson, S. M. Gutierrez, K. M. Prufer, D. C. Salazar-García, E. N. Chim, L. M. P. Gomes, M. L. Alves, A. Liryo, M. Inglez, R. E. Oliveira, D. V. Bernardo, A. Barioni, V. Wesolowski, N. A. Scheifler, M. A. Rivera, C. R. Plens, P. G. Messineo, L. Figuti, D. Corach, C. Scabuzzo, S. Eggers, P. De Blasis, M. Reindel, C. Méndez, G. Politis, E. Tomasto-Cagigao, D. J. Kennett, A. Strauss, L. Fehren-Schmitz, J. Krause, D. Reich, Reconstructing the deep population history of Central and South America. *Cell* **175**, 1185–1197.e22 (2018).

9. N. Nakatsuka, B. Holguin, J. Sedig, P. E. Langenwalter, J. Carpenter, B. J. Culleton, C. García-Moreno, T. K. Harper, D. Martin, J. Martínez-Ramírez, A. Porcayo-Michelini, V. Tiesler, M. E. Villapando-Canchola, A. Valdes Herrera, K. Callan, E. Curtis, A. Kearns, L. Iliev, A. M. Lawson, M. Mah, S. Mallick, A. Micco, M. Michel, J. N. Workman, J. Oppenheimer, L. Qiu, F. Zalzala, N. Rohland, J. L. Punzo Diaz, J. R. Johnson, D. Reich, Genetic continuity and change among the Indigenous peoples of California. *Nature* **624**, 122–129 (2023).

10. T. Ferraz, X. S. Villagran, K. Nägele, R. Radzevičiūtė, R. B. Lemes, D. C. Salazar-García, V. Wesolowski, M. L. Alves, M. Bastos, A. R. Py-Daniel, H. P. Lima, J. M. Cardoso, R. Estevam, A. Liryo, G. M. Guimarães, L. Figuti, S. Eggers, C. R. Plens, D. M. A. Erler, H. A. V. Costa, I. da Silva Erler, E. Koole, G. Henriques, A. Solari, G. Martin, S. F. S. M. da Silva, R. Kipnis, L. M. Müller, M. Ferreira, J. C. Resende, E. Chim, C. A. da Silva, A. C. Borella, T. Tomé, L. M. P. Gomes, D. B. Fonseca, C. S. da Rosa, J. D. de Moura Saldanha, L. C. Leite, C. M. S. Cunha, S. A. Viana, F. O. Almeida, D. Klokler, H. L. A. Fernandes, S. Talamo, P. De Blasis, S. M. de Souza, C. de Paula Moraes, R. E. Oliveira, T. Hünemeier, A. Strauss, C. Posth, Genomic history of coastal societies from eastern South America. *Nat. Ecol. Evol.* **7**, 1315–1330 (2023).

11. N. Nakatsuka, I. Lazaridis, C. Barbieri, P. Skoglund, N. Rohland, S. Mallick, C. Posth, K. Harkins-Kinkaid, M. Ferry, É. Harney, M. Michel, K. Stewardson, J. Novak-Forst, J. M. Capriles, M. A. Durruty, K. A. Álvarez, D. Beresford-Jones, R. Burger, L. Cadwallader, R. Fujita, J. Isla, G. Lau, C. L. Aguirre, S. LeBlanc, S. C. Maldonado, F. Meddens, P. G. Messineo, B. J. Culleton, T. K. Harper, J. Quilter, G. Politis, K. Rademaker, M. Reindel, M. Rivera, L. Salazar, J. R. Sandoval, C. M. Santoro, N. Scheifler, V. Standen, M. I. Barreto, I. F. Espinoza, E. Tomasto-Cagigao, G. Valverde, D. J. Kennett, A. Cooper, J. Krause, W. Haak, B. Llamas, D. Reich, L. Fehren-Schmitz, A paleogenomic reconstruction of the deep population history of the Andes. *Cell* **181**, 1131–1145.e21 (2020).

12. J. W. Hoopes, O. Fonseca, "Goldwork and Chibchan identity: Endogenous change and diffuse unity in the Isthmo-Colombian area," in *Gold and Power in Ancient Costa Rica, Panama, and Colombia* (Dumbarton Oaks, 2003), pp. 49–89.

13. J. C. Niño Vargas, S. Beckerman, "Universo chibcha, universos chibchas: introducción a la unidad y la diversidad del área istmocolombiana," in *Universos chibchas: Nuevas aproximaciones a la unidad y la diversidad humana del área istmocolombiana.* J. C. Nino Vargas, S. Beckerman eds. (Universidad de los Andes, 2024), pp. 1–59.

14. M. Pache, "Contribution to Chibchan historical linguistics," thesis, University Leiden (2018).

15. M. Pache, Tracing sound change in Nasa Yuwe (western Colombia): Evidence from Andaqui (western Colombia) and Misumalpan languages (Central America). *LIAMES: Línguas Indígenas Americanas* **24**, e024003 (2024).

16. M. Urban, Language classification, language contact and Andean prehistory: The North. *Lang. Linguist. Compass* **15**, e12414 (2021).

17. A. Constenla Umaña, L. Campbell, V. Grondona, "Chibchan languages," in *The Indigenous Languages of South America: A Comprehensive Guide,* V. G. L. Campbell, Ed. (De Gruyter Mouton, 2012), pp. 391–439.

18. A. Constenla Umaña, Sobre el estudio diacrónico de las lenguas chibchenses y su contribución al conocimiento del pasado de sus hablantes. *Boletín Museo del Oro* **3839**, 1356 (1995).

19. L. Casas Vargas, L. M. Romero, W. Usaquén, S. Zea, M. Silva, I. Briceño, A. Gomez, J. V. Rodríguez, Mitochondrial DNA diversity in Prehispanic bone remains on the Eastern Colombian Andes. *Biomedica* **37**, 548–560 (2017).

20. M. R. Capodiferro, B. Aram, A. Raveane, N. Rambaldi Migliore, G. Colombo, L. Ongaro, J. Rivera, T. Mendizábal, I. Hernández-Mora, M. Tribaldos, U. A. Perego, H. Li, C. L. Scheib, A. Modi, A. Gòmez-Carballa, V. Grugni, G. Lombardo, G. Hellenthal, J. M. Pascale, F. Bertolini, G. S. Grieco, C. Cereda, M. Lari, D. Caramelli, L. Pagani, M. Metspalu, R. Friedrich, C. Knipper, A. Olivieri, A. Salas, R. Cooke, F. Montinaro, J. Motta, A. Torroni, J. G. Martín, O. Semino, R. S. Malhi, A. Achilli, Archaeogenomic distinctiveness of the Isthmo-Colombian area. *Cell* **184**, 1706–1723.e24 (2021).

21. P. E. Melton, I. Briceño, A. Gómez, E. J. Devor, J. E. Bernal, M. H. Crawford, Biological relationship between central and South American Chibchan speaking populations: Evidence from mtDNA. *Am. J. Phys. Anthropol.* **133**, 753–770 (2007).

22. M. C. Noguera-Santamaría, C. E. Anderson, D. Uricoechea, C. Durán, I. Briceño-Balcázar, J. Bernal Villegas, Mitochondrial DNA analysis suggests a Chibchan migration into Colombia. *Universitas Scientiarum* **20**, 261–278 (2015).

23. D. Reich, N. Patterson, D. Campbell, A. Tandon, S. Mazieres, N. Ray, M. V. Parra, W. Rojas, C. Duque, N. Mesa, L. F. García, O. Triana, S. Blair, A. Maestre, J. C. Dib, C. M. Bravi, G. Bailliet, D. Corach, T. Hünemeier, M. C. Bortolini, F. M. Salzano, M. L. Petzl-Erler, V. Acuña-Alonzo, C. Aguilar-Salinas, S. Canizales-Quinteros, T. Tusié-Luna, L. Riba, M. Rodríguez-Cruz, M. Lopez-Alarcón, R. Coral-Vazquez, T. Canto-Cetina, I. Silva-Zolezzi, J. C. Fernandez-Lopez, A. V. Contreras, G. Jimenez-Sanchez, M. J. Gómez-Vázquez, J. Molina, A. Carracedo, A. Salas, C. Gallo, G. Poletti, D. B. Witonsky, G. Alkorta-Aranburu, R. I. Sukernik, L. Osipova, S. A. Fedorova, R. Vasquez, M. Villena, C. Moreau, R. Barrantes, D. Pauls, L. Excoffier, G. Bedoya, F. Rothhammer, J.-M. Dugoujon, G. Larrouy, W. Klitz, D.

Labuda, J. Kidd, K. Kidd, A. D. Rienzo, N. B. Freimer, A. L. Price, A. Ruiz-Linares, Reconstructing Native American population history. *Nature* **488**, 370–374 (2012).

24. A. Moreno-Estrada, S. Gravel, F. Zakharia, J. L. McCauley, J. K. Byrnes, C. R. Gignoux, P. A. Ortiz-Tello, R. J. Martínez, D. J. Hedges, R. W. Morris, C. Eng, K. Sandoval, S. Acevedo-Acevedo, P. J. Norman, Z. Layrisse, P. Parham, J. C. Martínez-Cruzado, E. G. Burchard, M. L. Cuccaro, E. R. Martin, C. D. Bustamante, Reconstructing the population genetic history of the Caribbean. *PLOS Genet.* **9**, e1003925 (2013).

25. G. Correal Urrego, *Aguazuque*. *Evidencia de cazadores, recolectores y plantadores en la altiplanicie de la cordillera oriental* (FIAN, 1990).

26. A. M. Groot, *Checua: Una secuencia cultural entre 8 500 y 3 000 años antes del presente* (FIAN, 1992).

27. J. V. Rodríguez, *Tras las huellas de los chibchas de los Andes Orientales de Colombia* (ICANH, 2024).

28. A. Gómez, J. C. Berrio, H. Henry, M. Becerra, R. Marchant, A Holocene pollen record of vegetation change and human impact from Pantano de Vargas, an intra-Andean basin of Duitama, Colombia. *Rev. Palaeobot. Palynol.* **145**, 143–157 (2007).

29. S. M. Broadbent, Reconocimiento arqueológico de la laguna de La Herrera. *Revista colombiana de antropología* **15**, 173–191 (1970).

30. M. E. Delgado Burbano, Mid and Late Holocene population changes at the Sabana de Bogotá (Northern South America) inferred from skeletal morphology and radiocarbon chronology. *Quat. Int.* **256**, 2–11 (2012).

31. M. Delgado, F. Rodríguez, K. Kassadjikova, L. Fehren-Schmitz, A paleogenetic perspective of the Sabana de Bogotá (Northern South America) population history over the Holocene (9000–550 cal BP). *Quat. Int.* **578**, 73–86 (2021).

32. A. M. Boada Rivas, *The Evolution of Social Hierarchy in a Muisca Chiefdom of the Northern Andes of Colombia* (University of Pittsburgh,Universidad de los Andes, 2007).

33. S. Archila, A. M. Groot, J. P. Ospina, M. Mejía, C. Zorro, Dwelling the hill: Traces of increasing sedentism in hunter-gatherers societies at Checua site, Colombia (9500-5052 cal BP). *Quat. Int.* **578**, 102–119 (2021).

34. C. H. Langebaek, *Regional Archaeology in the Muisca territory: A Study of the Fúquene and Susa Valleys* (University of Pittsburgh, Universidad de los Andes, 1995).

35. A. Casas-Vargas, A. Gómez, I. Briceño, M. Díaz-Matallana, J. E. Bernal, J. V. Rodríguez, High genetic diversity on a sample of pre-Columbian bone remains from Guane territories in northwestern Colombia. *Am. J. Phys. Anthropol.* **146**, 637–649 (2011).

36. M. Díaz-Matallana, A. Gómez, I. Briceño, J. V. Rodríguez, Genetic analysis of Paleo-Colombians from Nemocón, Cundinamarca provides insights on the early peopling of northwestern South America. *Revista Acad. Colomb. Ci. Exact.* **40**, 461–483 (2016).

37. S. Mallick, H. Li, M. Lipson, I. Mathieson, M. Gymrek, F. Racimo, M. Zhao, N. Chennagiri, S. Nordenfelt, A. Tandon, P. Skoglund, I. Lazaridis, S. Sankararaman, Q. Fu, N. Rohland, G. Renaud, Y. Erlich, T. Willems, C. Gallo, J. P. Spence, Y. S. Song, G. Poletti, F. Balloux, G. van Driem, P. de Knijff, I. G. Romero, A. R. Jha, D. M. Behar, C. M. Bravi, C. Capelli, T. Hervig, A. Moreno-Estrada, O. L. Posukh, E. Balanovska, O. Balanovsky, S. Karachanak-Yankova, H. Sahakyan, D. Toncheva, L. Yepiskoposyan, C. Tyler-Smith, Y. Xue, M. S. Abdullah, A. Ruiz-Linares, C. M. Beall, A. di Rienzo, C. Jeong, E. B. Starikovskaya, E. Metspalu, J. Parik, R. Villems, B. M. Henn, U. Hodoglugil, R. Mahley, A. Sajantila, G. Stamatoyannopoulos, J. T. S. Wee, R. Khusainova, E. Khusnutdinova, S. Litvinov, G. Ayodo, D. Comas, M. F. Hammer, T. Kivisild, W. Klitz, C. A. Winkler, D. Labuda, M. Bamshad, L. B. Jorde, S. A. Tishkoff, W. S. Watkins, M. Metspalu, S. Dryomov, R. Sukernik, L. Singh, K. Thangaraj, S. Pääbo, J. Kelso, N. Patterson, D. Reich, The Simons genome diversity project: 300 genomes from 142 diverse populations. *Nature* **538**, 201–206 (2016).

38. Y.-Z. Huang, H. Pamjav, P. Flegontov, V. Stenzl, S.-Q. Wen, X.-Z. Tong, C.-C. Wang, L.-X. Wang, L.-H. Wei, J.-Y. Gao, L. Jin, H. Li, Dispersals of the Siberian Y-chromosome haplogroup Q in Eurasia. *Mol. Genet. Genomics* **293**, 107–117 (2018).

39. V. Grugni, A. Raveane, L. Ongaro, V. Battaglia, B. Trombetta, G. Colombo, M. R. Capodiferro, A. Olivieri, A. Achilli, U. A. Perego, J. Motta, M. Tribaldos, S. R. Woodward, L. Ferretti, F. Cruciani, A. Torroni, O. Semino, Analysis of the human Y-chromosome haplogroup Q characterizes ancient population movements in Eurasia and the Americas. *BMC Biol.* **17**, 3 (2019).

40. D. Popli, S. Peyrégne, B. M. Peter, KIN: A method to infer relatedness from low-coverage ancient DNA. *Genome Biol.* **24**, 10 (2023).

41. H. Ringbauer, J. Novembre, M. Steinrücken, Parental relatedness through time revealed by runs of homozygosity in ancient DNA. *Nat. Commun.* **12**, 5425 (2021).

42. J. L. Garcia, "The foods and crops of the Muisca: A dietary reconstruction of the intermediate chiefdoms of Bogotá (Bacatá) and Tunja (Hunza), Colombia," thesis, University of Central Florida (2012).

43. F. M. Olivares, J. M. Madero, A. Casas-Vargas, S. Z. Montoya, D. S. Medellín, L. Gusmão, W. Usaquén, Contrasting the ancestry patterns of three distinct population groups from the northernmost region of South America. *Am. J. Phys. Anthropol.* **173**, 437–447 (2020).

44. D. M. Fernandes, K. A. Sirak, H. Ringbauer, J. Sedig, N. Rohland, O. Cheronet, M. Mah, S. Mallick, I. Olalde, B. J. Culleton, N. Adamski, R. Bernardos, G. Bravo, N. Broomandkhoshbacht, K. Callan, F. Candilio, L. Demetz, K. S. D. Carlson, L. Eccles, S. Freilich, R. J. George, A. M. Lawson, K. Mandl, F. Marzaioli, W. C. McCool, J. Oppenheimer, K. T. Özdogan, C. Schattke, R. Schmidt, K. Stewardson, F. Terrasi, F. Zalzala, C. A. Antúnez, E. V. Canosa, R. Colten, A. Cucina, F. Genchi, C. Kraan, F. La Pastina, M. Lucci, M. V. Maggiolo, B. Marcheco-Teruel, C. T. Maria, C. Martínez, I. París, M. Pateman, T. M. Simms, C. G. Sivoli, M. Vilar, D. J. Kennett, W. F. Keegan, A. Coppa, M. Lipson, R. Pinhasi, D. Reich, A genetic history of the pre-contact Caribbean. *Nature* **590**, 103–110 (2021).

45. K. Nägele, C. Posth, M. Iraeta Orbegozo, Y. Chinique de Armas, S. T. Hernández Godoy, U. M. González Herrera, M. A. Nieves-Colón, M. Sandoval-Velasco, D. Mylopotamitaki, R. Radzeviciute, J. Laffoon, W. J. Pestle, J. Ramos-Madrigal, T. C. Lamnidis, W. C. Schaffer, R.

S. Carr, J. S. Day, C. Arredondo Antúnez, A. Rangel Rivero, A. J. Martínez-Fuentes, E. Crespo-Torres, I. Roksandic, A. C. Stone, C. Lalueza-Fox, M. Hoogland, M. Roksandic, C. L. Hofman, J. Krause, H. Schroeder, Genomic insights into the early peopling of the Caribbean. *Science* **369**, 456–460 (2020).

46. J. W. Hoopes, The emergence of social complexity in the Chibchan world of southern Central America and northern Colombia, AD 300–600. *J. Archaeol. Res.* **13**, 1–47 (2005).

47. J. V. Rodríguez, C. Vargas Vargas, Evolución y tamaño dental en poblaciones humanas de Colombia. *Revista Acad. Colomb. Ci. Exact.* **34**, 423–439 (2010).

48. M. Delgado, Stable isotope evidence for dietary and cultural change over the Holocene at the Sabana de Bogotá region, Northern South America. *Archaeol. Anthropol. Sci.* **10**, 817–832 (2018).

49. D. J. Kennett, M. Lipson, K. M. Prufer, D. Mora-Marín, R. J. George, N. Rohland, M. Robinson, W. R. Trask, H. H. J. Edgar, E. C. Hill, E. E. Ray, P. Lynch, E. Moes, L. O'Donnell, T. K. Harper, E. J. Kate, J. Ramos, J. Morris, S. M. Gutierrez, T. M. Ryan, B. J. Culleton, J. J. Awe, D. Reich, South-to-north migration preceded the advent of intensive farming in the Maya region. *Nat. Commun.* **13**, 1530 (2022).

50. J. Dabney, M. Knapp, I. Glocke, M.-T. Gansauge, A. Weihmann, B. Nickel, C. Valdiosera, N. García, S. Pääbo, J.-L. Arsuaga, M. Meyer, Complete mitochondrial genome sequence of a Middle Pleistocene cave bear reconstructed from ultrashort DNA fragments. *Proc. Natl. Acad. Sci. U.S.A.* **110**, 15758–15763 (2013).

51. N. Rohland, E. Harney, S. Mallick, S. Nordenfelt, D. Reich, Partial uracil–DNA–glycosylase treatment for screening of ancient DNA. *Philos. Trans. R Soc. Lond. B Biol. Sci.* **370**, 20130624 (2015).

52. A. Peltzer, G. Jäger, A. Herbig, A. Seitz, C. Kniep, J. Krause, K. Nieselt, EAGER: Efficient ancient genome reconstruction. *Genome Biol.* **17**, 60 (2016).

53. M. Schubert, S. Lindgreen, L. Orlando, AdapterRemoval v2: Rapid adapter trimming, identification, and read merging. *BMC. Res. Notes* **9**, 88 (2016).

54. H. Li, R. Durbin, Fast and accurate short read alignment with Burrows-Wheeler transform. *Bioinformatics* **25**, 1754–1760 (2009).

55. H. Jónsson, A. Ginolhac, M. Schubert, P. L. F. Johnson, L. Orlando, mapDamage2.0: Fast approximate Bayesian estimates of ancient DNA damage parameters. *Bioinformatics* **29**, 1682–1684 (2013).

56. Q. Fu, M. Meyer, X. Gao, U. Stenzel, H. A. Burbano, J. Kelso, S. Pääbo, DNA analysis of an early modern human from Tianyuan Cave, China. *Proc. Natl. Acad. Sci. U.S.A.* **110**, 2223–2227 (2013).

57. G. Renaud, V. Slon, A. T. Duggan, J. Kelso, Schmutzi: Estimation of contamination and endogenous mitochondrial consensus calling for ancient DNA. *Genome Biol.* **16**, 224 (2015).

58. T. S. Korneliussen, A. Albrechtsen, R. Nielsen, ANGSD: Analysis of next generation sequencing data. *BMC Bioinformatics* **15**, 356 (2014).

59. N. Patterson, A. L. Price, D. Reich, Population structure and eigenanalysis. *PLOS Genet.* **2**, e190 (2006).

60. L. Arias, N. Q. Emlen, S. Norder, N. Julmi, M. Lemus Serrano, T. Chacon, J. Wiegertjes, A. Howard, M. C. B. C. Azevedo, A. Caine, S. Dunn, M. Stoneking, R. Van Gijn, Interpreting mismatches between linguistic and genetic patterns among speakers of Tanimuka (Eastern Tukanoan) and Yukuna (Arawakan). *Interface Focus* **13**, 20220056 (2023).

61. N. Patterson, P. Moorjani, Y. Luo, S. Mallick, N. Rohland, Y. Zhan, T. Genschoreck, T. Webster, D. Reich, Ancient admixture in human history. *Genetics* **192**, 1065–1093 (2012).

62. M. Stuiver, H. A. Polach, Discussion reporting of ^{14}C data. *Radiocarbon* **19**, 355–363 (1977).

63. S. I. Perez, V. Bernal, P. N. Gonzalez, M. Sardi, G. G. Politis, Discrepancy between cranial and DNA data of early Americans: Implications for American peopling. *PLOS ONE* **4**, e5746 (2009).

64. M. Hubbe, W. A. Neves, K. Harvati, Testing evolutionary and dispersion scenarios for the settlement of the new world. *PLOS ONE* **5**, e11105 (2010).

65. S. de Azevedo, A. Nocera, C. Paschetta, L. Castillo, M. González, R. González-José, Evaluating microevolutionary models for the early settlement of the New World: The importance of recurrent gene flow with Asia. *Am. J. Phys. Anthropol.* **146**, 539–552 (2011).

66. R. González-José, M. C. Bortolini, F. R. Santos, S. L. Bonatto, The peopling of America: Craniofacial shape variation on a continental scale and its interpretation from an interdisciplinary view. *Am. J. Phys. Anthropol.* **137**, 175–187 (2008).

67. G. Keyeux, C. Rodas, N. Gelvez, D. Carter, Possible migration routes into South America deduced from mitochondrial DNA studies in Colombian Amerindian populations. *Hum. Biol.* **74**, 211–233 (2002).

68. J. V. Rodríguez Cuenca, *La identificación humana en Colombia. Avances y perspectivas* (Universidad Nacional de Colombia, 2011).

69. J. V. Rodríguez Cuenca, *El Parque Arqueológico de Facatativá* (CAR, Universidad Nacional, 2015).

70. C. H. Langebaek, in *Mercados, poblamiento e integración étnica entre los muiscas* (Banco de la República, 1986).

71. R. Lleras, Los Muiscas en la literatura histórica y antropológica. *Bol. Hist. Antig.* **92**, 307–338 (2005).

72. A. M. Groot, Arqueología y patrimonio: Conocimiento y apropiación social. *Revista Acad. Colomb. Ci. Exact.* 10.18257/raccefyn.30(114).2006.2210 , (2006).

73. A. M. Groot, “Checua: Un aporte para el conocimiento del precerámico de la sabana de Bogotá,” in *Ámbito y Ocupaciones Tempranas de la América Tropical* (Instituto Colombiano de Antropología, 1995), pp. 45–58.

74. A. Minelli, M. Cozzolino, A. Di Nucci, S. Guglielmi, M. Giannantonio, D. D'Amore, E. Pittoni, A. M. Groot, The prehistory of the Colombian territory: The result of the Italian archaeological investigation on the Checua Site (Municipality of Nemocòn, Cundinamarca Department). *J. Biol. Res.-Boll. Soc. Ital. Biol. Sper.* **85**, 10.4081/jbr.2012.4073 (2012).

75. W. A. Neves, M. Hubbe, G. Correal, Human skeletal remains from Sabana de Bogotá, Colombia: A case of Paleoamerican morphology late survival in South America? *Am. J. Phys. Anthropol.* **133**, 1080–1098 (2007).

76. H. M. Pucciarelli, S. I. Perez, G. G. Politis, Early Holocene human remains from the Argentinean Pampas: Additional evidence for distinctive cranial morphology of early South Americans. *Am. J. Phys. Anthropol.* **143**, 298–305 (2010).

77. E. E. R. Braida, Arqueología de rescate, en el barrio las delicias (Bogotá). *Rev. Colomb. Antropol.* **28**, 156–160 (1991).

78. A. Cifuentes Toro, Reseña de un sitio arqueológico en la Mesa de los Santos (Santander). *Bol. Arqueol. FIAN* **4**, 33–40 (2014).

79. S. Mallick, A. Micco, M. Mah, H. Ringbauer, I. Lazaridis, I. Olalde, N. Patterson, D. Reich, The Allen Ancient DNA Resource (AADR) a curated compendium of ancient human genomes. *Sci. Data* **11**, 182 (2024).

80. M. H. Kruschek, “The evolution of the Bogotá chiefdom: A household view,” thesis, University of Pittsburgh (2003).

81. S. Rivas, D. Calderón, C. Marulanda, L. F. Mendoza, G. R. Scott, S. R. Poulson, M. Delgado, Stable isotopes and paleodiet of the ancient inhabitants of Nueva Esperanza: A late Holocene site from Sabana de Bogotá (Colombia). *Int. J. Osteoarchaeol.* **34**, e3244 (2024).

82. J. P. Quintero-Guzmán, El Dorado offerings in Lake Guatavita: A muisca ritual archaeological site. *Latin Am. Antiq.* **35**, 483–499 (2024).

83. P. A. Sánchez-Castañeda, Memory in sacred places: The revitalization process of the Muisca community. *Urban Plan.* **5**, 263–273 (2020).

84. J. V. Rodríguez, *Los chibchas: Hijos del sol, la luna y los Andes. Orígenes de su diversidad* (Universidad Nacional de Colombia, 2011).
